# Consistent gene expression profiles in MexTAg transgenic mouse and wild type mouse asbestos-induced mesothelioma

**DOI:** 10.1186/s12885-015-1953-y

**Published:** 2015-12-18

**Authors:** Cleo Robinson, Ian M. Dick, Michael J. Wise, Andrew Holloway, Dileepa Diyagama, Bruce W. S. Robinson, Jenette Creaney, Richard A. Lake

**Affiliations:** National Centre for Asbestos Related Diseases, School of Medicine and Pharmacology, University of Western Australia, M503, Harry Perkins Institute for Medical Research, QQ Block, QEII Medical Centre, Nedlands, Perth, 6009 Western Australia Australia; Anatomical Pathology, PathWest Laboratory Medicine, J Block, QEII Medical Centre, Hospital Ave, Nedlands, Perth, 6009 Western Australia Australia; School of Chemistry and Biochemistry, University of Western Australia, Crawley, Perth, 6008 Western Australia Australia; Peter MacCallum Institute for Cancer Research, St. Andrew’s Place, Melbourne, 3002 Victoria Australia; Present address: Anatomical Pathology, PathWest Laboratory Medicine, J Block, QEII Medical Centre, Hospital Ave, Nedlands, Perth, 6009 Western Australia Australia

**Keywords:** Mesothelioma, Mouse models of cancer, Asbestos, Gene expression, Expression microarray, SV40 large T antigen

## Abstract

**Background:**

The MexTAg transgenic mouse model of mesothelioma replicates many aspects of human mesothelioma, including induction by asbestos, pathogenicity and response to cytotoxic chemotherapy, despite high levels of the SV40 large T Antigen (TAg) in the mesothelial compartment. This model enables analysis of the molecular events associated with asbestos induced mesothelioma and is utilised here to investigate the molecular dynamics of tumours induced in these mice, using gene expression patterns as a read out.

**Methods:**

Gene expression of MexTAg mesothelioma cell lines bearing a high or low number of copies of the TAg transgene were compared to wild type mouse mesotheliomas and normal mouse mesothelial cells using Affymetrix microarray. These data were then compared to a similar published human microarray study using the same platform.

**Results:**

The main expression differences between transgenic mouse and wild type mouse mesotheliomas occurred for genes involved in cell cycle regulation and DNA replication, as would be expected from overexpression of the TAg oncogene. Quantitative PCR confirmed that E2F and E2F regulated genes were significantly more upregulated in MexTAg mesotheliomas and MexTAg mesothelial cells compared to wild type mesotheliomas. Like human mesothelioma, both MexTAg and wild type mesotheliomas had more genes underexpressed than overexpressed compared to normal mouse mesothelial cells. Most notably, the cdkn2 locus was deleted in the wild type mouse mesotheliomas, consistent with 80 % human mesotheliomas, however, this region was not deleted in MexTAg mesotheliomas. Regardless of the presence of TAg, all mouse mesotheliomas had a highly concordant set of deregulated genes compared to normal mesothelial cells that overlapped with the deregulated genes between human mesotheliomas and mesothelial cells.

**Conclusions:**

This investigation demonstrates that the MexTAg mesotheliomas are comparable with wild type mouse mesotheliomas in their representation of human mesothelioma at the molecular level, with some key gene expression differences that are attributable to the TAg transgene expression. Of particular note, MexTAg mesothelioma development was not dependent on cdkn2 deletion.

**Electronic supplementary material:**

The online version of this article (doi:10.1186/s12885-015-1953-y) contains supplementary material, which is available to authorized users.

## Background

Malignant mesothelioma is an aggressive tumour arising from the mesothelial cells lining the pleura, peritoneum or pericardium. The principal carcinogen associated with malignant mesothelioma is asbestos and several epidemiological data sets and clinical evidence have confirmed its carcinogenic properties [1]. This tumour is a major problem due to its increasing incidence and the lack of effective treatments [2, 3]. Asbestos induces a similar tumour in mice to that seen in humans [4]. This has enabled the transplantation of asbestos-induced mesothelioma into syngeneic mice and has provided useful preclinical information that has guided novel clinical trials [5]. However such models rely on transplantation of clonal tumour cell lines rather than tumours being induced *in situ* by asbestos. These models also suffer from a lack of accurate molecular definition, restricting our capacity to study molecularly targeted therapies [1].

To overcome these limitations we created a murine mesothelioma model in which mesothelioma is reliably induced by the natural carcinogen, asbestos. We achieved this by generating transgenic mice in which the Simian Virus 40 (SV40) large T antigen (TAg) is expressed under the control of the tissue specific mesothelin promoter [6]. In this system, mesothelioma rapidly and reproducibly develops in the peritoneum after instillation of asbestos [7]. Thus, the model represents an anatomically relevant location for mesothelioma and its emergence from mesothelial cells, as well as tumour induction by the known carcinogen. This presented an ideal opportunity to analyse the molecular events associated with asbestos induced mesothelioma. We therefore utilised this system to analyse the molecular dynamics of tumours arising in mice following asbestos exposure, using gene expression patterns as a readout.

At a molecular level mesothelioma is characterised by genetic loss and loss of function of tumour suppressor genes; most commonly cdkn2a and b (encoding p16, p15 and p14 cyclin dependent kinase inhibitor genes); NF2 (neurofibromatosis gene), BAP-1 (BRAC-1 associated protein, an ubiquitase) and LATS-2 [8–10]. Mutations in the tumour suppressors p53 and retinoblastoma (RB) family and the oncogenic ras family occur at a considerably lower frequency in mesothelioma compared to other cancer types [11].

SV40 has been utilized to generate transgenic murine models of various cancer types. In most cases the early coding region of SV40 is targeted to the cell type of interest using a specific promoter, for example the RIP-TAG model of pancreatic cancer uses the rat insulin promoter and the TRAMP model of prostate cancer uses the probasin promoter [12–14]. Malignant transformation in these mice results primarily from the inactivation of the tumour suppressors p53 and RB following binding to TAg [15]. The loss of p53 function makes cells less susceptible to apoptosis [16]. Inactivation of RB results in the activation of the E2F family of transcription factors that induce cell cycle-promoting genes [17]. In the majority of SV40 TAg cancer models, mice develop tumours as they age, for example 100 % of TRAMP mice develop poorly differentiated pancreatic adenocarcinomas by 24 weeks of age [13]. Furthermore, in TRAMP mice the mutation rate is much lower than for carcinogen-induced tumours [18]. Not only does the MexTAg model of mesothelioma development have a strict requirement for the relevant carcinogen, asbestos but a key distinction from other transgenic mesothelioma models, is that interfering spontaneous tumours do not develop in the peritoneum or pleura in the absence of the carcinogen [19].

Three MexTAg mouse lines were developed with different copy numbers of the TAg transgene inserted into the mouse genome [7]. The lines, denoted high TAg (hiTAg), intermediate TAg (intTAg) and single TAg (sTAg) have 100, 32 or single copy of the TAg transgene, respectively. All of the high copy MexTAg mice rapidly develop mesothelioma after asbestos exposure compared to approximately 20–30 % of wild-type mice which develop mesothelioma over a much longer time span [7]. The rate of mesothelioma development is TAg dose dependent: intTAgMexTAg mice have a slower rate than hiTAg mice and the single copy sTAg MexTAg mice have a similar rate to that of wild type mice, and incidence was found to be 90 and 83 % in intTAg and sTAg mice respectively [7]. However, the time to progression after diagnosis is similar between the four models [20]. This suggests that TAg has a role in disease initiation, but that the further development of the mesothelioma is not affected by the presence of TAg. Indeed, MexTAg mesotheliomas respond to cytotoxic chemotherapy in the same way as wild-type mice and patients with mesotheliomas [20].

This study was undertaken to investigate the gene expression differences between wild type and TAg expressing mouse mesotheliomas, to examine the relationship between these models and with human mesothelioma, and in the process to identify gene expression patterns that might be informative of the underlying biology of this tumour.

## Methods

### Mice

Experimental mice were housed in an approved facility, under Animal Ethics Committee approved conditions. Breeding colonies of MexTAg transgenic mice were maintained at The Biomedical Research Facility, University of Western Australia, Perth. Three MexTAg lines were used; the MexTAg 299 h line which has 100 copies of the TAg transgene (hiTAg); the MexTAg 304i line with 32 copies of the transgene (intTAg); and MexTAg 266 s with a single copy (sTAg) [7]. The genotype of each mouse was confirmed by PCR with DNA from murine tails as described previously [7]. C57Bl/6 J wild type mice were purchased from Animal Resources Centre, Perth WA. All animal work was carried out under NHMRC guidelines and with approval from the University of Western Australia Animal Ethics Committee.

### Cell lines

For summary of cell line nomenclature, refer to Table 1.

**Table 1:** Cell line and tumour sample nomenclature and information

Nomenclature	Mouse type	Cell line	TAg copy	No. cell lines arrayed	Median passage No. (range)
WTtu	C57Bl/6 J	Wild type mesothelioma	None	6	5.5 (3–8)
WTn	C57Bl/6 J	Normal mesothelial cells	None	2	3.5 (3–4)
sTAgtu	MexTAg transgenic	TAg mesothelioma	Single copy	3	3 (2–6)
intTAgtu	MexTAg transgenic	TAg mesothelioma	Intermediate copy (32)	3	2 (2–4)
loTAgtu^a^	MexTAg transgenic	TAg mesothelioma	Combined single and intermediate copy tumour samples	(6)	As above 2 rows
hiTAgtu	MexTAg transgenic	TAg mesothelioma	High copy (100)	6	3 (3–4)
hiTAgn	MexTAg transgenic	Normal TAg mesothelial cells	High copy (100)	2	3 (3–4)
Htu^b^	Human	Primary tissue	-	5 cases	n/a
Hn^b^	Human	Non malignant tissue	-	6 cases	n/a

Footnotes

*n* indicates normal mesothelial cells, *tu* indicates a malignant mesothelioma cell line

^a^data set is a combination of sTAGtu and intTAgtu, therefore not in cluster analysis (see Results section)

^b^Roe et al. [22]

### Normal mesothelial cell preparation

Peritoneum of wild type C57Bl/6 J mice or TAg transgenic mice were surgically resected and aseptically cut into small pieces. Mesothelial cells were removed from peritoneum by incubating in 5 ml trypsin (0.25 % in EDTA buffer; Gibco) for 30 min at 37 °C, with gentle agitation. The peritoneum was then discarded and cells were pelleted and cultured in tissue culture flasks in RPMI 1640 medium containing 10 % FCS (Invitrogen), supplemented with 100 U/mL penicillin, 100 μg/mL streptomycin, 2 mM L-glutamine (Gibco). Cells were passaged 2 or 3 times at a ratio of 1:2 or 1:3 prior to preparation of the RNA for the array.

### Mouse mesothelioma cell preparation

Mice were injected with 6 mg of asbestos (IUCC reference sample of Wittenoom Gorge crocidolite) intraperitoneally and euthanized when symptoms of disease were evident, as described previously [7]. At endpoint mice were euthanized and ascites fluid was collected under sterile conditions. Ascites fluid was placed in tissue culture flasks with 10× volume of RPMI (Life Technologies) supplemented with 10 % FCS (Invitrogen, Life Technologies), under 5 % CO_2_ and 95 % humidity. Cell lines were characterised and confirmed to be mesothelioma by electron microscopy, carried out at Anatomical Pathology, PathWest Laboratory Medicine, QEII Medical Centre, Perth WA. Cells were passaged as required.

### Microarray sample preparation and hybridization

Microarray experiments were performed at the Peter MacCallum Cancer Centre, East Melbourne, Australia). Total RNA was extracted from cells at low passage number, as described in Table 1, using TRIZOL reagent (Life Technologies) and further purified by column chromatography using a Qiagen RNeasy spin column (Qiagen). RNA quality and quantity was confirmed using the Agilent 2100 Bioanalyzer and the RNA 6000 Nano Assay kit (Agilent Biotechnologies, Palo Alto, CA). RNA was profiled according to the manufacturer’s protocols on Affymetrix Mouse Genome 430 V2 GeneChips (Affymetrix, Santa Clara CA), which cover approximately 39,000 transcripts. Data was extracted and processed using GeneChip Operating Software (Affymetrix) and gene expression data (*.cel files) were loaded into ‘R’ statistical package [21]. All of the resulting gene expression data (*.cel files) were retained after expression quality was analysed using the QCReport function from the affyQCReport library.

### Human microarray data

Microarray expression data for human mesothelioma was previously described by Roe et al., [22] and E-MTAB-47 data set available from the ArrayExpress http://www.ebi.ac.uk/arrayexpress/ database. Briefly, Affymetrix Human Genome U133 Plus 2.0 GeneChip data was available for 5 mesothelioma patients and normal parietal and visceral pleural samples from six non-cancer patients. There were 18 samples from 11 patients. One was rejected on the grounds of quality control, based on data from the R function QCReport and fitPLM, suggestive of hybridisation anomalies. Of the remaining 17 experiments, 6 are mesothelioma samples and 11 are controls.

### Microarray data analysis

The raw probe set intensities were normalised by the GC robust multi-array average function (gcrma). Data were clustered using the program Cluster and visualised using the program TreeView. Differential gene expression between groups was determined using the makeContrasts and contrasts.fit functions from the linear models for microarray data (limma) package, with the empirical Bayes function eBayes used to assess the significance and Benjamini and Hochberg False Discovery Rate corrected p-values reported. A *p*-value ≤ 0.05 was considered significant. Probe lists were collapsed to gene lists using a Python program based on the annotation files for the Human Genome U133 Plus 2.0 GeneChip provided by Affymetrix (Release 33), with the gene assumed to be represented by the most significant probe. Lists of significant genes were tested for over representation in KEGG pathways [23, 24]. P-values were corrected for multiple comparisons and pathways exhibiting corrected *P*-values of less than 0.05 were considered significant. Murine genes were mapped to their human homologues using a purpose written Python program using data from the database, HOM_AllOrganism.rpt, available from the Jackson Laboratory (ftp://ftp.informatics.jax.org/pub/reports/HOM_All Organism.rpt). The raw probe set intensities were normalised by the GC robust multi-array average function (gcrma) of the linear models for microarray data (Limma) package [25]. The Limma volcano plot function was used to plot log-fold change of individual probe-sets versus log-odds of the differential expression calculated above between the groups.

Array data can be accessed through ArrayExpress accession number: E-MTAB-3988.

### PCR

RNA was extracted from 1 × 10^7^ cultured cells or 100 mg mouse tissues using TRIZOL reagent (Invitrogen) according to manufacturer’s instructions. 5 μg RNA was reverse transcribed into cDNA using Omniscript (Qiagen) according to manufacturer’s instructions. PCR was performed with cDNA using forward and reverse primers as described in Additional file 1: Table S1, using optimised conditions based on the following: 95 °C 5mins, 35 cycles of 95 °C, 60 °C, 72 °C for 30 s at each temperature, followed by a final extension of 5 mins at 72 °C. Relative amounts of the transcripts were normalised to GAPDH transcript in the same cDNA samples.

## Results

### Hierarchical clustering by gene expression segregate MexTAg and wild type mesothelioma

**Fig. 1:**
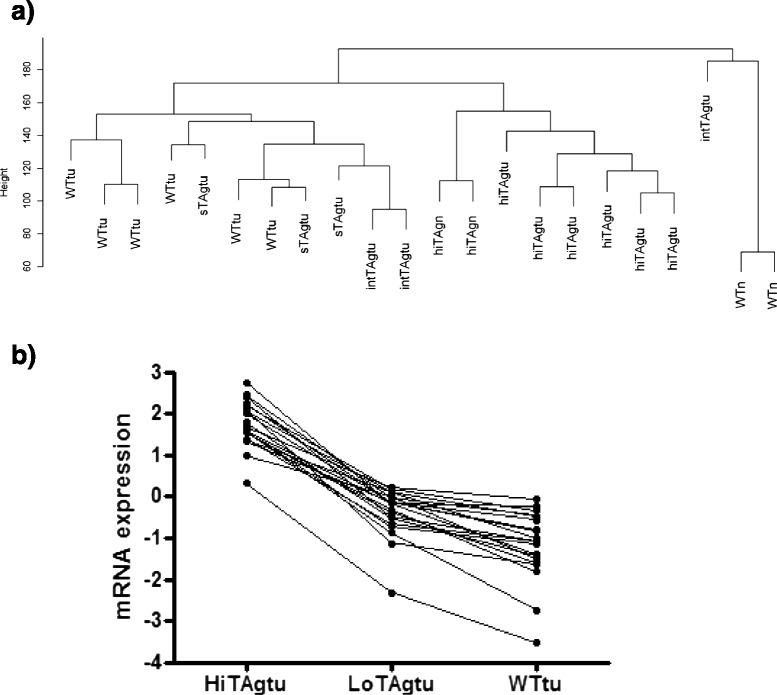
**a** Dendrogram depicting unsupervised clustering of array data for 6 WTtu, 6 hiTAgtu, 3 intTAgtu and 3 sTAgtu together with 2 samples from normal mesothelial cells from wild type mice (WTn) and 2 from high copy MexTAg mice (hiTAgn). **b** Expression of the top 20 most significantly overexpressed genes in a comparison hiTAgtu and WTtu, in WTtu, hiTAgtu and loTAgtu samples

Mesotheliomas were induced in MexTAg transgenic and wild type mice by asbestos instillation into the peritoneum as described previously [7]. Cell lines were generated from the ascites of individual mice and gene expression profiling was carried out on early passage cells. Non-malignant mesothelial cell cultures were also analysed. Details and nomenclature of samples is summarized in Table 1. Unsupervised hierarchical clustering analysis was performed using the entire gene set of expression profiles for a total of 22 samples. The resultant dendrogram shows that the first division separates normal mesothelial cells derived from wild type mice from the wild type mesotheliomas and all samples from TAg transgenic mice (Fig. 1a).

One sample from an intermediate TAg copy number MexTAg tumour cell line (denoted intTAgtu) appeared to be an outlier because it segregated with normal mesothelial cells. However, the large difference in height of the dendrogram arms at the point of segregation for these samples, indicates they have substantially different expression patterns.

The second major division separates both non-malignant mesothelial cells and mesotheliomas from high copy transgenic mouse (hiTAgn and hiTAgtu) from the wild type (WTtu), single TAg (sTAgtu) and intermediate TAg (intTAgtu) tumors. Furthermore, two of the three single copy TAg tumours branched more closely with wild type tumour than with the intermediate copy TAg tumours. This suggests that for high copy MexTAg mice, high TAg expression imparts a stronger influence on gene expression than malignancy.

The intermediate and single copy TAg tumours each had only 3 independent profiles, compared to the 6 hiTAgtu arrays. Therefore we investigated the validity of combining expression patterns of these samples in the context of gene expression differences versus wild type normal mesothelial cells (WTn). We found that 2846 probes were differentially expressed in the single TAg experiment versus WTn, and 2899 probes were differentially expressed in the intermediate TAgtu experiments versus WTn, but there was an overlap of 2087 probes. This amounted to 73 and 75 % for sTAgtu_versus WTn and intTAgtu versus WTn, respectively. This supports the notion that combining sTAgtu and intTAgtu samples, to form a common study set is justified. This is denoted loTAgtu and consists of 6 samples.

### HiTAg mesotheliomas have higher expression of cell cycle regulatory genes compared to wild type mesotheliomas

Comparing the hiTAgtu and WTtu samples, 714 genes were differentially expressed (adjusted *p* value < 0.05). Of these 465 were overexpressed and 245 genes were underexpressed (hiTAgtu:WTtu, Additional file 2: Table S2). Cyclin dependent kinase inhibitor 2a and 2b (CdkN2a and 2b), p107 and N-ras were all significantly over-expressed in TAg tumours relative to wild type tumours. In the loTAg tumours expression of the top 20 of these overexpressed genes were at levels intermediate between hiTAg and WT tumours (Fig. 1b).

Pathway enrichment analysis [24] suggested that differentially expressed genes were associated with cell cycle control and DNA replication, which could be a direct result of the TAg oncogene interaction with cell cycle regulatory proteins (Table 2) Of note, there was enrichment for the MAPK signaling pathway, several genes in the pathway were overexpressed in hiTAgtu but these did not include any of the MAP kinases themselves. Thus suggesting a pathway more favoured by the TAg tumours.

**Table 2:** Pathway enrichment analysis for differentially expressed genes between hiTAgtu and WTtu

Pathway	*p*-value
G1 to S cell cycle control	3.27E-09
Cell cycle	6.93E–08
mRNA processing	9.94E–08
MAPK signalling pathway	1.39E–06
DNA Replication	1.49E–06
Cytoplasmic Ribosomal Proteins	6.98E–05
One carbon metabolism and related	8.94E–04
Protein–protein interactions in the podocyte	0.001066

### Differentially expressed genes are more associated with underexpression in asbestos-induced mouse mesotheliomas compared to normal mesothelial cells, regardless of large T Antigen expression

**Fig. 2:**
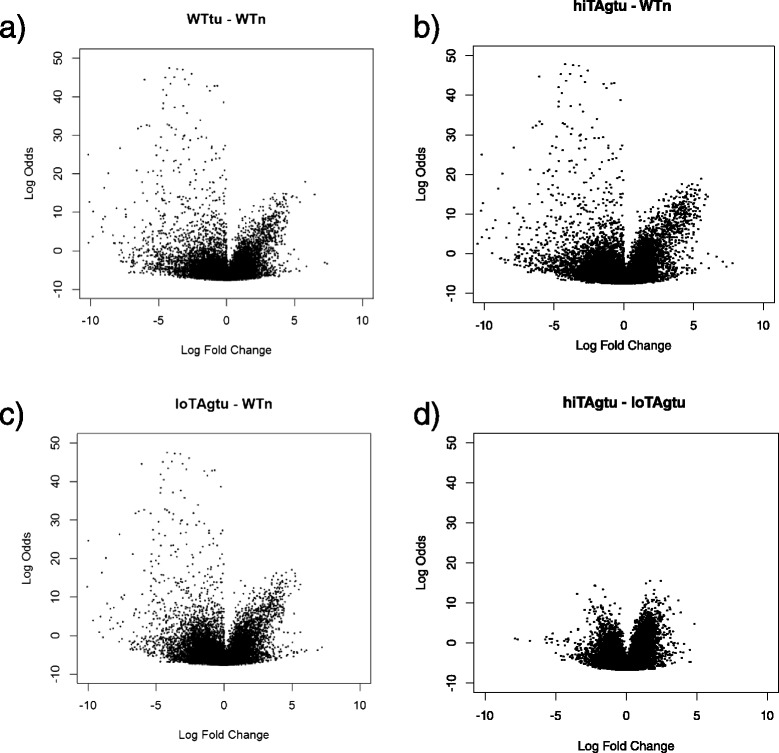
Volcano plots of log-fold change versus log-odds of the differential probe-set expression showing comparative expression levels between sample groups. **a** WTtu and WTn (**b**) hiTAgtu and WTn, (**c**) loTAgtu and WTn and (**d**) loTAgtu and HiTAgtu

Volcano plots were used to illustrate the expression differences of all probes on the array between all three asbestos-induced mesothelioma tumour types and wild type normal mesothelial cells (Fig. 2a, b, c). A similar pattern of over and under expression of probes is seen for each comparison, with a higher number of probes underexpressed than overexpressed in each case. The level of over and under expression is also within a similar range for each comparison. Comparing probe set differences between hiTAgtu and loTAgtu, revealed far less variation in fold change and there was an equal distribution of under and overexpressed probes (Fig. 2d).

Next we looked at the genes that were differentially expressed between each tumour set (ie. tumours generated in wild-type, low or high copy TAg transgenic mice) and normal mesothelial cells, and again found a greater number of genes underexpressed than were overexpressed when compared to normal mesothelial cells.

There were 2203 genes differentially expressed between the asbestos induced wild type tumours (WTtu) and normal mesothelial cells (WTn); 966 genes were significantly overexpressed and 1236 were underexpressed in the WTtu. Cdkn2a (encoding p16) was one of the most significantly under-expressed genes. There was no difference in expression between WTtu and WTn for three other genes commonly deleted in human mesothelioma: Nf2, Bap1 and Lats2. However, as the frequency of deletion of these genes is around 20–50 % of human mesotheliomas, we would not logically expect to find a deletion in the sample size of 6 tumours used in this study.

A comparison of the MexTAg mesotheliomas with normal mesothelial cells from wild type mice (WTn), gave a similar number of genes with expression differences: 2985 genes were differentially expressed between high copy MexTAg tumours (hiTAgtu) and normal mesothelial cells from wild type mouse (WTn) tumours; 1364 genes were overexpressed and 1621 underexpressed.

In a comparison between low copy MexTAg tumours (loTAgtu) and WTn samples, 3126 genes were differentially expressed. Of these, 1301 genes were overexpressed and 1825 genes underexpressed.

### A common set of genes is differentially expressed in asbestos-induced tumours compared to normal mesothelial cells

**Fig. 3:**
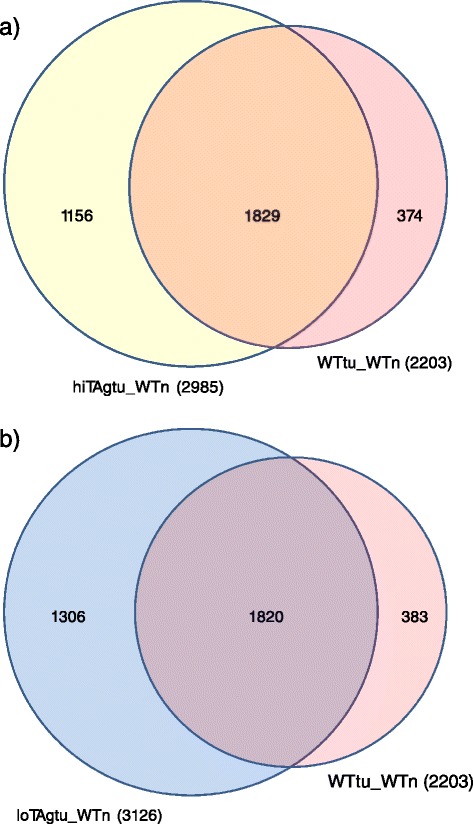
Proportional Venn diagram indicating the number of genes that are common to both sets of differentially expressed gene comparisons (**a**) HiTAgtu_WTn and WTtu_WTn, (**b**) loTAgtu_WTn and WTtu_WTn

Comparison of the genes that were differentially expressed between asbestos induced tumours in either wild type or high copy SV40 TAg transgenic mice and normal mesothelial cells revealed that over 80 % of the genes were common to both models (Fig. 3a). A similar overlap was observed comparing the gene lists differentially expressed between the wild-type tumours and the loTAg tumours (Fig. 3b). The top 20 most significantly differentially expressed genes for each comparison was highly concordant (Table 3). Genes commonly differentially expressed in the asbestos-induced tumours included known cancer and mesothelioma associated genes such as the Aurora kinases A and B, survivin, BRCA1, thymidine kinase, thymidylate synthase, eight members of the mini-chromosome maintenance protein family and topoisomerase 2 alpha (Additional file 3: Table S3). Pathway analysis revealed significant enrichment in these common sets of genes for the DNA replication and cell cycle control pathways (Table 4).

**Table 3:** Top 20 genes most significantly differentially expressed in each tumour sample set compared to wild type normal mesothelial cells

	hiTAgtu_WTn	WTtu_WTn	loTAgtu_WTn	Description
1	Kcna1	Kcna1	Kcna1	Potassium voltage-gated channel, shaker-related subfamily, member 1
2	Prl3a1	Prl3a1	Prl3a1	Prolactin family 3, subfamily a, member 1
3	Kcnc4	Kcnc4	Kcnc4	Potassium voltage gated channel, Shaw-related subfamily, member 4
4	Il17re	Il17re	Il17re	Interleukin 17 receptor E
5	Srd5a2	Srd5a2	Srd5a2	Steroid 5 alpha-reductase 2
6	Prokr2	Prokr2	Prokr2	Prokineticin receptor 2
7	Chi3l1	Chi3l1	Chi3l1	Chitinase 3-like 1
8	Lnx1	Lnx1	Lnx1	Ligand of numb-protein X 1
9	Cmah	Cmah	Cmah	Cytidine monophospho-N-acetylneuraminic acid hydroxylase
10	Emcn	Ppp6r2	Ppp6r2	Endomucin //
11	Dmrtc1a	Emcn	Emcn	DMRT-like family C1a
12	Ppp6r2	Dmrtc1a	Dmrtc1a	Protein phosphatase 6, regulatory subunit 2
13	Cldn10	Cldn10	Cldn10	Claudin 10
14	Selp	Selp	Selp	Selectin, platelet
15	Myo5b	Myo5b	Myo5b	Myosin VB
16	Tmem125	Tmem125	Tmem125	Transmembrane protein 125
17	Slc39a4	Slc39a4	Slc39a4	Solute carrier family 39 (zinc transporter), member 4
18	Fhit	Fhit	Fhit	Fragile histidine triad gene
19	Eef1a2	Sox6	Sox6	Eukaryotic translation elongation factor 1 alpha 2
20	Sox6	Fam19a2	Fam19a2	SRY-box containing gene 6

**Table 4:** Top 10 significantly enriched pathways found in genes commonly differentially expressed between WTtu_WTn and i) hiTAgtu_WTn or ii) loTAgtu_WTn

Pathway	i) hiTAgtu and WTtu	ii) loTAgtu and WTtu
	*p*-value	*p*-value
Cell cycle	0.0	0.0
G1 to S cell cycle control	0.0	0.0
DNA replication	1.760E–29	0.0
Mismatch repair	3.287E–9	1.59E–09
Homologous recombination	2.520E–6	4.66E–05
Purine metabolism	1.287E–5	0.005
mRNA processing	3.887E–5	6.08E–06
Hedgehog signaling pathway	6.695E–4	0.005
Complement activation, classical pathway	0.002	0.003
Nucleotide metabolism	0.004	0.003

### P16, but not other genes commonly deleted in human mesothelioma, is deleted in the wild type mesotheliomas but not in the hiTAg mesotheliomas

**Fig. 4:**
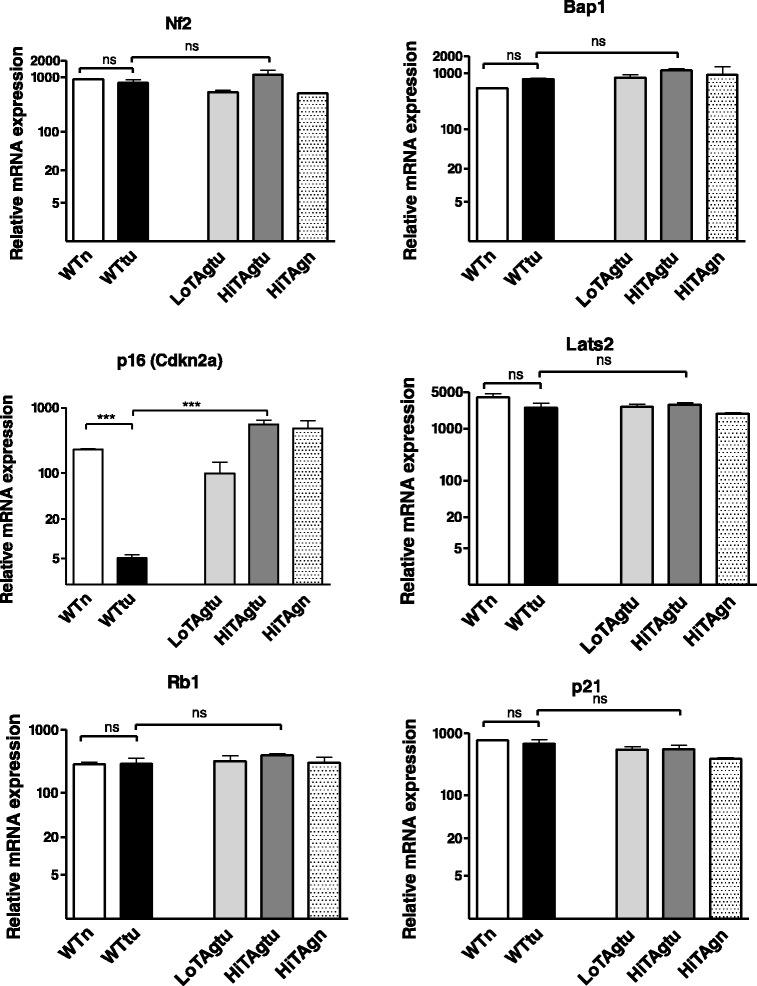
Relative mRNA expression of genes commonly deleted in human mesothelioma in mouse mesotheliomas as indicated. Genes investigated were, NF2, BAP1, p16 and LATS2 and RB (inhibited by Sv40 TAg) and the cyclin dependent inhibitor p21, which has not been reported to be dysregulated in mesothelioma

It was noted earlier that p16 expression levels were higher in TAg tumours and TAg mesothelial cells compared to wild type mesotheliomas, due to deletion of the cdkN2A locus in the latter samples. Thus the expression of the other genes commonly deleted in human mesothelioma, NF2, BAP1 and LATS2 was investigated. However, these three genes were found at similar levels in all three mouse tumour models as well as the normal mesothelial cell lines from wild-type or transgenic mice (Fig. 4). In addition, the expression of Rb was not significantly different between the TAg transgenic and wild-type mouse samples: even though TAg is known to bind and inactivate the RB tumour suppressor gene, this regulation principally occurs by phosphorylation, rather than changes in expression levels. No difference in expression level of a non-TAg targeted or mesothelioma specific cyclin dependent kinase inhibitor, p21, was observed across the sample set.

### E2F targets are overexpressed in TAg tumours and TAg mesothelial cells compared to wild type counterparts

**Fig. 5:**
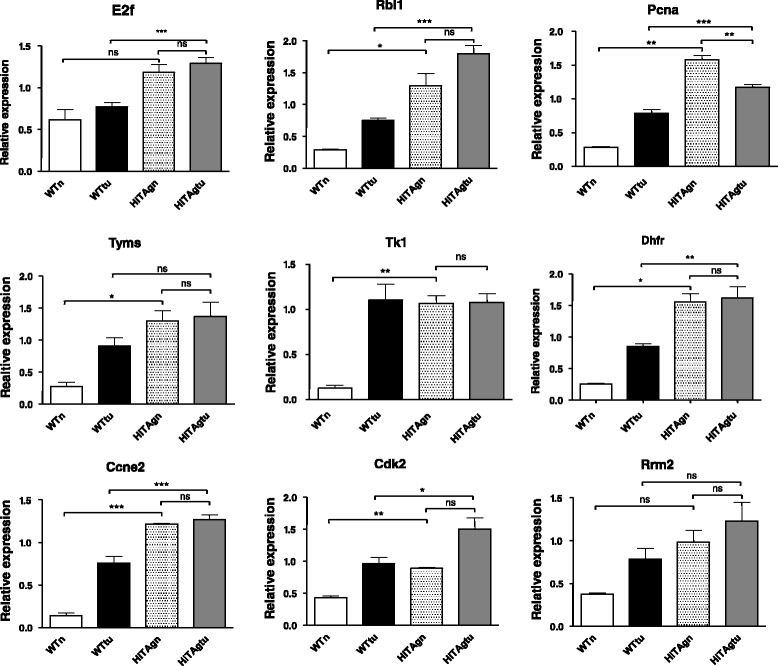
Expression of E2F and E2F regulated genes in WT and MexTAg mesotheliomas and normal mesothelial samples derived from wild type or transgenic mice. Validation quantitative PCRs were carried out using primers described in Methods and genes investigated are as indicated above each graph

The Rb family of proteins influence cell proliferation principally through the actions of the E2F family of transcription factors [17]. Thus, as TAg is known to inhibit and bind Rb and RB family proteins [15], the effect of TAg expression on E2f and E2f targets, was investigated. Seven of the eight E2f targets examined had increased expression levels in the normal mesothelial cells from TAg transgenic mice compared to those from wild-type mice (Fig. 5). Whilst not significant there was also a trend for E2f levels to be increased in normal mesothelial cells from TAg transgenic mice relative to wild type.

Cell cycle pathway genes E2f, Rbl1 (p107), Ccne1 (cyclin E) and cdk2, and DNA synthesis and replication pathway genes, PCNA (proliferating cell nuclear antigen) and DHFR (dihydrofolate reductase) had significantly higher expression in the hiTAgtu samples compared to WTtu (Fig. 5). However, thymidylate synthase (Tyms) and thymidine kinase (TK1) were not significantly different between TAg positive and WT tumours.

### The three mouse mesothelioma models deregulate a common set of genes that is also deregulated in human mesothelioma

A comparison of human mesothelioma with normal human mesothelial cells of the pleura found 1809 differentially expressed genes (Additional file 4: Table S4). Of these, 1106 were overexpressed and 703 were underexpressed. Genes involved in cell cycle, mitosis, replication, DNA repair and anti-apoptosis were overexpressed. However, in this sample set there was no difference in the expression of p16, NF2, BAP1 or LATS2.

**Fig. 6:**
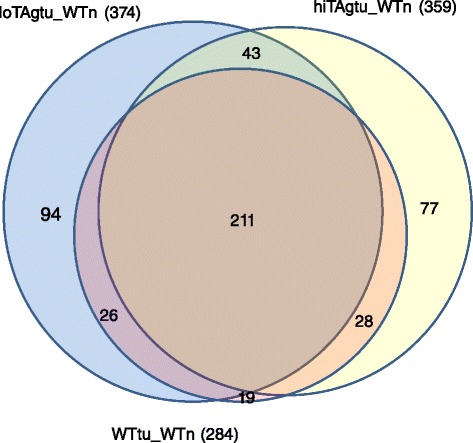
Differentially expressed genes in common between human and mouse mesotheliomas compared to their mesothelial counterpart. Figure shows a proportional Venn diagram indicating the number of overlapping genes that are commonly differentially expressed in each of the three tumour sets, as labeled, compared to the human mesothelioma-human mesothelial differential set (Htu_Hn)

Of the genes in the list of 1809 differentially expressed human mesothelioma genes, 1645 had mouse orthologs. A comparison of the 2203 differentially expressed genes in mesotheliomas from wild type mice versus wild type normal, with this 1645 gene set, found 284 common genes (Fig. 6). Of these 284 wild type mouse to human mesothelioma common genes, 91 % were also in the hiTAgtu to human overlap and 90 % were in the loTAgtu to human overlap (Fig. 6). There were 211 genes significantly differentially expressed common to all 3 mouse models (Additional file 5: Table S5). These 211 genes have functional roles in cell cycle pathway, DNA replication, G1-S phase transition and homologous recombination (Table 5, Additional file 5: Table S5). Gene expression differences identified in the MexTAg cell lines had a greater level of overlap with the differentially expressed genes expression in human mesotheliomas, versus their respective (wild type) normal mesothelial cells: 374 genes for the loTAgtu and 359 genes for the hiTAgtu experiments.

**Table 5:** Significantly enriched pathways between mouse and human mesotheliomas

Pathway	*p* value
Cell_cycle	0
DNA_Replication	3.056866E–11
G1_to_S_cell_cycle_control	2.3044033E–5
Homologous_recombination	7.0286414E–4

## Discussion

We have previously demonstrated that the pathogenesis of mesothelioma in MexTAg transgenic mice exposed to asbestos closely replicates human mesothelioma and it is probably the same disease [19, 20]. While there are a number of other mouse models of mesothelioma available, including transplantation and genetically engineered models, these have a number of disadvantages that render them less suitable as a model or for a molecular study such as this one. Transplantation models are typically in an anatomically irrelevant site making them less suitable for testing novel therapies. A number of genetically engineered models have been generated, such as the Nf2 or p16 knock out mice and Nf2.p53 hemizygous mice [19]. These mice do develop mesothelioma after asbestos exposure, but they are hindered by spontaneous growth of other tumour types. That the MexTAg model is absolutely dependent on asbestos for disease induction and that mesotheliomas are the only cancer to develop, makes this system unique amongst cancer models.

In the current study we showed that the introduction of approximately 100 copies of large TAg into murine mesothelial cells induces a change in the pattern of overall gene expression that sees derived cell lines cluster with the malignant rather than benign mesothelial cells. Such transgenic mesothelial cells exhibit some transformed properties [7] and are able to grow when transplanted into syngeneic mice (unpublished data, [26]. The segregation of mesothelioma tumour cell lines from transgenic mice with low or intermediate numbers of the transgene (loTAgtu and sTAgtu) more closely with mesotheliomas from wild type mice (WTtu) than those that develop in the high copy MexTAg mice (hiTAgtu), suggests the molecular differences caused by the presence of TAg are minor in comparison with the molecular changes that occur as a result of tumorigenesis.

A comparison of the wild type and TAg transgenic tumours showed that a common set of around 1800 genes were differentially expressed compared to wild type normal mesothelial cells, regardless of TAg copy number. However, the magnitude of difference in gene expression levels was directly related to the TAg burden, suggesting a TAg dose dependent affect. This is consistent with the TAg dose dependent rate of induction of mesothelioma by asbestos [7]. The differentially expressed genes we identified are known to be involved in DNA replication and cell cycle control, which could reflect either the increased proliferation rate of tumours or a shared pathway of gene expression changes that occur during tumourigenesis. However, to our knowledge, this is the first study to compare gene expression in a SV40TAg murine cancer model with the same carcinogen induced tumour in both wild type mice and humans. Previous studies of SV40TAg murine cancer models have typically compared gene expression differences with the human cancer counterpart alone. Thus this is a unique study, enabled by the properties of the model itself.

In all six of the wild type mouse mesotheliomas examined p16 was not expressed (above background levels). This is reflective of human mesothelioma whereby deletion of the cdkn2 locus on chromosome 9p21.3 encoding the cyclin dependent kinase inhibitors p16 and p15 is common (up to 80 % of mesothelioma cases) and is thought to be a primary driver of mesothelioma development. Loss of p16 leads to an inability of the cell to phosphorylate RB and activation of the E2F family of transcription factors and increased cell proliferation. Of note, p16 was expressed in all the MexTAg cell lines to a similar level seen in normal wild type mesothelial cells. However, no obvious changes in expression of other genes frequently altered in human mesothelioma such as NF2 and BAP1 was observed in MexTAg tumours. Consistent with the known mechanisms through which TAg exerts its oncogenic effect, TAg expressing cell lines had significantly altered expression of genes downstream of the tumour suppressors p53 and Rb family, including E2F and E2F regulated genes. Thus implying the changes in gene expression observed in the TAg positive mesotheliomas mimic deletion of p16.

For mesotheliomas to develop in MexTAg mice the addition of asbestos is essential. The similarities of MexTAg mesotheliomas at both molecular and functional levels with WT mouse mesotheliomas and their lack of dependence on deletion of p16, suggests that additional, as yet unidentified, genetic changes are required downstream of p16 loss for mesothelioma development. This may be reflective of the multi-step hypothesis of tumour development, for example, where a pattern of hyperplasia, then dysplasia occurs before tumour formation. This hypothesis may explain why tumours develop more rapidly following asbestos exposure in TAg expressing compared to wild type mice and why the rate and incidence of tumour development reflects the TAg transgene dose. Also such a hypothesis explains why there is no difference in the rate or pattern of tumour progression seen between MexTAg and wild type mesothelioma [20].

We made a comparison with a human data set that was selected because it had been analysed on the same platform as our mouse data. Although this comparison was not optimal, due to a number of features, including the fact that pleural as opposed to peritoneal mesotheliomas were compared, it did demonstrate that each of the 3 mouse models had almost identical gene differences in common with the human study. Thus, wild type and TAg transgenic mouse systems could be considered equally good representations of human mesothelioma. Given that the molecular consequences of TAg oncogene expression are well understood, together with our finding that TAg causes the predictable changes in the MexTAg tumours, the model can be reliably used experimentally to represent human disease, keeping TAg effects in mind.

Some of the differentially expressed genes of interest identified in the human study were also identified as genes of interest in our mouse study, for example thymidylate synthase and some of the tumour suppressor genes. Furthermore, we found that the same pathways were significantly altered in both species: including cell cycle regulation, mitosis, DNA repair and apoptosis. Thus, suggesting strong concordance of mesothelioma development across species, to some extent irrespective of TAg expression.

Of note, the MAP kinase pathway was significantly upregulated in the hiTAg mouse mesotheliomas compared to the wild type mouse mesotheliomas, and consistent with this the ras oncogene was also overexpressed in the TAg tumours. While this could be a consequence of large T antigen, activation of this particular cell signalling cascade, has previously been associated with mesothelioma [27]. However, mutations in MAP kinase pathway genes, including RAS and PIK3CA are very rare in human mesothelioma [28].

Broad heterogeneity amongst human tumours is well known and has been aligned with prognosis and response to treatment. However, the steps in tumourigenesis that lead to this heterogeneity are only beginning to be understood and could be a result of germline mutations, somatic mutations, carcinogen exposure as well as environmental and dietary factors. Thus, the heterogeneity amongst experimental mouse tumours would be expected to be somewhat less, due to their homogenous genetic background and tightly regulated environment and dietary intake. Despite this, heterogeneity is still evident in mouse tumour models that are carcinogen or mutation induced [29, 30]. Individual tumours arising from the same tumour cell line transplanted into a group of mice, have related, but distinct gene expression patterns [31]. Moreover, heterogeneity occurs within the same tumour tissue [32].

In this study the method of induction was through a standardised asbestos instillation and although there are some significant differences in gene expression between the TAg and non-TAg mesotheliomas, this is consistent with differences found between cancers developing in models not involving a foreign oncogene. Thus, the MexTAg system can be used to further investigate mesothelioma development at the molecular level, taking into account the known effects of TAg and the findings described here.

## Conclusion

Consistent with our previous observation that the pathogenesis of MexTAg mesothelioma development closely resembles human mesothelioma development, the data presented here further validate the use of the MexTAg model for investigating mesothelioma. In the context of the existence of heterogeneity amongst individual tumours arising from the same tumorigeneic process, we consider the molecular differences described here between the transgenic and the wild type mesotheliomas as small. Furthermore, the differences we identified follow a pattern that is predictable given the presence of the well-studied SV40 large T Antigen. The finding that MexTAg tumours are not reliant on deletion of the cdkN2 locus, suggests that this model could be useful in further investigations of the sequence of molecular events leading to mesothelioma after asbestos exposure.

## Additional files

Additional file 1: Table S1. PCR primers. (DOCX 23 kb) Additional file 2: Table S2. List of differentially expressed genes in ascending order of fold difference in hiTAgtu compared to WTtu. (PDF 135 kb) Additional file 3: Table S3. Genes significantly differentially expressed in hiTAgtu and WTtu sample sets compared to wild type normal mesothelial cells. (PDF 61 kb) Additional file 4: Table S4. List of differentially expressed genes in human mesothelioma compared with normal human mesothelial cells. (PDF 307 kb) Additional file 5: Table S5. List of 211 differentially expressed genes from human array data that were common to the three mouse data sets. (PDF 70 kb)

## References

[1] Robinson BW, Lake RA (2005). Advances in malignant mesothelioma. N Engl J Med.

[2] Olsen NJ, Franklin PJ, Reid A, de Klerk NH, Threlfall TJ, Shilkin K (2011). Increasing incidence of malignant mesothelioma after exposure to asbestos during home maintenance and renovation. Med J Aust.

[3] Le GV, Takahashi K, Park EK, Delgermaa V, Oak C, Qureshi AM (2011). Asbestos use and asbestos-related diseases in Asia: past, present and future. Respirology.

[4] Davis MR, Manning LS, Whitaker D, Garlepp MJ, Robinson BW (1992). Establishment of a murine model of malignant mesothelioma. Int J Cancer.

[5] McCoy MJ, Nowak AK, Lake RA (2009). Chemoimmunotherapy: an emerging strategy for the treatment of malignant mesothelioma. Tissue Antigens.

[6] Urwin D, Lake RA (2000). Structure of the Mesothelin/MPF gene and characterization of its promoter. Mol Cell Biol Res Commun.

[7] Robinson C, van Bruggen I, Segal A, Dunham M, Sherwood A, Koentgen F (2006). A novel SV40 TAg transgenic model of asbestos-induced mesothelioma: malignant transformation is dose dependent. Cancer Res.

[8] Bott M, Brevet M, Taylor BS, Shimizu S, Ito T, Wang L (2011). The nuclear deubiquitinase BAP1 is commonly inactivated by somatic mutations and 3p21.1 losses in malignant pleural mesothelioma. Nat Genet.

[9] de Assis LV, Locatelli J, Isoldi MC (2014). The role of key genes and pathways involved in the tumorigenesis of Malignant Mesothelioma. Biochim Biophys Acta.

[10] Murakami H, Mizuno T, Taniguchi T, Fujii M, Ishiguro F, Fukui T (2011). LATS2 is a tumor suppressor gene of malignant mesothelioma. Cancer Res.

[11] Robinson BW, Musk AW, Lake RA (2005). Malignant mesothelioma. Lancet.

[12] Hanahan D, Wagner EF, Palmiter RD (2007). The origins of oncomice: a history of the first transgenic mice genetically engineered to develop cancer. Genes Dev.

[13] Foster BA, Gingrich JR, Kwon ED, Madias C, Greenberg NM (1997). Characterization of prostatic epithelial cell lines derived from transgenic adenocarcinoma of the mouse prostate (TRAMP) model. Cancer Res.

[14] Colvin EK, Weir C, Ikin RJ, Hudson AL (2014). SV40 TAg mouse models of cancer. Semin Cell Dev Biol.

[15] De Luca A, Baldi A, Esposito V, Howard CM, Bagella L, Rizzo P (1997). The retinoblastoma gene family pRb/p105, p107, pRb2/p130 and simian virus-40 large T-antigen in human mesotheliomas. Nat Med.

[16] Amaral JD, Xavier JM, Steer CJ, Rodrigues CM (2010). The role of p53 in apoptosis. Discov Med.

[17] Di Fiore R, D’Anneo A, Tesoriere G, Vento R (2013). RB1 in cancer: different mechanisms of RB1 inactivation and alterations of pRb pathway in tumorigenesis. J Cell Physiol.

[18] Yadav M, Jhunjhunwala S, Phung QT, Lupardus P, Tanguay J, Bumbaca S (2014). Predicting immunogenic tumour mutations by combining mass spectrometry and exome sequencing. Nature.

[19] Robinson C, Solin J, Lee G, Lake R, Lesterhuis W (2014). Mouse models of mesothelioma: strengths, limitaions and clinical translation. Lung Cancer Manage.

[20] Robinson C, Walsh A, Larma I, O’Halloran S, Nowak AK, Lake RA (2011). MexTAg mice exposed to asbestos develop cancer that faithfully replicates key features of the pathogenesis of human mesothelioma. Eur J Cancer.

[21] Team, RDC (2006). A language and environment for statistical computing.

[22] Roe OD, Anderssen E, Helge E, Pettersen CH, Olsen KS, Sandeck H (2009). Genome-wide profile of pleural mesothelioma versus parietal and visceral pleura: the emerging gene portrait of the mesothelioma phenotype. PLoS One.

[23] Kanehisa M (1997). A database for post-genome analysis. Trends Genet.

[24] Kanehisa M, Goto S (2000). KEGG: kyoto encyclopedia of genes and genomes. Nucleic Acids Res.

[25] Smyth G, Gentleman R, Carey V, Dudoit S, Irizarry R, Huber W (2005). Limma linear models for microarray data. Bioinformatics and computational biology solutions using R and bioconductor.

[26] Cleaver AL, Bhamidipaty K, Wylie B, Connor T, Robinson C, Robinson BW (2014). Long-term exposure of mesothelial cells to SV40 and asbestos leads to malignant transformation and chemotherapy resistance. Carcinogenesis.

[27] Sekido Y (2010). Genomic abnormalities and signal transduction dysregulation in malignant mesothelioma cells. Cancer Sci.

[28] Sekido Y (2013). Molecular pathogenesis of malignant mesothelioma. Carcinogenesis.

[29] Andrechek ER, Cardiff RD, Chang JT, Gatza ML, Acharya CR, Potti A (2009). Genetic heterogeneity of Myc-induced mammary tumors reflecting diverse phenotypes including metastatic potential. Proc Natl Acad Sci U S A.

[30] Wright MH, Robles AI, Herschkowitz JI, Hollingshead MG, Anver MR, Perou CM (2008). Molecular analysis reveals heterogeneity of mouse mammary tumors conditionally mutant for Brca1. Mol Cancer.

[31] Glinsky GV, Krones-Herzig A, Glinskii AB, Gebauer G (2003). Microarray analysis of xenograft-derived cancer cell lines representing multiple experimental models of human prostate cancer. Mol Carcinog.

[32] Gerlinger M, Rowan AJ, Horswell S, Larkin J, Endesfelder D, Gronroos E (2012). Intratumor heterogeneity and branched evolution revealed by multiregion sequencing. N Engl J Med.

