# Supplementary material for: Consistent gene expression profiles in MexTAg transgenic mouse and wild type mouse asbestos-induced mesothelioma
Source: BMC Cancer. 2015 Dec 18;15:983. doi: 10.1186/s12885-015-1953-y (PMC4683914; doi:10.1186/s12885-015-1953-y)
Supplement: Additional file 1: Table S1. — PCR primers. (DOCX 23 kb) [file 12885_2015_1953_MOESM1_ESM.docx]

Additional file 1: Table S1: PCR primers

| Gene | Full name | Forward PCR primer | Reverse PCR primer |
| --- | --- | --- | --- |
| E2f1 | E2F transcription factor 1 | GAGGCTGGATCTGGAGACTG | CCCGGAGATTTCACACCTTTC |
| Cdk2 | cyclin-dependent kinase 2 | CATCTGGAGCAGCATGGAGTC | ATCATCATCACCTGAATCGGGG |
| Ccne2 | cyclin E2 | TCTAAGAGCCACCGTGAGGG | TCAAGGACTATGAAACCTCTGCG |
| RBl1 (p107) | retinoblastoma-like 1 (p107) | TCCCCATGATGCCAATGTCT | AATGGTTGCATATCCTGTCGAA |
| PCNA | proliferating cell nuclear antigen | TACAGCTTACTCTGCGCTCC | TTGGACATGCTGGTGAGGTT |
| Dhfr | dihydrofolate reductase | TAGCGTGAAGGCTGGTAGGA | TTGAACTCGTTCCTGAGCGGA |
| TS | thymidylate synthase | ACGATACAGCCTGAGAGATGA | TCCTCCAAAACACCCTTCCAG |
| TK1 | thymidine kinase 1 | GGGCAAATGCGAGCAGTAAG | AGAATCACCTGAATCTGCCCC |
| Rrm2 | ribonucleotide reductase M2 | AGCAGAGACGAGGGTTTACA | CCTGCTCTATCCTAACGGCG |
