# Supplementary material for: Consistent gene expression profiles in MexTAg transgenic mouse and wild type mouse asbestos-induced mesothelioma
Source: BMC Cancer. 2015 Dec 18;15:983. doi: 10.1186/s12885-015-1953-y (PMC4683914; doi:10.1186/s12885-015-1953-y)
Supplement: Additional file 2: Table S2. — List of differentially expressed genes in ascending order of fold difference in hiTAgtu compared to WTtu. (PDF 135 kb) [file 12885_2015_1953_MOESM2_ESM.pdf]

Gene	logFC	2^	adj_p_val	probeID	genedesc
Fgf7	-6.802	0.008962	0.00784	1422243_at	fibroblast growth factor 7
Postn	-6.516	0.010927	0.0463	1423606_at	periostin, osteoblast specific factor
Ogn	-5.785	0.018136	0.0133	1419663_at	osteoglycin
Thbs2	-5.769	0.018338	0.0197	1450663_at	thrombospondin 2
Serpinf1	-5.49	0.022251	0.0447	1453724_a_at	serine (or cysteine) peptidase inhibitor, clade F, member 1
Suclg2	-4.31	0.050415	0.0155	1427441_a_at	succinate-Coenzyme A ligase, GDP-forming, beta subunit
Emb	-4.218	0.053735	0.0448	1415857_at	embigin
Ctsh	-4.158	0.056017	0.0164	1418365_at	cathepsin H
Ebf1	-4.024	0.061469	0.0496	1416301_a_at	early B cell factor 1
Lrrn4cl	-3.46	0.090873	0.00591	1437771_at	LRRN4 C-terminal like
Loxl1	-3.322	0.099995	0.0088	1451978_at	lysyl oxidase-like 1
Ckb	-3.26	0.104386	0.0451	1455106_a_at	creatine kinase, brain
S100a4	-3.083	0.118012	0.0215	1424542_at	S100 calcium binding protein A4
Leprel2	-2.966	0.127981	0.0116	1449531_at	leprecan-like 2
Mn1	-2.956	0.128871	0.0283	1454867_at	meningioma 1
Fmnl2	-2.869	0.136882	0.0453	1428579_at	formin-like 2
Pcdhb21	-2.858	0.137929	0.0155	1420422_at	protocadherin beta 21
Trps1	-2.854	0.138312	0.00585	1457445_at	trichorhinophalangeal syndrome I (human)
Dnm3os	-2.706	0.153254	0.0356	1427298_at	dynamin 3, opposite strand
AU042950	-2.552	0.170518	0.0195	1458648_at	expressed sequence AU042950
Fam43a	-2.503	0.176409	0.0157	1426734_at	family with sequence similarity 43, member A
Rgs3	-2.401	0.189333	0.0273	1425701_a_at	regulator of G-protein signaling 3
Hic1	-2.37	0.193446	0.0422	1449226_at	hypermethylated in cancer 1
Glis3	-2.346	0.196691	0.00924	1430353_at	GLIS family zinc finger 3
9030617O03	-2.192	0.218848	0.0191	1424226_at	RIKEN cDNA 9030617O03 gene
Wdfy1	-2.173	0.221749	0.0157	1435588_at	WD repeat and FYVE domain containing 1
Zfp799	-2.136	0.22751	0.0182	1437873_at	zinc finger protein 799
B230217C12	-2.035	0.244008	0.014	1428568_at	RIKEN cDNA B230217C12 gene
Mettl7a2	-1.962	0.256672	0.0202	1434150_a_at	methyltransferase like 7A1 /// methyltransferase like 7A2
Srpk2	-1.961	0.25685	0.0294	1431372_at	serine/arginine-rich protein specific kinase 2
Ank3	-1.948	0.259175	0.0148	1425202_a_at	ankyrin 3, epithelial
Col1a2	-1.94	0.260616	0.0362	1446326_at	collagen, type I, alpha 2
Sipa1l2	-1.936	0.26134	0.0471	1434261_at	signal-induced proliferation-associated 1 like 2
Slc46a1	-1.912	0.265724	0.0245	1426714_at	solute carrier family 46, member 1
Cirbp	-1.893	0.269247	0.0067	1416332_at	cold inducible RNA binding protein
Ephx1	-1.892	0.269433	0.016	1422438_at	epoxide hydrolase 1, microsomal
Sgsh	-1.867	0.274143	0.036	1438731_at	N-sulfoglucosamine sulfohydrolase (sulfamidase)
C430003N24	-1.789	0.289373	0.0289	1457508_at	RIKEN cDNA C430003N24 gene
Asrgl1	-1.749	0.297508	0.0274	1424396_a_at	asparaginase like 1
Cyp20a1	-1.749	0.297508	0.0343	1430452_at	cytochrome P450, family 20, subfamily a, polypeptide 1
Mark1	-1.727	0.302079	0.0464	1449630_s_at	MAP/microtubule affinity-regulating kinase 1
C130009A20	-1.723	0.302918	0.00138	1444109_at	RIKEN cDNA C130009A20 gene
Btbd19	-1.718	0.30397	0.0445	1429792_at	BTB (POZ) domain containing 19
Gm11818	-1.716	0.304392	0.0357	1440215_at	predicted gene 11818
Ccdc56	-1.715	0.304603	0.00603	1423840_at	coiled-coil domain containing 56
Kansl1	-1.702	0.30736	0.0089	1457062_at	KAT8 regulatory NSL complex subunit 1
Agxt2l2	-1.681	0.311866	0.0168	1424745_at	alanine-glyoxylate aminotransferase 2-like 2
Mfsd3	-1.675	0.313166	0.00293	1451433_at	major facilitator superfamily domain containing 3
Pttg1ip	-1.67	0.314253	0.00863	1420132_s_at	Pituitary tumor-transforming 1 interacting protein
B4galt7	-1.635	0.32197	0.00375	1426304_x_at	xylosylprotein beta1,4-galactosyltransferase, polypeptide 7 (galactosyltransferase I)
Mbtd1	-1.628	0.323536	0.0302	1441100_at	mbt domain containing 1
Mettl7a1	-1.622	0.324885	0.0395	1434151_at	methyltransferase like 7A1
Orai3	-1.614	0.326691	0.0294	1434064_at	ORAI calcium release-activated calcium modulator 3
Tug1	-1.601	0.329648	0.0402	1426771_at	taurine upregulated gene 1
Spnb2	-1.575	0.335643	0.0328	1419255_at	spectrin beta 2
Tmem198b	-1.572	0.336342	0.0212	1433820_a_at	transmembrane protein 198b
Glrx	-1.571	0.336575	0.0202	1416592_at	glutaredoxin
Rpl23	-1.565	0.337978	0.00864	1422859_a_at	60S ribosomal protein L23-like /// 60S ribosomal protein L23-like /// ribosomal protein L23
Pten	-1.554	0.340565	0.0179	1438454_at	phosphatase and tensin homolog

Tmem205	-1.554	0.340565	0.028	1430421_a_at	transmembrane protein 205
Scp2	-1.546	0.342458	0.0238	1419975_at	Sterol carrier protein 2, liver
Kdm5b	-1.522	0.348203	0.0237	1427142_s_at	lysine (K)-specific demethylase 5B
Strap	-1.512	0.350625	0.0104	1419913_at	Serine/threonine kinase receptor associated protein
Zfp658	-1.51	0.351111	0.0293	1439181_at	zinc finger protein 658
4833412C15	-1.501	0.353308	0.00929	1430360_at	RIKEN cDNA 4833412C15 gene
Tshz3	-1.497	0.354289	0.0395	1435337_at	teashirt zinc finger family member 3
Sco2	-1.485	0.357249	0.0344	1432181_s_at	SCO cytochrome oxidase deficient homolog 2 (yeast)
Lass6	-1.48	0.358489	0.0272	1434418_at	LAG1 homolog, ceramide synthase 6
E430018J23I	-1.462	0.36299	0.0273	1425024_at	RIKEN cDNA E430018J23 gene
Dlx1	-1.453	0.365261	0.0418	1449470_at	distal-less homeobox 1
Tpbg	-1.445	0.367292	0.0311	1423312_at	trophoblast glycoprotein
A130012E19	-1.435	0.369847	0.029	1440671_at	RIKEN cDNA A130012E19 gene
Cpsf6	-1.434	0.370103	0.0273	1437372_at	cleavage and polyadenylation specific factor 6
Ggcx	-1.433	0.37036	0.0419	1423554_at	gamma-glutamyl carboxylase
Epm2aip1	-1.424	0.372678	0.0493	1434105_at	EPM2A (laforin) interacting protein 1
0610007C21	-1.422	0.373195	0.0132	1428380_at	RIKEN cDNA 0610007C21 gene
Jmjd1c	-1.415	0.37501	0.00324	1439998_at	jumonji domain containing 1C
Hrsp12	-1.414	0.37527	0.0332	1428326_s_at	heat-responsive protein 12
Cwc25	-1.394	0.380508	0.0253	1428759_s_at	CWC25 spliceosome-associated protein homolog (S. cerevisiae)
D5Ertd579e	-1.394	0.380508	0.0318	1439691_at	DNA segment, Chr 5, ERATO Doi 579, expressed
2010003O02	-1.393	0.380772	0.0444	1429115_at	RIKEN cDNA 2010003O02 gene
Dock7	-1.382	0.383687	0.00391	1425315_at	dedicator of cytokinesis 7
Pccb	-1.369	0.38716	0.0384	1450969_at	propionyl Coenzyme A carboxylase, beta polypeptide
Gpr137	-1.367	0.387697	0.0109	1436377_at	G protein-coupled receptor 137
AA415038	-1.356	0.390664	0.0273	1457111_at	expressed sequence AA415038
5430416B10	-1.339	0.395295	0.0472	1454558_at	RIKEN cDNA 5430416B10 gene
Prepl	-1.325	0.399149	0.0314	1426345_at	prolyl endopeptidase-like
6230409E13	-1.321	0.400257	0.0395	1440934_at	RIKEN cDNA 6230409E13 gene
Rps3	-1.313	0.402483	0.042	1455600_at	ribosomal protein S3
Zfr	-1.304	0.405002	0.026	1443002_at	zinc finger RNA binding protein
Nus1	-1.296	0.407254	0.0165	1419915_at	Nuclear undecaprenyl pyrophosphate synthase 1 homolog (S. cerevisiae)
Pold4	-1.295	0.407536	0.0155	1427885_at	polymerase (DNA-directed), delta 4
Gns	-1.292	0.408384	0.00636	1446861_at	glucosamine (N-acetyl)-6-sulfatase
Nprl3	-1.289	0.409235	0.0343	1433548_at	nitrogen permease regulator-like 3
Tmem80	-1.27	0.41466	0.0311	1431726_a_at	transmembrane protein 80
Rnf24	-1.269	0.414947	0.0468	1436101_at	ring finger protein 24
Sdc4	-1.268	0.415235	0.0356	1417654_at	syndecan 4
Rnf41	-1.263	0.416677	0.0292	1455763_at	ring finger protein 41
Nudt16	-1.259	0.417833	0.00599	1439884_at	nudix (nucleoside diphosphate linked moiety X)-type motif 16
Nt5c	-1.253	0.419575	0.00819	1417252_at	5',3'-nucleotidase, cytosolic
Itm2c	-1.239	0.423666	0.0271	1415961_at	integral membrane protein 2C
Snord47	-1.232	0.425727	0.0384	1455904_at	growth arrest specific 5 /// small nucleolar RNA, C/D box 47
Pex12	-1.229	0.426613	0.028	1416259_at	peroxisomal biogenesis factor 12
Gmcl1	-1.225	0.427798	0.0273	1438888_at	germ cell-less homolog 1 (Drosophila)
Acly	-1.214	0.431072	0.00195	1439459_x_at	ATP citrate lyase
Rtn4	-1.21	0.432269	0.00895	1435284_at	reticulon 4
2900053A13	-1.207	0.433168	0.03	1453207_at	RIKEN cDNA 2900053A13 gene
Hdac2	-1.189	0.438607	0.0289	1439704_at	histone deacetylase 2
Vps25	-1.188	0.438911	0.0209	1428621_a_at	receptor (calcitonin) activity modifying protein 2 /// vacuolar protein sorting 25 (yeast)
Rad1	-1.187	0.439215	0.0109	1448414_at	RAD1 homolog (S. pombe)
Arfip2	-1.183	0.440435	0.013	1435498_at	ADP-ribosylation factor interacting protein 2
Zcchc7	-1.163	0.446583	0.0179	1438580_at	zinc finger, CCHC domain containing 7
Zfp719	-1.162	0.446893	0.0272	1436594_at	zinc finger protein 719
LOC1008625	-1.161	0.447202	0.0306	1429219_at	IKBKB interacting protein /// inhibitor of nuclear factor kappa-B kinase-interacting protein-like
Laptm4b	-1.154	0.449378	0.0363	1438365_x_at	lysosomal-associated protein transmembrane 4B
Slc38a10	-1.15	0.450625	0.0196	1456241_a_at	solute carrier family 38, member 10
1110032A03	-1.124	0.45882	0.0421	1417211_a_at	RIKEN cDNA 1110032A03 gene
Abhd14a	-1.122	0.459456	0.0215	1441945_s_at	abhydrolase domain containing 14A
Nudt8	-1.114	0.462011	0.0195	1450111_a_at	nudix (nucleoside diphosphate linked moiety X)-type motif 8

Rbfox2	-1.114	0.462011	0.0443	1418245_a_at	RNA binding protein, fox-1 homolog (C. elegans) 2
Arl2	-1.112	0.462652	0.0276	1422549_at	ADP-ribosylation factor-like 2
Rdh5	-1.11	0.463294	0.0071	1422614_s_at	biogenesis of lysosome-related organelles complex-1, subunit 1 /// retinol dehydrogenase 5
Tmem19	-1.11	0.463294	0.0384	1416261_at	transmembrane protein 19
Cst3	-1.1	0.466516	0.0444	1426195_a_at	cystatin C
Tatdn1	-1.094	0.468461	0.0357	1438513_at	TatD DNase domain containing 1
Tnfrsf10b	-1.088	0.470413	0.0421	1422344_s_at	tumor necrosis factor receptor superfamily, member 10b
Ubtd1	-1.075	0.474671	0.0444	1424432_at	ubiquitin domain containing 1
Pdpr	-1.075	0.474671	0.0493	1459869_x_at	pyruvate dehydrogenase phosphatase regulatory subunit
Insig1	-1.07	0.476319	0.0178	1454671_at	insulin induced gene 1
3110056O03	-1.066	0.477641	0.00621	1434658_at	RIKEN cDNA 3110056O03 gene
Mir1931	-1.063	0.478636	0.0149	1446130_at	microRNA 1931
LOC1005050	-1.063	0.478636	0.0262	1439005_x_at	uncharacterized LOC100505062
Fasn	-1.063	0.478636	0.03	1423828_at	fatty acid synthase
Lamb1	-1.062	0.478968	0.0247	1424114_s_at	laminin B1
Ppp1r12a	-1.059	0.479965	0.0303	1429487_at	protein phosphatase 1, regulatory (inhibitor) subunit 12A
Rrp7a	-1.059	0.479965	0.0303	1448141_at	ribosomal RNA processing 7 homolog A (S. cerevisiae)
Glod4	-1.057	0.48063	0.00143	1428421_a_at	glyoxalase domain containing 4
Myh9	-1.053	0.481965	0.0311	1420172_at	Myosin, heavy polypeptide 9, non-muscle
Nudt19	-1.038	0.487002	0.0306	1434216_a_at	nudix (nucleoside diphosphate linked moiety X)-type motif 19
Pgpep1	-1.038	0.487002	0.0327	1418329_at	pyroglutamyl-peptidase I
Arpc4	-1.037	0.48734	0.0401	1423589_at	actin related protein 2/3 complex, subunit 4
Tmed3	-1.035	0.488016	0.0335	1416108_a_at	transmembrane emp24 domain containing 3
D11Wsu47e	-1.034	0.488354	0.0273	1452805_at	DNA segment, Chr 11, Wayne State University 47, expressed
Erlec1	-1.028	0.49039	0.0372	1451449_at	endoplasmic reticulum lectin 1
Znhit2	-1.026	0.49107	0.0306	1416651_at	zinc finger, HIT domain containing 2
1810009A15	-1.023	0.492092	0.00138	1417886_at	RIKEN cDNA 1810009A15 gene
8430408J09I	-1.017	0.494143	0.0476	1430196_at	RIKEN cDNA 8430408J09 gene
Stox2	-1.014	0.495171	0.024	1457778_at	storkhead box 2
Tspyl1	-1.014	0.495171	0.0485	1415908_at	testis-specific protein, Y-encoded-like 1
Ttll1	-1.007	0.49758	0.0332	1436833_x_at	tubulin tyrosine ligase-like 1
Mbnl2	-1.005	0.49827	0.026	1436858_at	muscleblind-like 2
H13	-1.002	0.499307	0.0421	1417287_at	histocompatibility 13
Tada1	-0.993	0.502432	0.0302	1424427_at	transcriptional adaptor 1
Sdf2	-0.988	0.504176	0.0327	1416857_at	stromal cell derived factor 2
Thap4	-0.984	0.505576	0.0153	1458185_at	THAP domain containing 4
Wdr6	-0.983	0.505927	0.0176	1455940_x_at	WD repeat domain 6
Tm9sf3	-0.98	0.50698	0.0456	1428412_at	transmembrane 9 superfamily member 3
Rpl14	-0.962	0.513345	0.0493	1422128_at	ribosomal protein L14
Hadha	-0.957	0.515127	0.0478	1452173_at	hydroxyacyl-Coenzyme A dehydrogenase/3-ketoacyl-Coenzyme A thiolase/enoyl-Coenzyme A hydratase (trifunctional protein), alpha subunit
Phb	-0.956	0.515484	0.0476	1417053_at	RIKEN cDNA 1700071K01 gene /// prohibitin
A230046K03	-0.948	0.518351	0.016	1437719_x_at	RIKEN cDNA A230046K03 gene
Stra13	-0.948	0.518351	0.024	1416553_at	stimulated by retinoic acid 13
H2afy3	-0.947	0.51871	0.0272	1450307_x_at	H2A histone family, member Y3
Cnpy2	-0.945	0.51943	0.0273	1437783_x_at	canopy 2 homolog (zebrafish)
Mpdu1	-0.942	0.520511	0.0463	1416104_at	mannose-P-dolichol utilization defect 1
LOC1008617	-0.931	0.524495	0.0357	1431328_at	uncharacterized LOC100861758
Map3k12	-0.929	0.525222	0.0071	1438908_at	mitogen-activated protein kinase kinase kinase 12
Tbcd	-0.926	0.526316	0.0237	1455801_x_at	tubulin-specific chaperone d
Rpl37	-0.907	0.533293	0.00138	1438291_x_at	predicted pseudogene 13826 /// ribosomal protein L37
Tspan31	-0.907	0.533293	0.0049	1430029_a_at	tetraspanin 31
Noc3l	-0.906	0.533663	0.0355	1437500_at	nucleolar complex associated 3 homolog (S. cerevisiae)
Tex264	-0.905	0.534033	0.0104	1425068_a_at	testis expressed gene 264
Stxbp4	-0.897	0.537002	0.0419	1451063_at	syntaxin binding protein 4
Ywhaz	-0.894	0.53812	0.0277	1436981_a_at	uncharacterized LOC100505062 /// tyrosine 3-monooxygenase/tryptophan 5-monooxygenase activation protein, zeta polypeptide
1200014J11I	-0.891	0.53924	0.0109	1439363_at	RIKEN cDNA 1200014J11 gene
Slc25a20	-0.887	0.540737	0.0432	1423108_at	solute carrier family 25 (mitochondrial carnitine/acylcarnitine translocase), member 20
LOC1005049	-0.881	0.542991	0.00827	1437844_x_at	component of oligomeric golgi complex 1 /// conserved oligomeric Golgi complex subunit 1-like
BC003331	-0.873	0.54601	0.0252	1426089_a_at	cDNA sequence BC003331
Use1	-0.854	0.553249	0.0367	1423817_s_at	unconventional SNARE in the ER 1 homolog (S. cerevisiae)

Cnot2	-0.852	0.554016	0.00398	1456576_x_at	CCR4-NOT transcription complex, subunit 2
Dock1	-0.845	0.556711	0.0057	1452220_at	dedicator of cytokinesis 1
Ttc33	-0.837	0.559806	0.0324	1423958_a_at	tetratricopeptide repeat domain 33
Slc29a3	-0.837	0.559806	0.0473	1455731_at	solute carrier family 29 (nucleoside transporters), member 3
2310004I24I	-0.835	0.560583	0.0317	1450987_a_at	RIKEN cDNA 2310004I24 gene
1110005A03	-0.834	0.560972	0.0273	1451448_a_at	RIKEN cDNA 1110005A03 gene
Yipf3	-0.833	0.561361	0.0384	1451284_at	Yip1 domain family, member 3
Spag7	-0.828	0.56331	0.0355	1433940_at	sperm associated antigen 7
Commd5	-0.816	0.568015	0.0497	1451369_at	COMM domain containing 5
Cdk12	-0.814	0.568803	0.0262	1455964_at	cyclin-dependent kinase 12
Mrps34	-0.812	0.569592	0.0164	1421971_a_at	mitochondrial ribosomal protein S34
Csnk1d	-0.812	0.569592	0.0178	1437690_x_at	casein kinase 1, delta
Rps6kb1	-0.809	0.570777	0.0293	1460705_at	ribosomal protein S6 kinase, polypeptide 1
Cdc40	-0.803	0.573156	0.0242	1457366_at	cell division cycle 40
Mpg	-0.802	0.573554	0.0279	1417571_at	N-methylpurine-DNA glycosylase
Cuedc2	-0.799	0.574747	0.0475	1431145_a_at	CUE domain containing 2
Poldip2	-0.798	0.575146	0.0487	1450727_a_at	polymerase (DNA-directed), delta interacting protein 2
Mrpl11	-0.792	0.577543	0.0189	1437131_x_at	mitochondrial ribosomal protein L11
Taok1	-0.779	0.582771	0.00195	1426358_at	TAO kinase 1
Sap30bp	-0.773	0.585199	0.0155	1449295_at	SAP30-binding protein-like /// SAP30 binding protein
Gosr2	-0.768	0.587231	0.0182	1419371_s_at	golgi SNAP receptor complex member 2
Ict1	-0.767	0.587638	0.00618	1460308_a_at	immature colon carcinoma transcript 1
Cuedc1	-0.765	0.588453	0.0353	1435358_at	CUE domain containing 1
Lmf1	-0.756	0.592136	0.0384	1424259_at	lipase maturation factor 1
Tomm7	-0.755	0.592546	0.0476	1434732_x_at	predicted gene 7226 /// translocase of outer mitochondrial membrane 7 homolog (yeast)
Asb3	-0.754	0.592957	0.0384	1417361_at	ankyrin repeat and SOCS box-containing 3
Ccdc91	-0.746	0.596254	0.026	1449083_at	coiled-coil domain containing 91
Chrac1	-0.73	0.602904	0.0321	1459859_x_at	chromatin accessibility complex 1
Skp1a	-0.711	0.610897	0.0109	1423149_at	S-phase kinase-associated protein 1A
Tax1bp3	-0.711	0.610897	0.0273	1455871_s_at	tax1-binding protein 3-like /// ribosomal protein L13 /// Tax1 (human T cell leukemia virus type I) binding protein 3
Cttn	-0.687	0.621144	0.0306	1433908_a_at	cortactin
Srp68	-0.687	0.621144	0.0443	1433708_at	signal recognition particle 68
Rnf139	-0.683	0.622869	0.0251	1429425_at	ring finger protein 139
Slc35e3	-0.68	0.624165	0.0453	1428805_at	solute carrier family 35, member E3
BC004004	-0.674	0.626767	0.022	1416608_a_at	cDNA sequence BC004004
Txndc17	-0.673	0.627201	0.0245	1439184_s_at	thioredoxin domain containing 17
Lrrc14	-0.671	0.628071	0.0419	1424419_at	leucine rich repeat containing 14
Idnk	-0.666	0.630252	0.0395	1424496_at	idnK gluconokinase homolog (E. coli)
Strada	-0.663	0.631564	0.0468	1438591_at	STE20-related kinase adaptor alpha
Csnk1g2	-0.657	0.634196	0.0149	1423370_a_at	casein kinase 1, gamma 2
Eif3e	-0.645	0.639493	0.0162	1439268_x_at	eukaryotic translation initiation factor 3, subunit E
Srp72	-0.642	0.640824	0.0309	1428876_at	signal recognition particle 72
Eef1g	-0.641	0.641268	0.0211	1417364_at	eukaryotic translation elongation factor 1 gamma
Eef1d	-0.629	0.646624	0.0384	1449506_a_at	eukaryotic translation elongation factor 1 delta (guanine nucleotide exchange protein)
Gm19974	-0.621	0.65022	0.0293	1420304_x_at	predicted gene, 19974
Cct2	-0.617	0.652025	0.0273	1433534_a_at	chaperonin containing Tcp1, subunit 2 (beta)
Rptor	-0.61	0.655197	0.0372	1434619_at	regulatory associated protein of MTOR, complex 1
Cltc	-0.608	0.656106	0.0109	1454626_at	clathrin, heavy polypeptide (Hc)
Ears2	-0.6	0.659754	0.0434	1451471_at	glutamyl-tRNA synthetase 2 (mitochondrial)(putative)
Usp47	-0.594	0.662504	0.0276	1426976_at	ubiquitin specific peptidase 47
Sar1b	-0.558	0.679243	0.0363	1428163_at	SAR1 gene homolog B (S. cerevisiae)
Bloc1s3	-0.551	0.682547	0.0493	1427390_at	biogenesis of lysosome-related organelles complex-1, subunit 3
D10Wsu52e	-0.535	0.690159	0.0253	1423880_at	DNA segment, Chr 10, Wayne State University 52, expressed
Sharpin	-0.509	0.702709	0.0478	1424165_a_at	SHANK-associated RH domain interacting protein
Eef2	-0.5	0.707107	0.0165	1424736_at	eukaryotic translation elongation factor 2
Tcf20	-0.497	0.708579	0.0384	1421910_at	transcription factor 20
Cand1	-0.49	0.712025	0.0302	1428648_at	cullin associated and neddylation disassociated 1
Canx	-0.488	0.713013	0.0321	1415692_s_at	calnexin
Gm6607	-0.45	0.732043	0.0335	1425238_at	40S ribosomal protein S20 pseudogene
Rpl13a	-0.428	0.743291	0.0311	1455001_x_at	ribosomal protein L13A

Naca	-0.422	0.746389	0.0123	1416669_s_at	nascent polypeptide-associated complex alpha polypeptide
Ssr1	-0.42	0.747425	0.0487	1417763_at	signal sequence receptor, alpha
Rps9	-0.412	0.751581	0.00631	1433689_s_at	ribosomal protein S9
Lmf2	-0.4	0.757858	0.0376	1433100_at	lipase maturation factor 2
Rpl38	-0.373	0.772175	0.0241	1433472_x_at	ribosomal protein L38
Rps15a-ps5	-0.322	0.79996	0.028	1453467_s_at	ribosomal protein S15A /// ribosomal protein S15A, pseudogene 5
LOC1008619	0.284	1.217566	0.0492	1439472_at	GCN1 general control of amino-acid synthesis 1-like 1 (yeast) /// translational activator GCN1-like
Sp4	0.316	1.244874	0.043	1421504_at	trans-acting transcription factor 4
Ing5	0.354	1.278099	0.0276	1431134_at	inhibitor of growth family, member 5
Zfp511	0.368	1.290562	0.0195	1434858_x_at	zinc finger protein 511
Tsc1	0.408	1.326845	0.0354	1422043_at	tuberous sclerosis 1
Arid2	0.459	1.374589	0.0468	1432483_at	AT rich interactive domain 2 (ARID, RFX-like)
Gm6340	0.463	1.378405	0.0473	1416698_a_at	CDC28 protein kinase 1b /// predicted pseudogene 6340
Tor1a	0.476	1.390882	0.0153	1426515_a_at	torsin family 1, member A (torsin A)
Ssna1	0.485	1.399586	0.0496	1448643_at	Sjogren's syndrome nuclear autoantigen 1
Rcan3	0.504	1.41814	0.00747	1450450_at	regulator of calcineurin 3
Stard7	0.509	1.423063	0.0252	1449628_s_at	START domain containing 7
Zbtb2	0.51	1.42405	0.0228	1457037_at	zinc finger and BTB domain containing 2
Ankrd11	0.518	1.431969	0.026	1456110_at	ankyrin repeat domain 11
Smad2	0.521	1.43495	0.0367	1420634_a_at	SMAD family member 2
Txnl1	0.548	1.462057	0.0356	1436947_a_at	thioredoxin-like 1
Uck1	0.552	1.466117	0.0403	1424399_at	uridine-cytidine kinase 1
Rad17	0.56	1.474269	0.0343	1448762_at	RAD17 homolog (S. pombe)
Pabpn1	0.572	1.486583	0.0496	1422849_a_at	poly(A) binding protein, nuclear 1
Dennd5b	0.575	1.489677	0.0293	1458146_at	DENN/MADD domain containing 5B
Cops7b	0.582	1.496923	0.0195	1418721_at	COP9 (constitutive photomorphogenic) homolog, subunit 7b (Arabidopsis thaliana)
Ubap2l	0.582	1.496923	0.0272	1433541_a_at	ubiquitin associated protein 2-like
Nt5c3	0.587	1.50212	0.0484	1451050_at	5'-nucleotidase, cytosolic III
Mef2d	0.588	1.503161	0.0354	1437300_at	myocyte enhancer factor 2D
Rnf115	0.618	1.534746	0.0178	1423615_at	ring finger protein 115
B4galt1	0.622	1.539007	0.0186	1418014_a_at	UDP-Gal:betaGlcNAc beta 1,4- galactosyltransferase, polypeptide 1
Snrpd1	0.622	1.539007	0.0315	1416336_s_at	predicted gene 14277 /// small nuclear ribonucleoprotein D1
Fbxw2	0.627	1.54435	0.0273	1432411_a_at	F-box and WD-40 domain protein 2
Pask	0.646	1.564824	0.0366	1450993_at	PAS domain containing serine/threonine kinase
Zc3hav1	0.653	1.572435	0.0311	1436183_at	zinc finger CCCH type, antiviral 1
Luc7l2	0.655	1.574616	0.028	1423251_at	LUC7-like 2 (S. cerevisiae)
Pogz	0.667	1.587768	0.0325	1434650_at	pogo transposable element with ZNF domain
Fbxl14	0.675	1.596597	0.0187	1417407_at	F-box and leucine-rich repeat protein 14
Wrnip1	0.677	1.598812	0.0419	1448376_at	Werner helicase interacting protein 1
Sfmbt1	0.683	1.605475	0.00328	1455412_at	Scm-like with four mbt domains 1
Lrrc57	0.685	1.607702	0.0228	1430057_s_at	leucine rich repeat containing 57
Phf2	0.687	1.609932	0.0228	1421067_a_at	PHD finger protein 2
Zfp472	0.699	1.623379	0.0134	1425058_at	zinc finger protein 472
Fam82a2	0.701	1.625631	0.0215	1452704_at	family with sequence similarity 82, member A2
Lrrc40	0.708	1.633538	0.0228	1448720_at	leucine rich repeat containing 40
Map3k3	0.708	1.633538	0.0382	1436522_at	mitogen-activated protein kinase kinase kinase 3
Zfp653	0.709	1.634671	0.0425	1435481_at	zinc finger protein 653
Ltn1	0.71	1.635804	0.0138	1427950_at	listerin E3 ubiquitin protein ligase 1
Ube2l3	0.71	1.635804	0.0165	1448880_at	predicted gene 10015 /// predicted gene 10145 /// predicted gene 10705 /// predicted gene 5858 /// ubiquitin-conjugating enzyme E2 L3-like /// ubiquitin-conjugating enzyme E2L 3
R3hdm1	0.711	1.636938	0.0464	1458539_at	R3H domain 1 (binds single-stranded nucleic acids)
Clcn7	0.712	1.638073	0.0384	1450408_at	chloride channel 7
Qk	0.714	1.640346	0.0113	1429318_a_at	quaking
Mkln1	0.717	1.64376	0.0472	1436096_at	muskelin 1, intracellular mediator containing kelch motifs
Opa1	0.722	1.649467	0.0071	1418768_at	optic atrophy 1
Ccdc14	0.728	1.656341	0.0351	1435654_at	coiled-coil domain containing 14
Mapkapk5	0.731	1.659789	0.0471	1427790_at	a disintegrin and metallopeptidase domain 1a /// MAP kinase-activated protein kinase 5
Rbm18	0.732	1.66094	0.00585	1420607_at	RNA binding motif protein 18
Tnrc18	0.734	1.663244	0.0223	1427267_at	trinucleotide repeat containing 18
Cep192	0.746	1.677136	0.0468	1428727_at	centrosomal protein 192
Actl6a	0.751	1.682959	0.0475	1416569_at	actin-like 6A

Cept1	0.752	1.684126	0.0272	1416471_at	choline/ethanolaminephosphotransferase 1
Uap1	0.759	1.692317	0.00611	1416743_at	UDP-N-acetylglucosamine pyrophosphorylase 1
Coro7	0.766	1.700548	0.00141	1428148_s_at	coronin 7
Bcas2	0.77	1.70527	0.00872	1418234_s_at	breast carcinoma amplified sequence 2
Pggt1b	0.771	1.706452	0.0402	1429769_at	protein geranylgeranyltransferase type I, beta subunit
Exosc10	0.779	1.715941	0.0431	1431832_x_at	exosome component 10
Hcfc1	0.787	1.725483	0.0273	1421972_s_at	host cell factor C1
2810408B13	0.79	1.729074	0.0397	1422172_x_at	RIKEN cDNA 2810408B13 gene
C2cd2l	0.791	1.730273	0.0343	1428095_a_at	C2 calcium-dependent domain containing 2-like
Gmeb2	0.793	1.732674	0.0478	1454818_at	glucocorticoid modulatory element binding protein 2
Dsn1	0.795	1.735077	0.0266	1417139_at	DSN1, MIND kinetochore complex component, homolog (S. cerevisiae)
Dgcr2	0.801	1.742308	0.0092	1421809_at	DiGeorge syndrome critical region gene 2
Mynn	0.801	1.742308	0.0272	1421333_a_at	myoneurin
Mapk14	0.803	1.744725	0.0338	1416704_at	mitogen-activated protein kinase 14
Xpo4	0.804	1.745935	0.00871	1430797_at	exportin 4
Hltf	0.804	1.745935	0.0314	1425996_a_at	helicase-like transcription factor
Agk	0.805	1.747146	0.0279	1431655_a_at	acylglycerol kinase
Asxl1	0.807	1.74957	0.0343	1458380_at	additional sex combs like 1
Carhsp1	0.81	1.753211	0.0247	1415976_a_at	calcium regulated heat stable protein 1
Pcna	0.811	1.754427	0.00786	1417947_at	proliferating cell nuclear antigen
Isca2	0.811	1.754427	0.0478	1424154_a_at	iron-sulfur cluster assembly 2 homolog (S. cerevisiae)
Rnf8	0.813	1.756861	0.0189	1451082_at	FtsJ methyltransferase domain containing 2 /// ring finger protein 8
Crkl	0.823	1.769081	0.0116	1421954_at	v-crk sarcoma virus CT10 oncogene homolog (avian)-like
Trappc8	0.831	1.778918	0.0057	1428969_at	trafficking protein particle complex 8
Pygo2	0.832	1.780151	0.0415	1428249_at	pygopus 2
Sp1	0.836	1.785094	0.0104	1448994_at	trans-acting transcription factor 1
Paip2b	0.837	1.786332	0.0463	1451125_at	poly(A) binding protein interacting protein 2B
Arrdc1	0.845	1.796265	0.0225	1424578_at	arrestin domain containing 1
Jund	0.847	1.798757	0.0315	1449117_at	Jun proto-oncogene related gene d
Atxn7	0.85	1.802501	0.0168	1426287_at	ataxin 7
Zfp131	0.852	1.805001	0.0344	1428616_at	zinc finger protein 131
Raver1-fdx1l	0.853	1.806253	0.0278	1427111_s_at	ribonucleoprotein, PTB-binding 1 /// Raver1-Fdx1l readthrough
Mbd2	0.854	1.807505	0.0128	1425803_a_at	methyl-CpG binding domain protein 2
Cdkn2aipnl	0.854	1.807505	0.0408	1440715_s_at	CDKN2A interacting protein N-terminal like
Trmt2b	0.856	1.810013	0.0246	1433672_at	TRM2 tRNA methyltransferase 2 homolog B (S. cerevisiae)
Tpx2	0.857	1.811268	0.0429	1428104_at	TPX2, microtubule-associated protein homolog (Xenopus laevis)
Stxbp3a	0.857	1.811268	0.0463	1416653_at	syntaxin binding protein 3A
Ttbk2	0.86	1.815038	0.0161	1440734_at	tau tubulin kinase 2
6330564D18	0.862	1.817556	0.00895	1460624_at	RIKEN cDNA 6330564D18 gene
Naa38	0.867	1.823866	0.0297	1436107_at	N(alpha)-acetyltransferase 38, NatC auxiliary subunit
Ezh2	0.87	1.827663	0.028	1416544_at	enhancer of zeste homolog 2 (Drosophila)
Arhgap11a	0.872	1.830198	0.0384	1438434_at	Rho GTPase activating protein 11A
Zfp944	0.876	1.83528	0.03	1425085_at	zinc finger protein 944
Usp1	0.88	1.840375	0.0273	1451080_at	ubiquitin specific peptidase 1
Pms1	0.88	1.840375	0.0302	1438071_at	postmeiotic segregation increased 1 (S. cerevisiae)
Arid1a	0.884	1.845485	0.0345	1448709_at	AT rich interactive domain 1A (SWI-like)
Stag2	0.888	1.850609	0.0278	1450396_at	stromal antigen 2
Rnf168	0.891	1.854461	0.00784	1455585_at	ring finger protein 168
Wnk1	0.891	1.854461	0.0417	1437888_at	WNK lysine deficient protein kinase 1
Trmt2a	0.893	1.857034	0.00143	1453995_a_at	TRM2 tRNA methyltransferase 2 homolog A (S. cerevisiae)
Tmem189	0.895	1.85961	0.00784	1424411_at	transmembrane protein 189
Stk4	0.895	1.85961	0.0198	1421107_at	serine/threonine kinase 4
Ckap2l	0.896	1.860899	0.00811	1453769_at	cytoskeleton associated protein 2-like
Atp11b	0.896	1.860899	0.0418	1451388_a_at	ATPase, class VI, type 11B
Prdm2	0.899	1.864773	0.028	1453068_at	PR domain containing 2, with ZNF domain
3110001I22I	0.91	1.879045	0.0197	1429165_at	RIKEN cDNA 3110001I22 gene
Plekha8	0.91	1.879045	0.0377	1454819_at	pleckstrin homology domain containing, family A (phosphoinositide binding specific) member 8
Luzp1	0.912	1.881652	0.0456	1448352_at	leucine zipper protein 1
Ss18	0.918	1.889494	0.0463	1419361_at	synovial sarcoma translocation, Chromosome 18
Etnk1	0.922	1.89474	0.00863	1454633_at	ethanolamine kinase 1

1700021K19	0.923	1.896054	0.0326	1428243_at	RIKEN cDNA 1700021K19 gene
Gon4l	0.928	1.902637	0.0197	1424385_at	gon-4-like (C.elegans)
Ankhd1	0.928	1.902637	0.0488	1448008_at	ankyrin repeat and KH domain containing 1
Dhfr	0.93	1.905276	0.0476	1419172_at	dihydrofolate reductase
Cdc25c	0.941	1.919859	0.0488	1422252_a_at	cell division cycle 25C
Ube2t	0.943	1.922522	0.0444	1422462_at	ubiquitin-conjugating enzyme E2T (putative)
Zfand2a	0.943	1.922522	0.0455	1415940_at	zinc finger, AN1-type domain 2A
Ankrd13c	0.945	1.925189	0.0134	1433521_at	ankyrin repeat domain 13c
Nup160	0.945	1.925189	0.0403	1418530_at	nucleoporin 160
Synj1	0.946	1.926524	0.0468	1436334_at	synaptojanin 1
Zranb3	0.957	1.941269	0.0087	1453617_at	zinc finger, RAN-binding domain containing 3
Hspa14	0.957	1.941269	0.0207	1448586_at	heat shock protein 14
Phip	0.957	1.941269	0.0296	1425721_at	pleckstrin homology domain interacting protein
Sfswap	0.958	1.942615	0.0262	1454683_at	splicing factor, suppressor of white-apricot homolog (Drosophila)
Pms2	0.958	1.942615	0.0262	1423539_at	postmeiotic segregation increased 2 (S. cerevisiae)
Arl13b	0.959	1.943962	0.0149	1437021_at	ADP-ribosylation factor-like 13B
Trip6	0.965	1.952064	0.00682	1449041_a_at	thyroid hormone receptor interactor 6
Mtbp	0.965	1.952064	0.0367	1448638_at	Mdm2, transformed 3T3 cell double minute p53 binding protein
Prim1	0.966	1.953417	0.0247	1449061_a_at	DNA primase, p49 subunit
2700097O09	0.969	1.957483	0.00784	1428810_at	RIKEN cDNA 2700097O09 gene
Yy1	0.969	1.957483	0.0425	1422570_at	YY1 transcription factor
2700099C18	0.976	1.967004	0.0327	1427141_at	NDC80 homolog, kinetochore complex component pseudogene
Kat2b	0.977	1.968368	0.0446	1434037_s_at	K(lysine) acetyltransferase 2B
Rrm1	0.979	1.971099	0.0471	1448127_at	ribonucleotide reductase M1
C1galt1c1	0.982	1.975202	0.00929	1416655_at	C1GALT1-specific chaperone 1
Bcl2l13	0.983	1.976571	0.0178	1424406_at	BCL2-like 13 (apoptosis facilitator)
Mllt4	0.984	1.977942	0.027	1436303_at	myeloid/lymphoid or mixed-lineage leukemia (trithorax homolog, Drosophila); translocated to, 4
Wdr5	0.984	1.977942	0.036	1448389_at	WD repeat domain 5
Pak4	0.985	1.979313	0.0444	1428548_at	p21 protein (Cdc42/Rac)-activated kinase 4
Gtpbp2	0.986	1.980686	0.0317	1416690_at	GTP binding protein 2
Rod1	0.987	1.982059	0.0284	1424083_at	ROD1 regulator of differentiation 1 (S. pombe)
Gm608	0.992	1.98894	0.0189	1428681_at	predicted gene 608
Wbp5	0.993	1.990319	0.0155	1451230_a_at	WW domain binding protein 5
E2f1	0.999	1.998614	0.0149	1431875_a_at	E2F transcription factor 1
Igsf8	0.999	1.998614	0.0228	1460675_at	immunoglobulin superfamily, member 8
Mcl1	1.002	2.002775	0.0116	1416881_at	myeloid cell leukemia sequence 1
LOC1000486	1.005	2.006943	0.0422	1459885_s_at	cytochrome c oxidase, subunit VIIc /// cytochrome c oxidase subunit 7C, mitochondrial-like
Commd1	1.009	2.012516	0.0247	1420852_a_at	UDP-GlcNAc:betaGal beta-1,3-N-acetylglucosaminyltransferase 2 /// COMM domain containing 1
Ube2a	1.01	2.013911	0.0117	1417609_at	ubiquitin-conjugating enzyme E2A
Map2k7	1.012	2.016705	0.0088	1425513_at	mitogen-activated protein kinase kinase 7
Ints12	1.012	2.016705	0.0459	1428985_at	integrator complex subunit 12
Rph3al	1.014	2.019503	0.0317	1444409_at	rabphilin 3A-like (without C2 domains)
Poc5	1.016	2.022304	0.0455	1419265_at	POC5 centriolar protein homolog (Chlamydomonas)
Arhgef11	1.016	2.022304	0.0488	1434926_at	Rho guanine nucleotide exchange factor (GEF) 11
Kntc1	1.019	2.026514	0.0178	1435575_at	kinetochore associated 1
Cep152	1.019	2.026514	0.0437	1427496_at	centrosomal protein 152
Pdpk1	1.02	2.027919	0.0164	1416501_at	3-phosphoinositide dependent protein kinase 1
Ncoa3	1.02	2.027919	0.0209	1422737_at	nuclear receptor coactivator 3
Habp4	1.021	2.029325	0.036	1430869_a_at	hyaluronic acid binding protein 4
Clcc1	1.024	2.033549	0.0149	1437511_x_at	chloride channel CLIC-like 1
Lin52	1.025	2.034959	0.0456	1434161_at	predicted gene 7020 /// lin-52 homolog (C. elegans)
Prpf3	1.026	2.03637	0.0178	1424314_at	PRP3 pre-mRNA processing factor 3 homolog (yeast)
Thoc3	1.027	2.037782	0.0463	1423971_at	THO complex 3
Wdr53	1.028	2.039195	0.00116	1428839_at	WD repeat domain 53
Rbm41	1.031	2.04344	0.0335	1456027_at	RNA binding motif protein 41
Pold1	1.032	2.044857	0.028	1448187_at	polymerase (DNA directed), delta 1, catalytic subunit
Rp9	1.033	2.046275	0.0272	1418277_at	retinitis pigmentosa 9 (human)
Prim2	1.035	2.049114	0.0115	1418036_at	DNA primase, p58 subunit
Nbn	1.035	2.049114	0.0343	1448746_at	nibrin
Rad54b	1.036	2.050534	0.0165	1443318_at	fibrinogen silencer binding protein /// RAD54 homolog B (S. cerevisiae)

Zranb2	1.039	2.054803	0.00195	1454652_at	zinc finger, RAN-binding domain containing 2
Atl2	1.039	2.054803	0.0115	1431197_at	atlastin GTPase 2
Sclt1	1.044	2.061937	0.046	1455608_at	sodium channel and clathrin linker 1
Gm2036	1.048	2.067661	0.0327	1424365_at	RIKEN cDNA 1810037I17 gene /// predicted gene 2036
Wdr76	1.06	2.084932	0.0205	1455674_at	WD repeat domain 76
Gins2	1.062	2.087824	0.0382	1428713_s_at	GINS complex subunit 2 (Psf2 homolog)
Man1b1	1.062	2.087824	0.041	1452235_at	mannosidase, alpha, class 1B, member 1
Hat1	1.064	2.09072	0.0272	1428061_at	histone aminotransferase 1
Adar	1.066	2.093621	0.016	1434268_at	adenosine deaminase, RNA-specific
Brd3	1.066	2.093621	0.0296	1460328_at	bromodomain containing 3
E2f8	1.066	2.093621	0.0367	1444319_at	E2F transcription factor 8
Pik3cd	1.069	2.097979	0.016	1422992_s_at	phosphatidylinositol 3-kinase catalytic delta polypeptide
Kdm5c	1.071	2.100889	0.0189	1426497_at	lysine (K)-specific demethylase 5C
Chd1l	1.071	2.100889	0.0272	1449415_at	chromodomain helicase DNA binding protein 1-like
Ncoa2	1.072	2.102346	0.0463	1450458_at	nuclear receptor coactivator 2
Magef1	1.073	2.103804	0.0397	1429479_at	melanoma antigen family F, 1
Ddx20	1.075	2.106722	0.0353	1416751_a_at	DEAD (Asp-Glu-Ala-Asp) box polypeptide 20
Mcm6	1.076	2.108183	0.0111	1416251_at	minichromosome maintenance deficient 6 (MIS5 homolog, S. pombe) (S. cerevisiae)
Gle1	1.077	2.109645	0.00895	1440720_s_at	GLE1 RNA export mediator (yeast)
1600002H07	1.08	2.114036	0.0175	1428832_at	RIKEN cDNA 1600002H07 gene
Kidins220	1.08	2.114036	0.024	1430449_at	kinase D-interacting substrate 220
Exo1	1.087	2.124318	0.0372	1430225_at	exonuclease 1
Strn	1.088	2.125791	0.0175	1422036_at	striatin, calmodulin binding protein
2310001A20	1.09	2.12874	0.00162	1452159_at	RIKEN cDNA 2310001A20 gene
Peli1	1.091	2.130216	0.0372	1431061_s_at	pellino 1
Zfp292	1.092	2.131693	0.0294	1430216_at	zinc finger protein 292
Chrd	1.093	2.133172	0.024	1417304_at	chordin
Usp12	1.097	2.139094	0.046	1425805_a_at	ubiquitin specific peptidase 12
Setx	1.107	2.153973	0.000333	1458080_at	senataxin
Lin7c	1.11	2.158456	0.0203	1449262_s_at	lin-7 homolog C (C. elegans)
Cep76	1.111	2.159953	0.0306	1437254_at	centrosomal protein 76
Mis18bp1	1.116	2.167452	0.0162	1458374_at	MIS18 binding protein 1
Cdc42se1	1.116	2.167452	0.0409	1428131_a_at	CDC42 small effector 1
Cbl	1.118	2.170459	0.0402	1450457_at	Casitas B-lineage lymphoma
Nusap1	1.119	2.171964	0.026	1416309_at	nucleolar and spindle associated protein 1
Sap25	1.12	2.17347	0.00813	1424879_at	predicted gene 20605 /// leucine-rich repeats and calponin homology (CH) domain containing 4 /// sin3 associated polypeptide
Taf3	1.121	2.174977	0.0253	1440187_at	TAF3 RNA polymerase II, TATA box binding protein (TBP)-associated factor
Daxx	1.125	2.181015	0.00141	1419026_at	Fas death domain-associated protein
Wdtc1	1.126	2.182528	0.0104	1434560_at	WD and tetratricopeptide repeats 1
Mknk2	1.126	2.182528	0.0363	1449029_at	MAP kinase-interacting serine/threonine kinase 2
Zfp598	1.137	2.199232	0.0204	1460691_at	zinc finger protein 598
Tubb5	1.139	2.202283	0.0377	1455719_at	tubulin, beta 5 class I
Arfgef1	1.14	2.20381	0.0262	1437104_at	ADP-ribosylation factor guanine nucleotide-exchange factor 1(brefeldin A-inhibited)
2310008H04	1.148	2.216065	0.0237	1451171_at	RIKEN cDNA 2310008H04 gene
Ankrd32	1.153	2.223758	0.00902	1423577_at	ankyrin repeat domain 32
Ing3	1.156	2.228387	0.0419	1450760_a_at	inhibitor of growth family, member 3
Tfeb	1.159	2.233026	0.00375	1422566_at	transcription factor EB
Tbc1d25	1.16	2.234574	0.0115	1456113_at	TBC1 domain family, member 25
Ncaph2	1.166	2.243887	0.0197	1423699_at	non-SMC condensin II complex, subunit H2
Yipf6	1.166	2.243887	0.0476	1455111_at	Yip1 domain family, member 6
Ppp1r13l	1.168	2.247	0.0475	1439530_a_at	protein phosphatase 1, regulatory (inhibitor) subunit 13 like
Mphosph9	1.171	2.251677	0.0293	1431053_at	M-phase phosphoprotein 9
Zmynd19	1.173	2.254801	0.0278	1419522_at	zinc finger, MYND domain containing 19
Lgmn	1.173	2.254801	0.0484	1448883_at	legumain
Slc25a25	1.176	2.259494	0.014	1424735_at	solute carrier family 25 (mitochondrial carrier, phosphate carrier), member 25
Prkci	1.177	2.261061	0.00141	1448695_at	protein kinase C, iota
Tcerg1	1.179	2.264198	0.0317	1450100_a_at	transcription elongation regulator 1 (CA150)
Hmgb2	1.18	2.265768	0.024	1452534_a_at	high mobility group box 2
Ect2	1.181	2.267339	0.0344	1419513_a_at	ect2 oncogene
Ccdc77	1.184	2.272059	0.00964	1425734_a_at	coiled-coil domain containing 77

Ncaph	1.187	2.276788	0.0133	1423920_at	non-SMC condensin I complex, subunit H
Prune	1.188	2.278367	0.0178	1434138_at	prune homolog (Drosophila)
Iqgap3	1.191	2.283109	0.0195	1434850_at	IQ motif containing GTPase activating protein 3
Rttn	1.191	2.283109	0.0437	1436588_at	rotatin
Mcm7	1.194	2.287862	0.0197	1416031_s_at	minichromosome maintenance deficient 7 (S. cerevisiae)
Taf4a	1.198	2.294214	0.00592	1452438_s_at	TAF4A RNA polymerase II, TATA box binding protein (TBP)-associated factor
Odf2l	1.204	2.303775	0.0185	1431861_a_at	outer dense fiber of sperm tails 2-like
Gpsm2	1.204	2.303775	0.0291	1424895_at	G-protein signalling modulator 2 (AGS3-like, C. elegans)
E130303B06	1.205	2.305373	0.013	1434981_at	RIKEN cDNA E130303B06 gene
Haus5	1.205	2.305373	0.0272	1452715_at	HAUS augmin-like complex, subunit 5
Ero1lb	1.207	2.308571	0.0484	1425705_a_at	ERO1-like beta (S. cerevisiae)
Ska1	1.208	2.310172	0.0115	1450496_a_at	spindle and kinetochore associated complex subunit 1
Srgap2	1.208	2.310172	0.0273	1434407_at	SLIT-ROBO Rho GTPase activating protein 2
2700049A03	1.209	2.311773	0.0071	1437248_at	RIKEN cDNA 2700049A03 gene
Samhd1	1.212	2.316586	0.0188	1434438_at	SAM domain and HD domain, 1
Ttll7	1.212	2.316586	0.0296	1430615_at	tubulin tyrosine ligase-like family, member 7
Cep350	1.214	2.319799	0.0182	1431102_at	centrosomal protein 350
Xpr1	1.215	2.321408	0.0421	1426053_a_at	xenotropic and polytropic retrovirus receptor 1
2610021K21	1.215	2.321408	0.0465	1431667_s_at	RIKEN cDNA 2610021K21 gene
Pole2	1.219	2.327853	0.0484	1427094_at	polymerase (DNA directed), epsilon 2 (p59 subunit)
Casp2	1.223	2.334316	0.00265	1448165_at	caspase 2
Elf4	1.228	2.34242	0.00375	1421337_at	E74-like factor 4 (ets domain transcription factor)
Foxp4	1.229	2.344045	0.0272	1431088_at	forkhead box P4
A830080D01	1.23	2.34567	0.0294	1434910_at	RIKEN cDNA A830080D01 gene
Zc3h15	1.231	2.347296	0.00075	1446512_at	zinc finger CCCH-type containing 15
Dgkd	1.234	2.352183	0.0456	1454655_at	diacylglycerol kinase, delta
Crtc2	1.235	2.353813	0.00585	1429373_x_at	CREB regulated transcription coactivator 2
Dck	1.238	2.358713	0.03	1428838_a_at	deoxycytidine kinase
Snx16	1.239	2.360349	0.0165	1438803_s_at	sorting nexin 16
Ap1ar	1.244	2.368543	0.00138	1426234_s_at	adaptor-related protein complex 1 associated regulatory protein
Rbbp8	1.248	2.375119	0.0155	1427062_at	retinoblastoma binding protein 8
Zdhhc18	1.256	2.388326	0.0464	1455518_at	zinc finger, DHHC domain containing 18
Usp7	1.257	2.389982	0.00611	1437118_at	ubiquitin specific peptidase 7
Rad51ap1	1.257	2.389982	0.0157	1417939_at	RAD51 associated protein 1
Timeless	1.257	2.389982	0.0462	1417586_at	timeless homolog (Drosophila)
Pkp4	1.259	2.393298	0.0199	1452209_at	plakophilin 4
Nras	1.263	2.399943	0.00138	1454060_a_at	neuroblastoma ras oncogene
Plk4	1.269	2.409945	0.0201	1419838_s_at	polo-like kinase 4
Pip5k1a	1.271	2.413288	0.0216	1426009_a_at	phosphatidylinositol-4-phosphate 5-kinase, type 1 alpha
Phka2	1.275	2.419988	0.0155	1434518_at	phosphorylase kinase alpha 2
Lmtk2	1.278	2.425026	0.00849	1455018_at	lemur tyrosine kinase 2
G2e3	1.285	2.436821	0.0451	1434700_at	G2/M-phase specific E3 ubiquitin ligase
Mcm4	1.286	2.43851	0.00168	1416214_at	minichromosome maintenance deficient 4 homolog (S. cerevisiae)
Arhgap5	1.286	2.43851	0.0335	1423194_at	Rho GTPase activating protein 5
Lpcat4	1.294	2.45207	0.0268	1440207_at	lysophosphatidylcholine acyltransferase 4
Fbxo45	1.296	2.455471	0.0197	1428742_at	F-box protein 45
Sgms2	1.296	2.455471	0.0262	1428663_at	sphingomyelin synthase 2
Hdac6	1.296	2.455471	0.0278	1448928_at	histone deacetylase 6
Trpm7	1.298	2.458878	0.038	1416801_at	transient receptor potential cation channel, subfamily M, member 7
Larp7	1.299	2.460583	0.0164	1429558_a_at	La ribonucleoprotein domain family, member 7
2610039C10	1.3	2.462289	0.00198	1453314_x_at	RIKEN cDNA 2610039C10 gene
Nup205	1.301	2.463996	0.024	1430343_at	nucleoporin 205
Rp2h	1.304	2.469125	0.0311	1419585_at	retinitis pigmentosa 2 homolog (human)
Numb	1.304	2.469125	0.0487	1425368_a_at	numb gene homolog (Drosophila)
Pebp1	1.31	2.479415	0.028	1453107_s_at	RIKEN cDNA 4933413G19 gene /// forkhead box M1 /// phosphatidylethanolamine binding protein 1
Cdc45	1.312	2.482855	0.0245	1416575_at	cell division cycle 45
Rfc4	1.314	2.486299	0.00745	1424321_at	replication factor C (activator 1) 4
Dclre1b	1.315	2.488023	0.0178	1440383_at	DNA cross-link repair 1B, PSO2 homolog (S. cerevisiae)
Homer1	1.32	2.496661	0.0225	1439662_at	homer homolog 1 (Drosophila)
BC055324	1.321	2.498392	0.028	1437186_at	cDNA sequence BC055324

Pkmyt1	1.331	2.51577	0.013	1418481_at	protein kinase, membrane associated tyrosine/threonine 1
Rfxap	1.334	2.521007	0.0386	1418859_at	regulatory factor X-associated protein
Fam107b	1.337	2.526255	0.0435	1416893_at	family with sequence similarity 107, member B
Aqr	1.343	2.536783	0.00116	1430971_a_at	aquarius
Enc1	1.347	2.543826	0.00929	1420964_at	ectodermal-neural cortex 1
Dgcr14	1.349	2.547355	0.00374	1416822_at	DiGeorge syndrome critical region gene 14
Ppip5k1	1.354	2.556199	0.0273	1460584_at	diphosphoinositol pentakisphosphate kinase 1
Nkap	1.366	2.577549	0.026	1419763_at	NFKB activating protein
Chaf1b	1.368	2.581125	0.00727	1423877_at	chromatin assembly factor 1, subunit B (p60)
Depdc1a	1.368	2.581125	0.0447	1424292_at	DEP domain containing 1a
C2cd2	1.371	2.586498	0.0293	1437731_at	C2 calcium-dependent domain containing 2
Ammecr1	1.373	2.590086	0.0329	1450546_at	Alport syndrome, mental retardation, midface hypoplasia and elliptocytosis chromosomal region gene 1
Cpox	1.376	2.595478	0.00624	1422492_at	coproporphyrinogen oxidase
4930422G04	1.378	2.599078	0.0227	1453038_at	RIKEN cDNA 4930422G04 gene
Zfp518b	1.378	2.599078	0.0228	1441134_at	zinc finger protein 518B
Mms22l	1.379	2.60088	0.013	1427147_at	MMS22-like, DNA repair protein
Ccdc82	1.379	2.60088	0.0155	1421383_at	coiled-coil domain containing 82
Cep110	1.38	2.602684	0.00141	1421004_at	centrosomal protein 110
Chtf18	1.382	2.606294	0.0273	1452098_at	CTF18, chromosome transmission fidelity factor 18
Lig1	1.385	2.61172	0.00585	1416641_at	ligase I, DNA, ATP-dependent
Setdb2	1.387	2.615343	0.00328	1436120_at	SET domain, bifurcated 2
Zfp367	1.387	2.615343	0.0344	1438772_at	zinc finger protein 367
Elf2	1.388	2.617156	0.00784	1428045_a_at	E74-like factor 2
A030009H04	1.39	2.620787	0.0278	1448952_at	RIKEN cDNA A030009H04 gene
Raver1	1.393	2.626242	0.0343	1427110_at	ribonucleoprotein, PTB-binding 1
Pde7a	1.398	2.63536	0.016	1451839_a_at	phosphodiesterase 7A
Lfng	1.402	2.642677	7.26E-05	1449943_at	LFNG O-fucosylpeptide 3-beta-N-acetylglucosaminyltransferase
Tcf19	1.405	2.648178	0.0444	1423809_at	transcription factor 19
Mcm2	1.407	2.651852	0.00195	1448777_at	minichromosome maintenance deficient 2 mitotin (S. cerevisiae)
Pde4dip	1.408	2.65369	0.0476	1460426_at	phosphodiesterase 4D interacting protein (myomegalin)
Cdk16	1.417	2.670297	0.0278	1460169_a_at	cyclin-dependent kinase 16
Cdc25b	1.419	2.674001	0.0273	1421963_a_at	cell division cycle 25B
Eif4g1	1.421	2.677711	0.0395	1438686_at	eukaryotic translation initiation factor 4, gamma 1
Rell1	1.422	2.679567	0.00138	1427243_at	RELT-like 1
Kifc1	1.424	2.683284	0.0477	1449877_s_at	kinesin family member C1
Hdgf	1.435	2.703822	0.00819	1415888_at	hepatoma-derived growth factor
Nktr	1.437	2.707573	0.00141	1423249_at	natural killer tumor recognition sequence
Cdt1	1.437	2.707573	0.0463	1424144_at	chromatin licensing and DNA replication factor 1
Fanci	1.44	2.713209	0.00495	1425941_a_at	Fanconi anemia, complementation group I
Slc25a15	1.445	2.722628	0.0262	1420966_at	solute carrier family 25 (mitochondrial carrier ornithine transporter), member 15
Mtmr1	1.446	2.724516	0.0332	1421879_at	myotubularin related protein 1
Senp2	1.449	2.730187	0.00871	1455327_at	SUMO/sentrin specific peptidase 2
Sgol1	1.451	2.733975	0.0203	1439510_at	shugoshin-like 1 (S. pombe)
4930534B04	1.458	2.747272	0.0344	1430815_at	RIKEN cDNA 4930534B04 gene
Dna2	1.461	2.752991	0.00784	1452210_at	DNA replication helicase 2 homolog (yeast)
Pld1	1.468	2.766381	0.00138	1437112_at	phospholipase D1
BC046331	1.469	2.768299	0.0156	1435156_at	cDNA sequence BC046331
Pnn	1.486	2.801113	0.0104	1423325_at	pinin
Pla2g12a	1.487	2.803055	0.0157	1452026_a_at	phospholipase A2, group XIIA
Pola1	1.497	2.822552	0.0463	1419397_at	polymerase (DNA directed), alpha 1
Rlf	1.502	2.832351	0.0303	1439555_at	rearranged L-myc fusion sequence
Lmnb1	1.505	2.838247	0.0189	1423521_at	lamin B1
Casc5	1.506	2.840215	0.00599	1438833_at	cancer susceptibility candidate 5
1110007C09	1.509	2.846127	0.0357	1431359_a_at	RIKEN cDNA 1110007C09 gene
Msh3	1.521	2.869899	0.00671	1422261_a_at	mutS homolog 3 (E. coli)
Smc4	1.522	2.871889	0.0162	1427276_at	structural maintenance of chromosomes 4
Terf1	1.534	2.895876	0.0328	1431332_a_at	telomeric repeat binding factor 1
Smpd4	1.54	2.907945	0.0146	1437024_at	sphingomyelin phosphodiesterase 4
Ppp1r15b	1.545	2.918041	0.0149	1436366_at	protein phosphatase 1, regulatory (inhibitor) subunit 15b
Abhd10	1.548	2.924115	0.0455	1427958_at	abhydrolase domain containing 10

Ptar1	1.552	2.932234	0.0357	1432162_s_at	protein prenyltransferase alpha subunit repeat containing 1
D330028D13	1.562	2.952629	0.0247	1434428_at	RIKEN cDNA D330028D13 gene
Usp49	1.565	2.958775	0.00301	1453037_at	ubiquitin specific peptidase 49
Rbl1	1.569	2.96699	0.00116	1424156_at	retinoblastoma-like 1 (p107)
Ccdc21	1.577	2.983488	0.0448	1451212_at	coiled-coil domain containing 21
Paqr4	1.578	2.985557	0.0485	1423101_at	progestin and adipoQ receptor family member IV
Glis2	1.585	3.000078	0.00245	1416599_at	GLIS family zinc finger 2
Tube1	1.587	3.00424	0.00964	1431873_a_at	epsilon-tubulin 1
Donson	1.595	3.020945	0.00116	1426739_at	downstream neighbor of SON
Zfp664	1.601	3.033535	0.0237	1437047_at	zinc finger protein 664
Mybl2	1.601	3.033535	0.0332	1454946_at	myeloblastosis oncogene-like 2
Fanca	1.605	3.041958	0.00853	1452513_a_at	Fanconi anemia, complementation group A
Nfkbiz	1.606	3.044067	0.0492	1417483_at	nuclear factor of kappa light polypeptide gene enhancer in B cells inhibitor, zeta
Rad51c	1.608	3.04829	0.014	1423026_at	RAD51 homolog c (S. cerevisiae)
Baz1b	1.61	3.052518	0.0255	1450068_at	bromodomain adjacent to zinc finger domain, 1B
Zfyve20	1.614	3.060994	0.0195	1429517_at	zinc finger, FYVE domain containing 20
Eif5a2	1.623	3.080149	0.0352	1434593_at	eukaryotic translation initiation factor 5A2
Hjurp	1.626	3.08656	0.00142	1442202_at	Holliday junction recognition protein
Anp32a	1.632	3.099424	0.0195	1450407_a_at	acidic (leucine-rich) nuclear phosphoprotein 32 family, member A
Arid5a	1.632	3.099424	0.03	1451340_at	AT rich interactive domain 5A (MRF1-like)
Tonsl	1.635	3.105876	0.00614	1428661_at	tonsoku-like, DNA repair protein
Gltp	1.646	3.129647	0.0163	1419027_s_at	glycolipid transfer protein
Arl5b	1.652	3.14269	0.0339	1437884_at	ADP-ribosylation factor-like 5B
Ncapd3	1.653	3.144869	0.000657	1430782_at	non-SMC condensin II complex, subunit D3
Cenpf	1.653	3.144869	0.00579	1458447_at	centromere protein F
Kctd6	1.654	3.14705	0.0123	1428186_at	potassium channel tetramerisation domain containing 6
Piga	1.654	3.14705	0.0289	1453497_a_at	phosphatidylinositol glycan anchor biosynthesis, class A
Intu	1.663	3.166743	0.0202	1458595_at	inturned planar cell polarity effector homolog (Drosophila)
Cyp39a1	1.673	3.18877	0.0421	1418780_at	cytochrome P450, family 39, subfamily a, polypeptide 1
2010317E24	1.686	3.217633	0.00521	1429404_at	RIKEN cDNA 2010317E24 gene
Elp4	1.686	3.217633	0.0162	1416987_at	elongation protein 4 homolog (S. cerevisiae)
Arhgap12	1.698	3.244509	0.0497	1431052_at	Rho GTPase activating protein 12
Trim59	1.708	3.267076	0.0202	1431430_s_at	tripartite motif-containing 59
Prkx	1.712	3.276147	0.0264	1424287_at	protein kinase, X-linked
Bcl3	1.713	3.278418	0.00495	1418133_at	B cell leukemia/lymphoma 3
Mllt1	1.727	3.310387	0.0317	1421060_at	myeloid/lymphoid or mixed-lineage leukemia (trithorax homolog, Drosophila); translocated to, 1
Arnt	1.745	3.351949	0.0148	1421721_a_at	aryl hydrocarbon receptor nuclear translocator
Wdr47	1.747	3.356599	9.24E-07	1437257_at	WD repeat domain 47
Ptpn21	1.752	3.368252	0.0385	1419054_a_at	protein tyrosine phosphatase, non-receptor type 21
Dusp8	1.757	3.379946	0.000798	1428864_at	dual specificity phosphatase 8
Comt	1.758	3.382289	0.00141	1418701_at	catechol-O-methyltransferase
Foxm1	1.762	3.39168	0.00683	1448833_at	forkhead box M1
Parp12	1.794	3.46775	0.0354	1426774_at	poly (ADP-ribose) polymerase family, member 12
Xiap	1.813	3.513722	0.00138	1421394_a_at	X-linked inhibitor of apoptosis
Rif1	1.815	3.518596	0.000647	1442939_at	Rap1 interacting factor 1 homolog (yeast)
Mcm3	1.819	3.528365	0.0173	1426652_at	minichromosome maintenance deficient 3 (S. cerevisiae)
Tnfaip8l1	1.829	3.552907	0.0344	1449125_at	tumor necrosis factor, alpha-induced protein 8-like 1
Setdb1	1.833	3.562772	0.00585	1416670_at	SET domain, bifurcated 1
Slc16a4	1.847	3.597513	0.0296	1456914_at	Solute carrier family 16 (monocarboxylic acid transporters), member 4
2900054C01	1.848	3.600008	0.0249	1429625_at	RIKEN cDNA 2900054C01 gene
Ercc6l	1.882	3.685857	0.0133	1444024_at	excision repair cross-complementing rodent repair deficiency complementation group 6 - like
Zfx	1.901	3.73472	0.0227	1449512_a_at	zinc finger protein X-linked
Celf4	1.924	3.794737	0.0319	1426930_at	CUGBP, Elav-like family member 4
Syt12	1.928	3.805273	0.0183	1422878_at	synaptotagmin XII
Nup188	1.932	3.815838	0.0116	1442314_at	nucleoporin 188
Dusp6	1.938	3.831741	0.0211	1415834_at	dual specificity phosphatase 6
Pfkp	1.967	3.909543	0.00603	1430634_a_at	phosphofructokinase, platelet
Rbmx2	1.967	3.909543	0.0189	1424705_at	RNA binding motif protein, X-linked 2
Cbx7	1.967	3.909543	0.0203	1433557_at	chromobox 7
Smc1a	1.99	3.97237	0.00636	1417832_at	structural maintenance of chromosomes 1A

Mpp7	1.991	3.975124	0.00375	1432216_s_at	membrane protein, palmitoylated 7 (MAGUK p55 subfamily member 7)
Nrm	2	4	0.00593	1417971_at	nurim (nuclear envelope membrane protein)
Tnfaip3	2.012	4.03341	0.0285	1450829_at	tumor necrosis factor, alpha-induced protein 3
Rprd1a	2.019	4.053028	0.0228	1438065_at	regulation of nuclear pre-mRNA domain containing 1A
Ccdc120	2.026	4.072741	0.0197	1428066_at	coiled-coil domain containing 120
Arrb2	2.029	4.081219	0.00195	1451987_at	arrestin, beta 2
BC006779	2.032	4.089714	0.0273	1435454_a_at	cDNA sequence BC006779
Rem1	2.09	4.257481	0.0403	1450158_at	rad and gem related GTP binding protein 1
Atp11c	2.102	4.293041	0.0196	1442367_at	ATPase, class VI, type 11C
Taf1	2.132	4.383247	0.00473	1447417_at	TAF1 RNA polymerase II, TATA box binding protein (TBP)-associated factor
Rps6ka3	2.171	4.503354	0.00261	1452383_at	ribosomal protein S6 kinase polypeptide 3
Mir1199	2.196	4.582072	0.0228	1440194_at	microRNA 1199
Ptprk	2.264	4.803214	0.0273	1431680_a_at	protein tyrosine phosphatase, receptor type, K
Bhlhe40	2.281	4.860147	0.0078	1418025_at	basic helix-loop-helix family, member e40
Herc6	2.29	4.890561	0.04	1432026_a_at	hect domain and RLD 6
Rpia	2.299	4.921165	0.0311	1418337_at	ribose 5-phosphate isomerase A
Clic5	2.342	5.07005	0.0385	1439505_at	chloride intracellular channel 5
Sema4a	2.377	5.194554	0.00871	1448110_at	sema domain, immunoglobulin domain (Ig), transmembrane domain (TM) and short cytoplasmic domain, (semaphorin) 4A
Rbm38	2.455	5.483131	0.024	1421265_a_at	RNA binding motif protein 38
Gabpb2	2.459	5.498355	9.24E-07	1440868_at	GA repeat binding protein, beta 2
Stat1	2.474	5.555821	0.0449	1450033_a_at	signal transducer and activator of transcription 1
Tspan15	2.517	5.723906	0.00585	1424653_at	tetraspanin 15
BB182297	2.551	5.860403	0.00495	1436585_at	expressed sequence BB182297
Agrn	2.559	5.892991	0.048	1426670_at	agrin
Dusp5	2.636	6.216058	0.0228	1437199_at	dual specificity phosphatase 5
Aspm	2.67	6.364292	0.00138	1441520_at	asp (abnormal spindle)-like, microcephaly associated (Drosophila)
Slfn9	2.702	6.507034	0.014	1436472_at	schlafen 9
Syce2	2.827	7.09597	0.00863	1429270_a_at	synaptonemal complex central element protein 2
Irf9	2.944	7.69542	0.00682	1421322_a_at	interferon regulatory factor 9
Gch1	3.026	8.145482	0.0209	1420499_at	GTP cyclohydrolase 1
Igf2bp1	3.066	8.374482	0.0175	1418761_at	insulin-like growth factor 2 mRNA binding protein 1
Gcnt2	3.085	8.485502	0.0149	1430826_s_at	glucosaminyl (N-acetyl) transferase 2, I-branching enzyme
Atf3	3.141	8.821353	0.0343	1449363_at	activating transcription factor 3
Bst2	3.288	9.767572	0.0459	1424921_at	bone marrow stromal cell antigen 2
Fgf18	3.34	10.12605	0.0216	1449545_at	fibroblast growth factor 18
Ccl5	3.399	10.54875	0.0372	1418126_at	chemokine (C-C motif) ligand 5
Ano1	3.411	10.63686	0.0493	1459713_s_at	anoctamin 1, calcium activated chloride channel
5730508B09	3.431	10.78534	0.000798	1429678_at	RIKEN cDNA 5730508B09 gene
Gpr64	3.536	11.59957	0.0355	1438350_at	G protein-coupled receptor 64
Gm20559	3.591	12.05032	0.0392	1436172_at	predicted gene, 20559
2210011C24	3.801	13.93847	0.0184	1429953_at	RIKEN cDNA 2210011C24 gene
Ucp2	3.837	14.29065	0.0182	1448188_at	uncoupling protein 2 (mitochondrial, proton carrier)
Cxcl10	3.962	15.58407	0.00141	1418930_at	chemokine (C-X-C motif) ligand 10
Oasl2	4.074	16.8421	0.0216	1453196_a_at	2'-5' oligoadenylate synthetase-like 2
Neurl1b	4.134	17.55731	0.00075	1435564_at	neuralized homolog 1b (Drosophila)
Isg15	4.3	19.69831	0.026	1431591_s_at	predicted gene 9706 /// ISG15 ubiquitin-like modifier
Oasl1	4.452	21.88696	0.0119	1424339_at	2'-5' oligoadenylate synthetase-like 1
Rsad2	4.514	22.84806	0.0493	1436058_at	radical S-adenosyl methionine domain containing 2
Oas1a	4.531	23.11889	0.0368	1424775_at	2'-5' oligoadenylate synthetase 1A
Rtp4	4.648	25.07191	0.0413	1418580_at	receptor transporter protein 4
Ifit1	4.901	29.87776	0.0148	1450783_at	interferon-induced protein with tetratricopeptide repeats 1
Cdkn2a	5.324	40.05749	0.00189	1450140_a_at	cyclin-dependent kinase inhibitor 2A
Ifi27l2a	5.508	45.50648	0.0289	1426278_at	interferon, alpha-inducible protein 27 like 2A
Usp18	5.762	54.26688	0.0215	1418191_at	ubiquitin specific peptidase 18
Cdkn2b	6.321	79.94855	0.0367	1449152_at	cyclin-dependent kinase inhibitor 2B (p15, inhibits CDK4)
