# Supplementary material for: Consistent gene expression profiles in MexTAg transgenic mouse and wild type mouse asbestos-induced mesothelioma
Source: BMC Cancer. 2015 Dec 18;15:983. doi: 10.1186/s12885-015-1953-y (PMC4683914; doi:10.1186/s12885-015-1953-y)
Supplement: Additional file 3: Table S3. — Genes significantly differentially expressed in hiTAgtu and WTtu sample sets compared to wild type normal mesothelial cells. (PDF 61 kb) [file 12885_2015_1953_MOESM3_ESM.pdf]

Gene ID	Gene description	WTtu v WTn		hiTAgtu v WTn	
		logFC	adj_p_val	logFC	adj_p_val
Acsl3	acyl-CoA synthetase long-chain family member 3	3.186	0.000407	3.022	0.00053
Mki67	antigen identified by monoclonal antibody Ki 67	3.274	1.52E-05	3.662	2.72E-06
Asf1b	ASF1 anti-silencing function 1 homolog B (S. cerevisiae)	3.111	0.0031	4.632	3.86E-05
Aspm	asp (abnormal spindle)-like, microcephaly associated (Drosophila)	4.154	3.90E-08	4.895	3.25E-09
Atad5	ATPase family, AAA domain containing 5	3.581	1.68E-06	4.301	1.13E-07
Aurka	aurora kinase A	3.117	0.00045	3.881	3.02E-05
Aurkb	aurora kinase B	3.976	6.75E-07	4.58	8.13E-08
Birc5	baculoviral IAP repeat-containing 5	3.678	4.13E-06	3.624	3.28E-06
Blm	Bloom syndrome, RecQ helicase-like	3.118	0.000232	4.102	7.35E-06
Brca1	breast cancer 1	3.435	3.13E-06	4.192	1.71E-07
Bub1	budding uninhibited by benzimidazoles 1 homolog (S. cerevisiae)	3.686	2.21E-06	4.481	1.28E-07
Bub1b	budding uninhibited by benzimidazoles 1 homolog, beta (S. cerevisiae)	3.856	3.73E-07	4.399	5.31E-08
BC055324	cDNA sequence BC055324	3.65	1.38E-05	4.971	2.10E-07
Cdc25c	cell division cycle 25C	4.198	8.48E-08	3.893	9.06E-06
Cdc45	cell division cycle 45	3.808	4.99E-06	5.119	8.02E-08
Cdc6	cell division cycle 6	4.181	2.59E-06	5.031	1.71E-07
Cdca2	cell division cycle associated 2	4.28	0.000223	4.48	9.92E-05
Cdca3	cell division cycle associated 3	3.556	5.99E-06	3.816	1.72E-06
Cdca5	cell division cycle associated 5	5.158	8.92E-08	5.128	7.67E-08
Cdca7	cell division cycle associated 7	4.073	6.19E-05	5.249	2.10E-06
Cdca7l	cell division cycle associated 7 like	3.421	8.18E-06	3.877	1.23E-06
Cdca8	cell division cycle associated 8	3.23	0.00028	4.034	1.68E-05
Cenpa	centromere protein A	3.084	0.000168	3.686	1.61E-05
Cenpe	centromere protein E	3.378	0.000106	4.118	7.70E-06
Cenpf	centromere protein F	4.341	5.34E-06	5.158	4.17E-07
Cenph	centromere protein H	3.156	5.56E-06	3.798	3.75E-07
Cenpi	centromere protein I	4.038	0.000274	5.511	5.83E-06
Cenpl	centromere protein L	3.075	4.62E-05	3.303	1.40E-05
Cenpm	centromere protein M	3.374	0.0005	3.607	0.000189
Cenpn	centromere protein N	3.866	5.49E-06	4.655	3.64E-07
Cenpp	centromere protein P	3.947	3.73E-07	4.444	6.12E-08
Cenpw	centromere protein W	3.09	0.00724	3.937	0.000652
Cep55	centrosomal protein 55	3.77	4.31E-07	4.434	4.11E-08
Chek1	checkpoint kinase 1	3.508	0.000195	4.475	8.83E-06
Cdt1	chromatin licensing and DNA replication factor 1	3.219	4.62E-05	4.085	1.84E-06
Cit	citron	3.108	0.000324	4.214	7.50E-06
Clspn	claspin homolog (Xenopus laevis)	4.293	8.26E-06	4.736	1.71E-06
Chtf18	CTF18, chromosome transmission fidelity factor 18	3.265	7.56E-05	4.647	7.69E-07

Ccna2	cyclin A2	3.816	2.63E-06	4.674	1.39E-07
Gm5593	cyclin B1 /// predicted gene 5593	3.363	5.05E-06	4.224	1.98E-07
Ccnb2	cyclin B2	3.141	2.99E-05	3.504	5.78E-06
Ccne2	cyclin E2	3.049	7.55E-06	3.886	2.50E-07
Ccnf	cyclin F	3.535	0.000909	4.189	0.000118
Dscc1	defective in sister chromatid cohesion 1 homolog (S. cerevisiae)	4.579	2.56E-06	5.846	7.83E-08
Dtl	denticleless homolog (Drosophila)	3.414	7.69E-05	4.591	1.59E-06
Dnmt1	DNA methyltransferase (cytosine-5) 1	3.667	2.02E-05	4.45	1.34E-06
Prim1	DNA primase, p49 subunit	3.678	1.45E-07	4.644	4.97E-09
D17H6S56E-5	DNA segment, Chr 17, human D6S56E 5	3.173	0.000555	3.874	4.75E-05
D2Ertd750e	DNA segment, Chr 2, ERATO Doi 750, expressed	3.295	8.26E-06	3.952	6.10E-07
E2f7	E2F transcription factor 7	4.257	9.90E-06	5.298	4.53E-07
E2f8	E2F transcription factor 8	3.278	0.000432	4.665	5.96E-06
Eme1	essential meiotic endonuclease 1 homolog 1 (S. pombe)	3.056	0.00815	3.472	0.00218
Esco2	establishment of cohesion 1 homolog 2 (S. cerevisiae)	4.139	7.62E-05	4.695	1.25E-05
Ercc6l	excision repair cross-complementing rodent repair deficiency complementat	3.258	0.000348	4.287	1.16E-05
Exo1	exonuclease 1	4.183	1.23E-05	4.785	1.64E-06
Espl1	extra spindle poles-like 1 (S. cerevisiae)	3.012	0.000939	4.024	3.16E-05
Fam54a	family with sequence similarity 54, member A	3.041	0.001	3.916	5.23E-05
Fam64a	family with sequence similarity 64, member A	4.488	6.49E-07	4.093	1.51E-06
Fanca	Fanconi anemia, complementation group A	3.037	6.29E-05	4.002	1.62E-06
Fbxo5	F-box protein 5	3.555	5.49E-06	3.707	2.25E-06
Fignl1	fidgetin-like 1	3.266	8.04E-06	4.248	2.11E-07
Foxm1	forkhead box M1	4.26	1.54E-05	5.767	2.50E-07
Gcat	galanin receptor 3 /// glycine C-acetyltransferase (2-amino-3-ketobutyrate-c	4.272	0.00166	3.652	0.00496
Gsg2	germ cell-specific gene 2	3.822	0.000264	4.901	1.16E-05
Gins1	GINS complex subunit 1 (Psf1 homolog)	3.55	0.00012	4.179	1.34E-05
Glipr1	GLI pathogenesis-related 1 (glioma)	6.474	4.81E-08	5.301	5.04E-07
Gatm	glycine amidinotransferase (L-arginine:glycine amidinotransferase)	3.771	0.0182	4.914	0.00174
Gas2l3	growth arrest-specific 2 like 3	4.798	8.48E-08	5.405	1.38E-08
Hells	helicase, lymphoid specific	4.127	4.52E-07	5.097	2.33E-08
Hmmr	hyaluronan mediated motility receptor (RHAMM)	3.135	2.85E-05	3.62	3.54E-06
Incenp	inner centromere protein	3.224	1.20E-07	3.395	4.99E-08
Iqgap3	IQ motif containing GTPase activating protein 3	3.033	1.34E-05	4.224	1.55E-07
Kif11	kinesin family member 11	4.867	6.74E-08	5.445	1.20E-08
Kif18b	kinesin family member 18B	4.056	2.10E-06	4.843	1.54E-07
Kif20a	kinesin family member 20A	3.382	9.09E-08	3.835	1.38E-08
Kif20b	kinesin family member 20B	3.762	4.38E-07	4.37	4.98E-08
Kif23	kinesin family member 23	5.403	2.40E-07	6.012	4.90E-08
Kif2c	kinesin family member 2C	3.918	5.96E-06	4.319	1.25E-06

Kif4	kinesin family member 4	4.035	9.86E-05	5.204	3.54E-06
Kntc1	kinetochore associated 1	3.163	8.98E-07	4.182	1.83E-08
Ltbp1	latent transforming growth factor beta binding protein 1	4.963	0.00499	3.336	0.0444
Lrr1	leucine rich repeat protein 1	4.09	0.000786	5.333	3.36E-05
Melk	maternal embryonic leucine zipper kinase	3.496	5.14E-05	4.321	2.94E-06
Mastl	microtubule associated serine/threonine kinase-like	3.789	1.49E-05	4.792	5.73E-07
Mcm10	minichromosome maintenance deficient 10 (S. cerevisiae)	4.174	5.88E-05	5.284	2.50E-06
Mcm3	minichromosome maintenance deficient 3 (S. cerevisiae)	3.628	0.000155	5.447	9.09E-07
Mcm5	minichromosome maintenance deficient 5, cell division cycle 46 (S. cerevisia	3.307	5.32E-06	4.094	2.49E-07
Mis18bp1	MIS18 binding protein 1	3.291	3.91E-05	3.505	4.22E-07
Mybl2	myeloblastosis oncogene-like 2	3.42	0.00395	5.001	6.70E-05
Mlf1ip	myeloid leukemia factor 1 interacting protein	3.251	0.000107	3.819	1.21E-05
Ndc80	NDC80 homolog, kinetochore complex component (S. cerevisiae)	3.345	4.67E-06	4.1	2.44E-07
Nrp1	neuropilin 1	4.019	0.00103	3.207	0.00572
Nek2	NIMA (never in mitosis gene a)-related expressed kinase 2	3.36	7.56E-05	4.373	2.35E-06
Ncapd2	non-SMC condensin I complex, subunit D2	3.444	4.33E-06	3.956	5.04E-07
Ncapg	non-SMC condensin I complex, subunit G	3.858	3.14E-06	4.618	2.21E-07
Ncaph	non-SMC condensin I complex, subunit H	3.005	2.76E-05	3.565	4.37E-07
Nusap1	nucleolar and spindle associated protein 1	3.128	8.67E-06	4.247	1.36E-07
Nup43	nucleoporin 43	3.233	2.48E-05	3.315	1.30E-05
Nuf2	NUF2, NDC80 kinetochore complex component, homolog (S. cerevisiae)	3.882	7.10E-06	4.354	1.20E-06
Pbk	PDZ binding kinase	3.479	0.000389	3.964	6.98E-05
Pde4d	phosphodiesterase 4D, cAMP specific	3.173	0.0314	3.533	0.0122
Plk1	polo-like kinase 1	3.362	0.000117	3.649	3.36E-05
Pola1	polymerase (DNA directed), alpha 1	3.538	0.000259	5.035	3.23E-06
Pole	polymerase (DNA directed), epsilon	3.126	4.06E-05	4.06	1.23E-06
Racgap1	Rac GTPase-activating protein 1	3.637	1.03E-05	4.484	5.33E-07
Rad51ap1	RAD51 associated protein 1	4.213	1.17E-07	5.285	4.22E-09
Rad54b	RAD54 homolog B (S. cerevisiae)	3.531	0.000106	5.152	8.43E-07
Rad54l	RAD54 like (S. cerevisiae)	3.613	0.00119	4.193	0.000204
Rfc5	replication factor C (activator 1) 5	3.052	5.59E-06	3.795	2.50E-07
1110012J17Rik	RIKEN cDNA 1110012J17 gene	3.553	0.0157	5.213	0.000457
1200009I06Rik	RIKEN cDNA 1200009I06 gene	3.758	0.0112	3.257	0.0223
2700038G22Rik	RIKEN cDNA 2700038G22 gene	3.875	0.00101	4.155	0.000395
4930534B04Rik	RIKEN cDNA 4930534B04 gene	3.083	0.0363	4.232	0.00298
Pebp1	RIKEN cDNA 4933413G19 gene /// forkhead box M1 /// phosphatidylethano	4.092	3.17E-06	5.401	6.28E-08
5730590G19Rik	RIKEN cDNA 5730590G19 gene	3.076	0.000962	3.858	6.76E-05
A130088B03Rik	RIKEN cDNA A130088B03 gene	3.265	0.000654	3.447	0.000295
C330027C09Rik	RIKEN cDNA C330027C09 gene	3.267	3.45E-05	4.359	7.34E-07
E130306D19Rik	RIKEN cDNA E130306D19 gene	3.078	0.00196	4.408	3.43E-05

F630043A04Rik	RIKEN cDNA F630043A04 gene	3.167	0.000228	3.812	2.05E-05
Sep6	septin 6	3.606	0.0019	3.97	0.000586
Shcbp1	Shc SH2-domain binding protein 1	3.4	3.05E-05	4.015	2.95E-06
Sgol1	shugoshin-like 1 (S. pombe)	4.424	1.73E-06	5.875	3.18E-08
Sgol2	shugoshin-like 2 (S. pombe)	3.523	3.95E-08	4.161	3.25E-09
Slc29a1	solute carrier family 29 (nucleoside transporters), member 1	3.571	0.000957	4.991	1.91E-05
Spc24	SPC24, NDC80 kinetochore complex component, homolog (S. cerevisiae)	3.461	0.0004	3.977	6.61E-05
Spag5	sperm associated antigen 5	4.387	4.14E-08	4.797	1.06E-08
Ska1	spindle and kinetochore associated complex subunit 1	4.333	4.07E-08	5.54	9.89E-10
Sass6	spindle assembly 6 homolog (C. elegans)	3.251	0.000505	4.301	1.68E-05
Suv39h1	suppressor of variegation 3-9 homolog 1 (Drosophila)	3.334	0.00441	4.457	0.000212
Suv39h2	suppressor of variegation 3-9 homolog 2 (Drosophila)	3.237	0.0106	4.483	0.000472
Tiam2	T cell lymphoma invasion and metastasis 2	3.823	0.000342	3.714	0.000351
Tk1	thymidine kinase 1	4.323	4.65E-05	4.201	4.57E-05
Timeless	timeless homolog (Drosophila)	3.784	1.76E-05	5.041	3.49E-07
Top2a	topoisomerase (DNA) II alpha	5.786	2.51E-09	5.15	1.31E-08
Tpx2	TPX2, microtubule-associated protein homolog (Xenopus laevis)	4.127	4.37E-07	4.826	4.54E-08
Tacc3	transforming, acidic coiled-coil containing protein 3	3.651	2.43E-06	4.027	4.74E-07
Troap	trophinin associated protein	3.49	0.00712	4.489	0.000577
Ttk	Ttk protein kinase	4.461	6.49E-07	5.071	9.24E-08
Ube2t	ubiquitin-conjugating enzyme E2T (putative)	3.088	5.78E-06	4.031	1.44E-07
Uhrf1	ubiquitin-like, containing PHD and RING finger domains, 1	4.272	1.43E-07	5.072	1.19E-08
Ung	uracil DNA glycosylase	3.439	0.0395	4.929	0.0027
Wdhd1	WD repeat and HMG-box DNA binding protein 1	3.502	0.000163	4.529	6.18E-06
Zwilch	Zwilch, kinetochore associated, homolog (Drosophila)	3.161	1.09E-05	3.267	5.19E-06
