# Supplementary material for: Consistent gene expression profiles in MexTAg transgenic mouse and wild type mouse asbestos-induced mesothelioma
Source: BMC Cancer. 2015 Dec 18;15:983. doi: 10.1186/s12885-015-1953-y (PMC4683914; doi:10.1186/s12885-015-1953-y)
Supplement: Additional file 4: Table S4. — List of differentially expressed genes in human mesothelioma compared with normal human mesothelial cells. (PDF 307 kb) [file 12885_2015_1953_MOESM4_ESM.pdf]

Gene	logFC	adj_p_val	probeID	genedesc
MSI2	2.869	1.50E-06	243010_at	musashi homolog 2 (Drosophila)
MAGOHB	1.055	3.78E-06	218894_s_at	mago-nashi homolog B (Drosophila)
ARHGAP6	-3.257	1.07E-05	206167_s_at	Rho GTPase activating protein 6
DENND2C	-2.117	3.29E-05	230769_at	DENN/MADD domain containing 2C
TSPAN8	-5.49	0.00119	203824_at	tetraspanin 8
PTGS2	-5.064	0.00119	204748_at	prostaglandin-endoperoxide synthase 2 (prostaglandin G/H synthase and cyclooxygenase)
EGR2	-4.176	0.00119	205249_at	early growth response 2
PKIG	-1.593	0.00119	202732_at	protein kinase (cAMP-dependent, catalytic) inhibitor gamma
C4orf3	-1.221	0.00119	224604_at	chromosome 4 open reading frame 3
CCT7	0.962	0.00119	200812_at	chaperonin containing TCP1, subunit 7 (eta)
RUVBL1	1.51	0.00119	201614_s_at	RuvB-like 1 (E. coli)
SSR1	2.284	0.00119	200890_s_at	signal sequence receptor, alpha
SLC4A11	3.677	0.00119	223748_at	solute carrier family 4, sodium borate transporter, member 11
DGKE	-2.656	0.00153	238694_at	diacylglycerol kinase, epsilon 64kDa
PGM5	-3.057	0.00154	226303_at	phosphoglucomutase 5
DLG1	0.713	0.00154	202515_at	discs, large homolog 1 (Drosophila)
DDX41	0.728	0.00154	217840_at	DEAD (Asp-Glu-Ala-Asp) box polypeptide 41
CDC123	0.99	0.00154	201725_at	cell division cycle 123 homolog (S. cerevisiae)
C20orf112	1.958	0.00154	230954_at	chromosome 20 open reading frame 112
LOC10050588	2.535	0.00154	240382_at	uncharacterized LOC100505880
C20orf160	-2.81	0.00155	231991_at	chromosome 20 open reading frame 160
FANCD2	2.415	0.00166	242560_at	Fanconi anemia, complementation group D2
PPFIA1	1.521	0.0017	210236_at	protein tyrosine phosphatase, receptor type, f polypeptide (PTPRF), interacting protein (liprin), alpha 1
SETDB2	-0.773	0.00172	235339_at	SET domain, bifurcated 2
CALR	1.033	0.00172	200935_at	calreticulin
ADAMTS1	-3.47	0.00176	222162_s_at	ADAM metallopeptidase with thrombospondin type 1 motif, 1
USP53	-1.099	0.00177	231817_at	ubiquitin specific peptidase 53
ANKRD29	-3.497	0.00179	238332_at	ankyrin repeat domain 29
SOCS2	-2.628	0.00179	203373_at	suppressor of cytokine signaling 2
GIPC2	-2.577	0.00179	236548_at	GIPC PDZ domain containing family, member 2
C10orf128	-2.508	0.00179	228372_at	chromosome 10 open reading frame 128
MITF	-2.016	0.00179	226066_at	microphthalmia-associated transcription factor
CYB5R3	-1.092	0.00179	201885_s_at	cytochrome b5 reductase 3
INPP4A	-0.815	0.00179	227087_at	inositol polyphosphate-4-phosphatase, type I, 107kDa
TROVE2	0.467	0.00179	210438_x_at	TROVE domain family, member 2
CEP41	0.744	0.00179	225484_at	centrosomal protein 41kDa
EIF4G3	0.764	0.00179	201936_s_at	eukaryotic translation initiation factor 4 gamma, 3
VGLL4	0.775	0.00179	212399_s_at	vestigial like 4 (Drosophila)
SLC30A5	0.879	0.00179	218989_x_at	solute carrier family 30 (zinc transporter), member 5
COPZ1	0.976	0.00179	217726_at	coatomer protein complex, subunit zeta 1
COX20	1.082	0.00179	224824_at	COX20 Cox2 chaperone homolog (S. cerevisiae)
LONP2	1.108	0.00179	223098_s_at	lon peptidase 2, peroxisomal
RPE	1.18	0.00179	221770_at	rcRPE /// ribulose-5-phosphate-3-epimerase
PPP1R14B	1.365	0.00179	212680_x_at	protein phosphatase 1, regulatory (inhibitor) subunit 14B
STT3A	1.438	0.00179	202223_at	STT3, subunit of the oligosaccharyltransferase complex, homolog A (S. cerevisiae)
SPTLC2	1.623	0.00179	203128_at	serine palmitoyltransferase, long chain base subunit 2
ALDH18A1	1.856	0.00179	217791_s_at	aldehyde dehydrogenase 18 family, member A1
P4HA1	1.998	0.00179	207543_s_at	prolyl 4-hydroxylase, alpha polypeptide I
TMOD3	2.477	0.00179	223078_s_at	tropomodulin 3 (ubiquitous)
CALCRL	-3.233	0.00183	234996_at	calcitonin receptor-like
STARD9	-2.849	0.00183	227108_at	StAR-related lipid transfer (START) domain containing 9
EMCN	-2.696	0.00183	222885_at	endomucin
SPARCL1	-1.87	0.00183	200795_at	SPARC-like 1 (hevin)
SIPA1L2	-1.754	0.00183	233587_s_at	signal-induced proliferation-associated 1 like 2
TACC1	-1.446	0.00183	200911_s_at	transforming, acidic coiled-coil containing protein 1
ASB13	-1.042	0.00183	218862_at	ankyrin repeat and SOCS box containing 13
SKAP2	-0.917	0.00183	225639_at	src kinase associated phosphoprotein 2
SETD4	0.732	0.00183	213989_x_at	SET domain containing 4
NME2	1.125	0.00183	201268_at	NME1-NME2 readthrough /// NME/NM23 nucleoside diphosphate kinase 2
TSPAN2	-3.12	0.00185	227236_at	tetraspanin 2
CSRP2	-2.572	0.00193	207030_s_at	cysteine and glycine-rich protein 2
ARHGAP24	-2.114	0.00193	223422_s_at	Rho GTPase activating protein 24
STOM	-1.737	0.00193	201061_s_at	stomatin
HECA	-1.143	0.00193	218603_at	headcase homolog (Drosophila)
RILPL2	-1.021	0.00193	227983_at	Rab interacting lysosomal protein-like 2
KLHL8	-0.972	0.00193	242648_at	kelch-like 8 (Drosophila)
NUFIP2	0.747	0.00193	224939_at	nuclear fragile X mental retardation protein interacting protein 2
NAA50	1.031	0.00193	222393_s_at	N(alpha)-acetyltransferase 50, NatE catalytic subunit
STAM	1.1	0.00193	203544_s_at	signal transducing adaptor molecule (SH3 domain and ITAM motif) 1
PTS	1.525	0.00202	209694_at	6-pyruvoyltetrahydropterin synthase
STAG3L4	1.877	0.00202	222801_s_at	stromal antigen 3-like 4
LOC10065313	-3.826	0.00202	227404_s_at	uncharacterized LOC100653132
HDAC9	-1.985	0.00206	205659_at	histone deacetylase 9
GNG7	-0.974	0.00206	228831_s_at	guanine nucleotide binding protein (G protein), gamma 7

REV3L	-0.864	0.00206	208070_s_at	REV3-like, polymerase (DNA directed), zeta, catalytic subunit
CANX	1.271	0.00206	208852_s_at	calnexin
ANTXR1	2.503	0.00206	220092_s_at	anthrax toxin receptor 1
LOC10065334	0.938	0.0021	214737_x_at	heterogeneous nuclear ribonucleoprotein C (C1/C2) /// uncharacterized LOC100652761 /// uncharacterized LOC100653343
PDIA6	0.815	0.00211	208638_at	protein disulfide isomerase family A, member 6
MAOA	-4.644	0.00211	212741_at	monoamine oxidase A
RGN	-2.703	0.00211	210751_s_at	regucalcin (senescence marker protein-30)
Mar-02	-2.294	0.00211	221636_s_at	mitochondrial amidoxime reducing component 2
DIP2A	1.123	0.00211	1555301_a_at	DIP2 disco-interacting protein 2 homolog A (Drosophila)
CIAPIN1	1.144	0.00211	208424_s_at	cytokine induced apoptosis inhibitor 1
EIF5	1.257	0.00211	208708_x_at	eukaryotic translation initiation factor 5
PDIA4	1.9	0.00211	208658_at	protein disulfide isomerase family A, member 4
SHC3	-3.106	0.00212	229824_at	SHC (Src homology 2 domain containing) transforming protein 3
FILIP1	-2.592	0.00212	1570515_a_at	filamin A interacting protein 1
DDX18	0.992	0.00212	208897_s_at	DEAD (Asp-Glu-Ala-Asp) box polypeptide 18
SNRPG	1.064	0.00212	205644_s_at	small nuclear ribonucleoprotein polypeptide G
TMX2	1.086	0.00212	201175_at	thioredoxin-related transmembrane protein 2
SNURF	1.274	0.00212	206042_x_at	paternally expressed transcript PAR-SN /// small nucleolar RNA, C/D box 107 /// small nuclear ribonucleoprotein polypeptide N /// SNRPN upstream reading frame
AP1G1	1.002	0.00212	203350_at	adaptor-related protein complex 1, gamma 1 subunit
TNPO1	1.079	0.00212	225765_at	transportin 1
FBXO22	1.538	0.00212	225734_at	F-box protein 22
NUP54	-0.555	0.00212	218256_s_at	nucleoporin 54kDa
STYXL1	1.246	0.00214	232353_s_at	serine/threonine/tyrosine interacting-like 1
NR5A2	-3.719	0.00215	210174_at	nuclear receptor subfamily 5, group A, member 2
RUNX1T1	-2.199	0.0022	228827_at	runt-related transcription factor 1; translocated to, 1 (cyclin D-related)
UBFD1	1.766	0.00231	205687_at	ubiquitin family domain containing 1
BCCIP	1.731	0.00235	227896_at	BRCA2 and CDKN1A interacting protein
EGR3	-5.281	0.00239	206115_at	early growth response 3
RASL12	-2.426	0.00239	219167_at	RAS-like, family 12
PRELID1	1.36	0.00255	224232_s_at	PRELI domain containing 1
LOC643733	-1.996	0.00261	240890_at	caspase 4, apoptosis-related cysteine peptidase pseudogene
RUNDC3B	-3.427	0.00261	241703_at	RUN domain containing 3B
ANXA4	1.425	0.00261	201301_s_at	annexin A4
LAMA1	3.08	0.00261	227048_at	laminin, alpha 1
CDH4	3.855	0.00265	206866_at	cadherin 4, type 1, R-cadherin (retinal)
UBE2H	0.913	0.00269	222420_s_at	ubiquitin-conjugating enzyme E2H
GALNT10	1.845	0.00291	212256_at	UDP-N-acetyl-alpha-D-galactosamine:polypeptide N-acetylgalactosaminyltransferase 10 (GalNAc-T10)
SUMF2	0.511	0.00292	225002_s_at	sulfatase modifying factor 2
B4GALT4	1.695	0.00293	212876_at	UDP-Gal:betaGlcNAc beta 1,4- galactosyltransferase, polypeptide 4
GART	0.891	0.00299	212378_at	phosphoribosylglycinamide formyltransferase, phosphoribosylglycinamide synthetase, phosphoribosylaminoimidazole synthetase
PAWR	1.831	0.00299	204004_at	PRKC, apoptosis, WT1, regulator
KANK3	-2.343	0.00301	213715_s_at	KN motif and ankyrin repeat domains 3
MANF	1.206	0.00301	202655_at	mesencephalic astrocyte-derived neurotrophic factor
MPDZ	-1.673	0.0031	205079_s_at	multiple PDZ domain protein
FGD6	2.087	0.0031	1555137_a_at	FYVE, RhoGEF and PH domain containing 6
SND1-IT1	0.987	0.00311	210109_at	SND1 intronic transcript 1 (non-protein coding)
COQ7	1.048	0.00315	210820_x_at	coenzyme Q7 homolog, ubiquinone (yeast)
PSMD2	0.783	0.00318	200830_at	proteasome (prosome, macropain) 26S subunit, non-ATPase, 2
MAGED1	0.934	0.00319	209014_at	melanoma antigen family D, 1
PREX2	-2.748	0.00323	228692_at	phosphatidylinositol-3,4,5-trisphosphate-dependent Rac exchange factor 2
EGR1	-3.219	0.00324	201694_s_at	early growth response 1
TFEB	-0.977	0.00324	50221_at	transcription factor EB
ATP5SL	-0.542	0.00324	45828_at	ATP5S-like
PSMD1	0.962	0.00324	201198_s_at	proteasome (prosome, macropain) 26S subunit, non-ATPase, 1
SEC13	1.096	0.00324	207707_s_at	SEC13 homolog (S. cerevisiae)
TIMM23	1.378	0.00324	218118_s_at	translocase of inner mitochondrial membrane 23 homolog (yeast)
PFN2	1.701	0.00324	204992_s_at	profilin 2
TAPT1	-1.103	0.00332	227407_at	transmembrane anterior posterior transformation 1
NPEPPS	0.75	0.00332	201454_s_at	uncharacterized LOC100653042 /// aminopeptidase puromycin sensitive
PNPT1	1.15	0.00332	225291_at	polyribonucleotide nucleotidyltransferase 1
MTDH	0.952	0.00336	212250_at	metadherin
GTF2E1	0.718	0.00337	205930_at	general transcription factor IIE, polypeptide 1, alpha 56kDa
FAM162B	-3.161	0.00341	228875_at	family with sequence similarity 162, member B
CYYR1	-2.178	0.00341	228665_at	cysteine/tyrosine-rich 1
MID2	-1.397	0.00341	209733_at	midline 2
RHEB	0.845	0.00341	201453_x_at	Ras homolog enriched in brain
CD83	-2.199	0.00344	204440_at	CD83 molecule
CD1C	-2.851	0.00344	205987_at	CD1c molecule
NUDC	0.955	0.00344	210574_s_at	nuclear distribution C homolog (A. nidulans)
ZNF273	1.778	0.00345	243661_at	zinc finger protein 273
CNIH4	1.195	0.00349	228437_at	cornichon homolog 4 (Drosophila)
PARM1	-2.905	0.00365	225809_at	prostate androgen-regulated mucin-like protein 1
FAM13C	-2.676	0.00365	1554547_at	family with sequence similarity 13, member C
NF1	0.602	0.00365	212676_at	neurofibromin 1
CKAP2	1.513	0.00365	218252_at	cytoskeleton associated protein 2

HTT	-1.165	0.0037	202390_s_at	huntingtin
DYNC1I2	0.305	0.00375	211684_s_at	dynein, cytoplasmic 1, intermediate chain 2
SNX19	0.879	0.00375	202359_s_at	sorting nexin 19
RGS5	-2.138	0.00378	218353_at	regulator of G-protein signaling 5
SLC25A13	0.9	0.0038	203775_at	solute carrier family 25 (aspartate/glutamate carrier), member 13
DCAKD	0.675	0.00386	224522_s_at	dephospho-CoA kinase domain containing
ADCY4	-2.049	0.00387	230800_at	adenylate cyclase 4
UGGT1	0.945	0.00391	222569_at	UDP-glucose glycoprotein glucosyltransferase 1
C5orf43	0.81	0.00401	224740_at	chromosome 5 open reading frame 43
PRTFDC1	-1.443	0.00407	222803_at	phosphoribosyl transferase domain containing 1
RAPH1	-1.401	0.00407	225189_s_at	Ras association (RalGDS/AF-6) and pleckstrin homology domains 1
PLD1	-1.747	0.00415	226636_at	phospholipase D1, phosphatidylcholine-specific
MRPL3	1.115	0.00424	208787_at	mitochondrial ribosomal protein L3
PMP22	-1.285	0.00425	210139_s_at	peripheral myelin protein 22
TMEM209	1.004	0.00432	235158_at	transmembrane protein 209
FCER1A	-3.059	0.00439	211734_s_at	Fc fragment of IgE, high affinity I, receptor for; alpha polypeptide
ECSCR	-2.433	0.00439	227780_s_at	endothelial cell surface expressed chemotaxis and apoptosis regulator
HOXA5	-2.282	0.00439	213844_at	homeobox A5
LOC10050565	-2.155	0.00439	230381_at	chromosome 1 open reading frame 186 /// uncharacterized LOC100505650
NCALD	-1.954	0.00439	211685_s_at	neurocalcin delta
AHR	-1.833	0.00439	202820_at	aryl hydrocarbon receptor
ZDHHC5	0.737	0.00439	224858_at	zinc finger, DHHC-type containing 5
MIR3652	0.919	0.00439	200598_s_at	heat shock protein 90kDa beta (Grp94), member 1 /// microRNA 3652
PSPH	1.426	0.00439	205194_at	phosphoserine phosphatase
DOCK1	1.857	0.00439	241708_at	dedicator of cytokinesis 1
DTNA	-2.169	0.00439	227084_at	dystrobrevin, alpha
CCDC99	1.25	0.00439	221685_s_at	coiled-coil domain containing 99
DOCK5	1.652	0.00439	230263_s_at	dedicator of cytokinesis 5
CCT5	1.717	0.00439	208696_at	chaperonin containing TCP1, subunit 5 (epsilon)
F8	-2.313	0.00439	205756_s_at	coagulation factor VIII, procoagulant component
KIAA1377	-1.874	0.00439	235956_at	KIAA1377
FANCF	1.125	0.00439	218689_at	Fanconi anemia, complementation group F
KCTD4	2.582	0.00439	240512_x_at	potassium channel tetramerisation domain containing 4
PRKG1	-2.339	0.00441	228396_at	protein kinase, cGMP-dependent, type I
TIMM17A	0.794	0.00441	215171_s_at	translocase of inner mitochondrial membrane 17 homolog A (yeast)
FICD	1.542	0.00441	219910_at	FIC domain containing
KITLG	-1.786	0.00443	226534_at	KIT ligand
TEAD1	0.877	0.00443	224955_at	TEA domain family member 1 (SV40 transcriptional enhancer factor)
TXNL4A	0.973	0.00443	202836_s_at	thioredoxin-like 4A
PARPBP	1.141	0.00443	220060_s_at	PARP1 binding protein
PPAPDC1B	1.536	0.00448	223569_at	phosphatidic acid phosphatase type 2 domain containing 1B
PELI2	-3.008	0.00449	219132_at	pellino E3 ubiquitin protein ligase family member 2
ITGA8	-2.939	0.00449	214265_at	integrin, alpha 8
LDB2	-2.483	0.00449	206481_s_at	LIM domain binding 2
FNIP2	-1.909	0.00449	225922_at	folliculin interacting protein 2
PECAM1	-1.79	0.00449	208982_at	platelet/endothelial cell adhesion molecule 1
CCT8	0.435	0.00449	200873_s_at	chaperonin containing TCP1, subunit 8 (theta)
YIF1A	1.144	0.00449	202418_at	Yip1 interacting factor homolog A (S. cerevisiae)
EGFR	1.177	0.00449	201983_s_at	epidermal growth factor receptor
SCCPDH	1.867	0.00449	201826_s_at	saccharopine dehydrogenase (putative)
PDE5A	-2.268	0.00451	227088_at	phosphodiesterase 5A, cGMP-specific
PLVAP	-1.836	0.00451	221529_s_at	plasmalemma vesicle associated protein
MGST2	-1.023	0.00454	204168_at	microsomal glutathione S-transferase 2
YME1L1	0.945	0.00454	201351_s_at	YME1-like 1 (S. cerevisiae)
OGFOD1	1.045	0.00454	225106_s_at	2-oxoglutarate and iron-dependent oxygenase domain containing 1
CENPC1	-1.114	0.00459	204739_at	centromere protein C 1
REM1	-1.587	0.00461	210300_at	RAS (RAD and GEM)-like GTP-binding 1
GMNN	1.239	0.00461	218350_s_at	geminin, DNA replication inhibitor
SRPRB	1.572	0.00466	227737_at	signal recognition particle receptor, B subunit
DLC1	-2.198	0.00475	224822_at	deleted in liver cancer 1
SARM1	2.837	0.00477	213259_s_at	sterile alpha and TIR motif containing 1
ZNF204P	-2.305	0.00479	214823_at	zinc finger protein 204, pseudogene
DYNLRB1	0.639	0.00479	217918_at	dynein, light chain, roadblock-type 1
ZNF608	-2.014	0.00487	229817_at	zinc finger protein 608
NUDT5	1.169	0.00492	223100_s_at	nudix (nucleoside diphosphate linked moiety X)-type motif 5
CCDC69	-2.384	0.00492	212886_at	coiled-coil domain containing 69
A2M	-1.81	0.00499	217757_at	alpha-2-macroglobulin
ZWILCH	1.32	0.00499	222606_at	Zwilch, kinetochore associated, homolog (Drosophila)
WDR4	0.897	0.005	241937_s_at	WD repeat domain 4
PPIB	1.511	0.005	200968_s_at	peptidylprolyl isomerase B (cyclophilin B)
SLITRK4	2.705	0.005	230680_at	SLIT and NTRK-like family, member 4
SOS1	-0.773	0.00501	212780_at	son of sevenless homolog 1 (Drosophila)
LINC00312	-2.777	0.00504	220244_at	long intergenic non-protein coding RNA 312
IL1RAP	1.79	0.00506	205227_at	interleukin 1 receptor accessory protein
SYNPO2	-2.267	0.00508	225720_at	synaptopodin 2

MANSC1	-1.682	0.00514	220945_x_at	MANSC domain containing 1
PDE4A	-1.304	0.00514	204735_at	phosphodiesterase 4A, cAMP-specific
PTPN14	1.4	0.00514	244533_at	protein tyrosine phosphatase, non-receptor type 14
RGS2	-3.135	0.00516	202388_at	regulator of G-protein signaling 2, 24kDa
ERG	-2.636	0.00516	213541_s_at	v-ets erythroblastosis virus E26 oncogene homolog (avian)
NACC2	1.217	0.00524	212993_at	NACC family member 2, BEN and BTB (POZ) domain containing
FMO3	-3.168	0.00524	40665_at	flavin containing monooxygenase 3
GTPBP4	0.709	0.00524	218239_s_at	GTP binding protein 4
TMED2	0.807	0.00524	200087_s_at	transmembrane emp24 domain trafficking protein 2
STRBP	1.348	0.00524	233252_s_at	spermatid perinuclear RNA binding protein
RSPO3	-3.724	0.00525	228186_s_at	R-spondin 3
SPRY1	-2.914	0.00525	212558_at	sprouty homolog 1, antagonist of FGF signaling (Drosophila)
SH3BGRL2	-2.056	0.00525	225354_s_at	SH3 domain binding glutamic acid-rich protein like 2
PTDSS2	0.696	0.00525	221005_s_at	phosphatidylserine synthase 2
MPHOSPH9	1.117	0.00525	1558369_at	M-phase phosphoprotein 9
ZKSCAN1	1.159	0.00525	1557953_at	zinc finger with KRAB and SCAN domains 1
ILF2	0.961	0.00527	200052_s_at	interleukin enhancer binding factor 2, 45kDa
SNRPC	0.901	0.00528	201342_at	small nuclear ribonucleoprotein polypeptide C
CPED1	-2.079	0.0053	228728_at	cadherin-like and PC-esterase domain containing 1
FLJ35934	2.228	0.00536	1564383_s_at	FLJ35934
PLA2G4C	-2.291	0.00541	209785_s_at	phospholipase A2, group IVC (cytosolic, calcium-independent)
MTUS1	-1.544	0.00541	212095_s_at	microtubule associated tumor suppressor 1
HSPA4L	1.733	0.00541	205543_at	heat shock 70kDa protein 4-like
CSE1L	1.152	0.00545	201111_at	CSE1 chromosome segregation 1-like (yeast)
RFC4	1.578	0.00547	204023_at	replication factor C (activator 1) 4, 37kDa
C19orf21	2.136	0.00547	212925_at	chromosome 19 open reading frame 21
FXYD6	-2.554	0.0056	217897_at	FXYD domain containing ion transport regulator 6
ZNF148	0.8	0.0056	203319_s_at	zinc finger protein 148
CCT3	0.972	0.0056	200910_at	chaperonin containing TCP1, subunit 3 (gamma)
C16orf54	-2.398	0.00567	1559584_a_at	chromosome 16 open reading frame 54
PSMD7	0.456	0.00567	201705_at	proteasome (prosome, macropain) 26S subunit, non-ATPase, 7
DTYMK	0.778	0.00567	203270_at	deoxythymidylate kinase (thymidylate kinase)
NFIB	-1.558	0.00573	209290_s_at	nuclear factor I/B
WDR12	0.837	0.00573	218512_at	WD repeat domain 12
BMP2	-3.63	0.00574	205289_at	bone morphogenetic protein 2
LRRC70	-2.564	0.00574	238488_at	importin 11 /// leucine rich repeat containing 70
SHANK3	-2.262	0.00574	227923_at	SH3 and multiple ankyrin repeat domains 3
HSPA12B	-2.177	0.00574	229172_at	heat shock 70kD protein 12B
GNG11	-2.151	0.00574	204115_at	guanine nucleotide binding protein (G protein), gamma 11
RBMS3	-1.785	0.00574	235570_at	RNA binding motif, single stranded interacting protein 3
TCF4	-1.292	0.00574	228837_at	transcription factor 4
INPP5A	-1.248	0.00574	203006_at	inositol polyphosphate-5-phosphatase, 40kDa
RC3H1	-0.806	0.00574	225893_at	ring finger and CCCH-type domains 1
SEC22A	0.51	0.00574	218703_at	SEC22 vesicle trafficking protein homolog A (S. cerevisiae)
NSMCE1	0.772	0.00574	224666_at	non-SMC element 1 homolog (S. cerevisiae)
AGGF1	0.8	0.00574	218534_s_at	angiogenic factor with G patch and FHA domains 1
GTF2A2	0.943	0.0058	202678_at	general transcription factor IIA, 2, 12kDa
RPL26L1	1.025	0.00585	218830_at	ribosomal protein L26-like 1
CCT2	0.771	0.00586	201947_s_at	chaperonin containing TCP1, subunit 2 (beta)
NUP37	1.074	0.00593	218622_at	nucleoporin 37kDa
C4orf32	-2.11	0.00593	227856_at	chromosome 4 open reading frame 32
PDIA3	1.192	0.00593	208612_at	protein disulfide isomerase family A, member 3
GTF2H2	1.976	0.00593	223758_s_at	general transcription factor IIH, polypeptide 2, 44kDa
DSP	3.108	0.00593	200606_at	desmoplakin
RBM14	0.396	0.00595	204178_s_at	RNA binding motif protein 14
SCARF1	-1.803	0.00598	206995_x_at	scavenger receptor class F, member 1
CDK2AP1	0.625	0.00598	201938_at	cyclin-dependent kinase 2 associated protein 1
GATA2	-2.293	0.00599	209710_at	GATA binding protein 2
IGSF10	-3.474	0.00604	230670_at	immunoglobulin superfamily, member 10
ELTD1	-2.179	0.00605	219134_at	EGF, latrophilin and seven transmembrane domain containing 1
NSUN2	0.815	0.00606	223076_s_at	NOP2/Sun RNA methyltransferase family, member 2
MLLT6	-0.939	0.00609	224784_at	myeloid/lymphoid or mixed-lineage leukemia (trithorax homolog, Drosophila); translocated to, 6
PGM5-AS1	-3.308	0.00617	230595_at	uncharacterized LOC100653336 /// PGM5 antisense RNA 1 (non-protein coding)
SNORA28	-1.262	0.00617	241843_at	eukaryotic translation initiation factor 5 /// small nucleolar RNA, H/ACA box 28
KIAA1217	1.547	0.00617	1554438_at	KIAA1217
GGPS1	0.689	0.00623	202322_s_at	geranylgeranyl diphosphate synthase 1
WDR41	1.282	0.00623	222503_s_at	WD repeat domain 41
KCNJ8	-1.662	0.00625	205304_s_at	potassium inwardly-rectifying channel, subfamily J, member 8
CC2D2A	-1.287	0.00625	234936_s_at	coiled-coil and C2 domain containing 2A
CNOT1	0.511	0.00629	200860_s_at	CCR4-NOT transcription complex, subunit 1
CACYBP	1.083	0.00629	210691_s_at	calcyclin binding protein
TNPO3	0.665	0.00632	212318_at	transportin 3
MYCBP	1.089	0.00635	203360_s_at	gap junction protein, alpha 9, 59kDa /// GJA9-MYCBP readthrough /// c-myc binding protein
GLI2	1.505	0.00635	228537_at	GLI family zinc finger 2
LMOD1	-2.966	0.00664	203766_s_at	leiomodin 1 (smooth muscle)

ZFP36	-3.253	0.00672	201531_at	zinc finger protein 36, C3H type, homolog (mouse)
KLF9	-1.72	0.00674	230636_s_at	Kruppel-like factor 9
TMEM9	1.66	0.00675	222987_s_at	transmembrane protein 9
S100A4	-1.738	0.00678	203186_s_at	S100 calcium binding protein A4
MEMO1	0.749	0.00682	219065_s_at	dpy-30 homolog (C. elegans) /// mediator of cell motility 1
FUT1	-2.302	0.00683	206109_at	fucosyltransferase 1 (galactoside 2-alpha-L-fucosyltransferase, H blood group)
COG5	0.581	0.00683	203629_s_at	component of oligomeric golgi complex 5
NUDT15	0.76	0.00689	219347_at	nudix (nucleoside diphosphate linked moiety X)-type motif 15
NHP2	1.044	0.0069	209104_s_at	NHP2 ribonucleoprotein homolog (yeast)
LSM7	1.081	0.0069	204559_s_at	LSM7 homolog, U6 small nuclear RNA associated (S. cerevisiae)
FAM136A	0.686	0.0069	215947_s_at	family with sequence similarity 136, member A
HN1L	1.039	0.00694	212109_at	hematological and neurological expressed 1-like
LINC00341	-2.275	0.00698	219563_at	long intergenic non-protein coding RNA 341
B3GAT3	0.777	0.00698	35179_at	beta-1,3-glucuronyltransferase 3 (glucuronosyltransferase I)
GPR116	-2.623	0.00699	212950_at	G protein-coupled receptor 116
TNS1	-1.831	0.00699	221748_s_at	tensin 1
FRAT1	-0.991	0.00699	219889_at	frequently rearranged in advanced T-cell lymphomas
PHB	1.089	0.00699	200658_s_at	prohibitin
MECOM	-2.921	0.00703	226420_at	MDS1 and EVI1 complex locus
RASIP1	-2.66	0.00706	220027_s_at	Ras interacting protein 1
PIGB	0.85	0.00706	242760_x_at	phosphatidylinositol glycan anchor biosynthesis, class B
LMNB2	1.668	0.00714	216952_s_at	lamin B2
CYGB	-1.963	0.00719	226632_at	cytoglobin
NME1	1.752	0.00723	201577_at	NME/NM23 nucleoside diphosphate kinase 1
POGK	0.6	0.00728	218229_s_at	pogo transposable element with KRAB domain
HCCS	0.557	0.00739	203745_at	holocytochrome c synthase
UHMK1	1.095	0.0074	235003_at	U2AF homology motif (UHM) kinase 1
PHLDA1	-2.504	0.00743	217996_at	pleckstrin homology-like domain, family A, member 1
HHEX	-1.934	0.00743	204689_at	hematopoietically expressed homeobox
CYSLTR1	-1.614	0.00743	230866_at	cysteinyl leukotriene receptor 1
PWP1	0.702	0.00743	201608_s_at	PWP1 homolog (S. cerevisiae)
TRIM27	0.754	0.00743	212116_at	tripartite motif containing 27
TIMELESS	1.358	0.00743	203046_s_at	timeless homolog (Drosophila)
ZNF738	1.716	0.00743	229700_at	zinc finger protein 738
COBLL1	1.825	0.00743	203642_s_at	COBL-like 1
PDPN	2.246	0.00743	226658_at	podoplanin
ARID5B	-1.52	0.00747	212614_at	AT rich interactive domain 5B (MRF1-like)
GEM	-2.295	0.0075	204472_at	GTP binding protein overexpressed in skeletal muscle
ORC5	0.806	0.00751	211212_s_at	origin recognition complex, subunit 5
JAM2	-2.302	0.00753	219213_at	junctional adhesion molecule 2
ZNF79	0.447	0.00753	216486_x_at	zinc finger protein 79
PFDN6	0.957	0.00754	233588_x_at	prefoldin subunit 6
RBBP4	0.82	0.00762	217301_x_at	retinoblastoma binding protein 4
RCAN2	-2.506	0.00763	203498_at	regulator of calcineurin 2
ING2	-0.791	0.00765	205981_s_at	inhibitor of growth family, member 2
TSC22D3	-1.714	0.00767	208763_s_at	TSC22 domain family, member 3
COL21A1	-2.605	0.00775	208096_s_at	collagen, type XXI, alpha 1
UMPS	0.705	0.00775	202706_s_at	uridine monophosphate synthetase
AATF	0.775	0.00777	209165_at	apoptosis antagonizing transcription factor
SLC35A2	1.146	0.00778	209326_at	solute carrier family 35 (UDP-galactose transporter), member A2
SLC30A6	0.684	0.00779	243643_x_at	solute carrier family 30 (zinc transporter), member 6
RNF180	-2.638	0.0078	242033_at	ring finger protein 180
RER1	0.802	0.0078	202297_s_at	RER1 retention in endoplasmic reticulum 1 homolog (S. cerevisiae)
LSM12	0.989	0.0078	212532_s_at	LSM12 homolog (S. cerevisiae)
COPB2	0.907	0.00782	201098_at	coatomer protein complex, subunit beta 2 (beta prime)
CKAP5	0.752	0.00784	212832_s_at	cytoskeleton associated protein 5
TMEM135	0.853	0.00784	208810_at	DnaJ (Hsp40) homolog, subfamily B, member 6 /// transmembrane protein 135
EI24	0.623	0.00792	216396_s_at	etoposide induced 2.4 mRNA
RRM1	0.727	0.00792	201477_s_at	ribonucleotide reductase M1
MAGI3	-1.932	0.00794	226770_at	membrane associated guanylate kinase, WW and PDZ domain containing 3
AP3M1	0.63	0.00796	222516_at	adaptor-related protein complex 3, mu 1 subunit
HPGD	-4.167	0.008	203914_x_at	hydroxyprostaglandin dehydrogenase 15-(NAD)
P2RY14	-2.691	0.008	206637_at	purinergic receptor P2Y, G-protein coupled, 14
IL33	-2.65	0.008	209821_at	interleukin 33
PER3	-2.243	0.008	221045_s_at	period homolog 3 (Drosophila)
ZNF704	-1.912	0.008	223366_at	zinc finger protein 704
CBX7	-1.474	0.008	212914_at	chromobox homolog 7
NR3C1	-0.721	0.008	201865_x_at	nuclear receptor subfamily 3, group C, member 1 (glucocorticoid receptor)
AP3D1	0.627	0.008	206592_s_at	adaptor-related protein complex 3, delta 1 subunit
CYB5B	0.781	0.008	201634_s_at	cytochrome b5 type B (outer mitochondrial membrane)
MRPL17	0.873	0.008	222216_s_at	mitochondrial ribosomal protein L17
TMEM48	0.893	0.008	234672_s_at	transmembrane protein 48
MEA1	0.922	0.008	218061_at	male-enhanced antigen 1
RPS27L	1.104	0.008	222487_s_at	ribosomal protein S27-like
MESDC2	1.107	0.008	224679_at	mesoderm development candidate 2

LMBR1	1.25	0.008	224410_s_at	limb region 1 homolog (mouse)
UCK2	1.318	0.008	209825_s_at	microRNA 3658 /// uridine-cytidine kinase 2
RAN	1.456	0.008	200750_s_at	RAN, member RAS oncogene family
NUP155	1.542	0.008	206550_s_at	nucleoporin 155kDa
OIP5	1.93	0.008	213599_at	Opa interacting protein 5
SH3RF2	3.465	0.008	228892_at	SH3 domain containing ring finger 2
COPG1	0.919	0.008	217749_at	coatomer protein complex, subunit gamma 1
SEC61A1	1.258	0.00804	217716_s_at	Sec61 alpha 1 subunit (S. cerevisiae)
CENPN	2.784	0.0081	219555_s_at	centromere protein N
SMC2	0.977	0.00814	213253_at	structural maintenance of chromosomes 2
CD109	-2.036	0.00823	226545_at	CD109 molecule
BTG1	-1.11	0.00828	200921_s_at	B-cell translocation gene 1, anti-proliferative
NTMT1	1.233	0.0083	223369_at	N-terminal Xaa-Pro-Lys N-methyltransferase 1
FILIP1L	-2.017	0.00831	204135_at	filamin A interacting protein 1-like
SLC10A7	-0.692	0.00831	235143_at	solute carrier family 10 (sodium/bile acid cotransporter family), member 7
PTPRO	-1.156	0.00837	211600_at	protein tyrosine phosphatase, receptor type, O
SLC6A16	-1.557	0.00838	1569940_at	Solute carrier family 6, member 16
FANCA	1.689	0.00839	236976_at	Fanconi anemia, complementation group A
ACSL5	-1.667	0.00846	218322_s_at	acyl-CoA synthetase long-chain family member 5
TMEM199	0.613	0.00846	225374_at	microRNA 4723 /// transmembrane protein 199
TMPO	1.713	0.00846	203432_at	thymopoietin
SNAPC5	0.799	0.00846	213203_at	small nuclear RNA activating complex, polypeptide 5, 19kDa
TFAP2A	4.614	0.00847	204653_at	transcription factor AP-2 alpha (activating enhancer binding protein 2 alpha)
FARSA	1.09	0.00857	202159_at	phenylalanyl-tRNA synthetase, alpha subunit
G3BP1	1.148	0.00857	201514_s_at	GTPase activating protein (SH3 domain) binding protein 1
OIP5-AS1	1.305	0.00857	231808_at	OIP5 antisense RNA 1 (non-protein coding)
NTAN1	-0.974	0.00857	213061_s_at	N-terminal asparagine amidase
ILF3	1.05	0.00857	217805_at	interleukin enhancer binding factor 3, 90kDa
CALU	1.454	0.00857	200755_s_at	calumenin
ZMYM4	0.461	0.00862	202049_s_at	zinc finger, MYM-type 4
KHNYN	-0.923	0.00864	212356_at	KH and NYN domain containing
FHL5	-3.202	0.00866	220170_at	four and a half LIM domains 5
EPM2A	-1.21	0.00872	205231_s_at	epilepsy, progressive myoclonus type 2A, Lafora disease (laforin)
GALM	-1.135	0.00874	234974_at	galactose mutarotase (aldose 1-epimerase)
IPP	1.275	0.00877	1554740_a_at	intracisternal A particle-promoted polypeptide
PHF17	-0.98	0.0088	225816_at	PHD finger protein 17
THRB	-2.541	0.00884	228716_at	thyroid hormone receptor, beta
LOC10050581	-1.432	0.00885	242521_at	uncharacterized LOC100505812
GCN1L1	0.968	0.00885	216232_s_at	GCN1 general control of amino-acid synthesis 1-like 1 (yeast)
AIMP2	0.749	0.00889	202138_x_at	aminoacyl tRNA synthetase complex-interacting multifunctional protein 2
DNALI1	-2.049	0.00889	227081_at	dynein, axonemal, light intermediate chain 1
DCAF13	0.896	0.00891	225676_s_at	DDB1 and CUL4 associated factor 13
RREB1	-0.834	0.00892	228487_s_at	Ras responsive element binding protein 1
NCBP2	1.325	0.00892	201521_s_at	nuclear cap binding protein subunit 2, 20kDa
MNF1	1.37	0.00892	224448_s_at	mitochondrial nucleoid factor 1
PRKCI	1.407	0.00894	213518_at	protein kinase C, iota
PPM1F	-1.128	0.00895	203063_at	protein phosphatase, Mg2+/Mn2+ dependent, 1F
FAM126A	-1.547	0.00903	227239_at	family with sequence similarity 126, member A
CNRIP1	-1.804	0.00905	226751_at	cannabinoid receptor interacting protein 1
SCAMP3	0.821	0.00906	201771_at	secretory carrier membrane protein 3
PRDM1	-1.771	0.00909	228964_at	PR domain containing 1, with ZNF domain
C9orf72	-1.435	0.00909	225919_s_at	chromosome 9 open reading frame 72
SAMD13	-1.032	0.00909	229402_at	sterile alpha motif domain containing 13
IQSEC1	-0.935	0.00909	203906_at	IQ motif and Sec7 domain 1
AAGAB	1.094	0.00909	202852_s_at	alpha- and gamma-adaptin binding protein
TMEM100	-3.266	0.00913	219230_at	transmembrane protein 100
C5orf51	0.844	0.00919	226159_at	chromosome 5 open reading frame 51
WDR5B	0.968	0.00919	219538_at	WD repeat domain 5B
PID1	-2.534	0.00921	219093_at	phosphotyrosine interaction domain containing 1
LSM5	0.855	0.00933	211747_s_at	LSM5 homolog, U6 small nuclear RNA associated (S. cerevisiae)
WNT3	1.945	0.00935	229103_at	wingless-type MMTV integration site family, member 3
MYCT1	-2.429	0.00942	231947_at	myc target 1
TMCO1	0.719	0.00943	208716_s_at	transmembrane and coiled-coil domains 1
TPSB2	-2.818	0.00952	216474_x_at	tryptase alpha/beta 1 /// tryptase beta 2 (gene/pseudogene)
GORASP2	0.402	0.00952	208843_s_at	golgi reassembly stacking protein 2, 55kDa
SLC39A6	0.779	0.00952	202088_at	solute carrier family 39 (zinc transporter), member 6
LRCH1	-1.46	0.00956	226795_at	leucine-rich repeats and calponin homology (CH) domain containing 1
MCM4	1.523	0.00957	222036_s_at	minichromosome maintenance complex component 4
TPTE2P2	-1.555	0.00958	219477_s_at	mitochondrial ribosomal protein S31 pseudogene 3 /// thrombospondin, type I, domain containing 1 /// thrombospondin, type I, domain containing 1 pseudogene 1 /// transmembrane phosphoinositide 3-phosphatase and tensin homolog 2 pseudogene 2
HNMT	-1.518	0.00958	228772_at	histamine N-methyltransferase
C11orf48	0.549	0.0096	221637_s_at	chromosome 11 open reading frame 48
PRKAR1A	1.406	0.00961	200604_s_at	protein kinase, cAMP-dependent, regulatory, type I, alpha (tissue specific extinguisher 1)
ADORA3	1.912	0.00961	206171_at	adenosine A3 receptor
PRCP	-1.33	0.00964	201494_at	prolylcarboxypeptidase (angiotensinase C)
PSMA7	0.931	0.00964	216088_s_at	proteasome (prosome, macropain) subunit, alpha type, 7

LIN7A	-2.193	0.00972	227929_at	lin-7 homolog A (C. elegans)
LYST	-1.582	0.00972	203518_at	lysosomal trafficking regulator
DDX23	0.355	0.00972	40465_at	DEAD (Asp-Glu-Ala-Asp) box polypeptide 23
TTC30B	0.781	0.00972	243413_at	tetratricopeptide repeat domain 30B
PGK1	0.901	0.00972	200738_s_at	uncharacterized LOC100652805 /// uncharacterized LOC100653302 /// phosphoglycerate kinase 1
AKIP1	1.059	0.00972	219953_s_at	A kinase (PRKA) interacting protein 1
ANK2	-3.238	0.00978	202920_at	ankyrin 2, neuronal
CDK11B	-0.561	0.00978	212401_s_at	cyclin-dependent kinase 11A /// cyclin-dependent kinase 11B
ZNF138	0.909	0.00978	244743_x_at	zinc finger protein 138
FAM149B1	1.242	0.00979	213896_x_at	family with sequence similarity 149, member B1
ADAMTS9-AS:	-2.268	0.00983	1556364_at	ADAMTS9 antisense RNA 2 (non-protein coding)
MKL2	-1.494	0.00983	218259_at	MKL/myocardin-like 2
NMT1	0.926	0.00983	201157_s_at	N-myristoyltransferase 1
SSR3	1.262	0.00983	222411_s_at	signal sequence receptor, gamma (translocon-associated protein gamma)
AKT3	-1.656	0.00991	212607_at	v-akt murine thymoma viral oncogene homolog 3 (protein kinase B, gamma)
PTGES	2.777	0.00991	210367_s_at	prostaglandin E synthase
ANGPT1	-3	0.00991	205609_at	angiopoietin 1
DNAJC9	1.18	0.00997	213088_s_at	DnaJ (Hsp40) homolog, subfamily C, member 9
PER1	-2.127	0.00999	202861_at	period homolog 1 (Drosophila)
SETD1B	-0.524	0.0101	213153_at	SET domain containing 1B
ALDH1A1	-2.948	0.0101	212224_at	aldehyde dehydrogenase 1 family, member A1
C18orf34	-3.022	0.0101	1558820 a at	chromosome 18 open reading frame 34
ECE1	-1.241	0.0102	201749_at	endothelin converting enzyme 1
RPP38	0.838	0.0102	205562_at	ribonuclease P/MRP 38kDa subunit
MCM2	1.681	0.0102	202107_s_at	minichromosome maintenance complex component 2
CD34	-2.408	0.0102	209543_s_at	CD34 molecule
GINS1	2.074	0.0102	206102_at	GINS complex subunit 1 (Psf1 homolog)
STIL	1.687	0.0102	205339_at	SCL/TAL1 interrupting locus
MTA3	0.925	0.0103	223311_s_at	metastasis associated 1 family, member 3
LRRC1	2.357	0.0104	218816_at	leucine rich repeat containing 1
CBS	1.637	0.0104	1553972 a at	cystathionine-beta-synthase
RNF125	-2.006	0.0106	235199_at	ring finger protein 125, E3 ubiquitin protein ligase
UBL5	0.993	0.0106	218011_at	ubiquitin-like 5
NDFIP2	1.908	0.0107	224799_at	Nedd4 family interacting protein 2
EIF3J	0.531	0.0107	208985_s_at	eukaryotic translation initiation factor 3, subunit J
FEN1	1.548	0.0108	204767_s_at	flap structure-specific endonuclease 1
YIPF5	0.668	0.0109	224953_at	Yip1 domain family, member 5
RAMP3	-2.521	0.0109	205326_at	receptor (G protein-coupled) activity modifying protein 3
RFK	0.483	0.0109	203224_at	riboflavin kinase
MEIS1	-2.07	0.011	1559477 s at	Meis homeobox 1
JTB	0.571	0.011	200048_s_at	jumping translocation breakpoint
RSRC1	1.098	0.011	1555501 s at	arginine/serine-rich coiled-coil 1
TBL2	0.851	0.011	212685_s_at	transducin (beta)-like 2
CXCL2	-4.114	0.011	209774_x_at	chemokine (C-X-C motif) ligand 2
TTC14	0.659	0.011	225178_at	tetratricopeptide repeat domain 14
SIPA1L1	1.065	0.011	202255_s_at	signal-induced proliferation-associated 1 like 1
E2F7	2.341	0.011	228033_at	E2F transcription factor 7
CCDC146	-1.154	0.011	227091_at	coiled-coil domain containing 146
SSBP1	0.833	0.0111	202591_s_at	microRNA 5096 /// single-stranded DNA binding protein 1, mitochondrial
CNPY2	0.89	0.0111	202857_at	canopy 2 homolog (zebrafish)
C16orf87	1.214	0.0111	226608_at	chromosome 16 open reading frame 87
KIAA0146	1.031	0.0112	212523_s_at	KIAA0146
MALL	-2.787	0.0113	209373_at	mal, T-cell differentiation protein-like
TSG101	0.447	0.0113	201758_at	tumor susceptibility gene 101
SOX7	-2.784	0.0113	228698_at	SRY (sex determining region Y)-box 7
ZEB2	-1.31	0.0113	228333_at	zinc finger E-box binding homeobox 2
PTPRB	-2.801	0.0113	230250_at	protein tyrosine phosphatase, receptor type, B
ANGPTL1	-2.943	0.0113	231773_at	angiopoietin-like 1
C1orf115	-2.75	0.0114	218546_at	chromosome 1 open reading frame 115
SNRNP27	0.956	0.0115	212438_at	small nuclear ribonucleoprotein 27kDa (U4/U6.U5)
NACC1	0.678	0.0118	227651_at	nucleus accumbens associated 1, BEN and BTB (POZ) domain containing
TUBB	0.794	0.0118	212320_at	tubulin, beta class I
PSMB2	0.893	0.0118	200039_s_at	proteasome (prosome, macropain) subunit, beta type, 2
LAPTM4B	1.428	0.0118	208029_s_at	lysosomal protein transmembrane 4 beta
ZNF438	-0.867	0.0118	229743_at	zinc finger protein 438
HLX	-2.254	0.0118	214438_at	H2.0-like homeobox
SERF2-C15OR	0.987	0.0118	233746_x_at	chromosome 15 open reading frame 63 /// microRNA 1282 /// small EDRK-rich factor 2 /// SERF2-C15orf63 readthrough
PSMG3	0.998	0.0118	223363_at	proteasome (prosome, macropain) assembly chaperone 3
LSM4	1.255	0.0119	202737_s_at	LSM4 homolog, U6 small nuclear RNA associated (S. cerevisiae)
ZNF85	1.579	0.012	1554445_at	zinc finger protein 85
CDKN2AIP	-0.689	0.0121	218929_at	CDKN2A interacting protein
PRKCH	-1.987	0.0122	218764_at	protein kinase C, eta
SPICE1	0.847	0.0122	234995_at	spindle and centriole associated protein 1
LOC10050702	3.154	0.0122	232035_at	histone cluster 1, H4a /// histone cluster 1, H4b /// histone cluster 1, H4c /// histone cluster 1, H4d /// histone cluster 1, H4e /// histone cluster 1, H4f /// histone cluster 1, H4h /// histone cluster 1, H4i /// histone cluster 1, H4j /// histone clus
SLK	-0.858	0.0122	206875_s_at	STE20-like kinase

CYBRD1	2.129	0.0122	217889_s_at	cytochrome b reductase 1
MTMR10	-0.608	0.0122	225810_at	myotubularin related protein 10
CEP170	0.757	0.0122	212746_s_at	centrosomal protein 170kDa
NDUFS6	1.355	0.0122	203606_at	NADH dehydrogenase (ubiquinone) Fe-S protein 6, 13kDa (NADH-coenzyme Q reductase)
TFCP2	0.906	0.0123	209338_at	transcription factor CP2
EBF1	-2.775	0.0123	227646_at	early B-cell factor 1
ATXN3	-1.576	0.0124	238723_at	ataxin 3
CPA3	-4.021	0.0124	205624_at	carboxypeptidase A3 (mast cell)
DNAJB11	0.82	0.0124	223054_at	DnaJ (Hsp40) homolog, subfamily B, member 11
UCHL1	3.761	0.0125	201387_s_at	ubiquitin carboxyl-terminal esterase L1 (ubiquitin thiolesterase)
MCTP1	-2.49	0.0125	235740_at	multiple C2 domains, transmembrane 1
PTPN13	-2.614	0.0125	204201_s_at	protein tyrosine phosphatase, non-receptor type 13 (APO-1/CD95 (Fas)-associated phosphatase)
RPS6KA5	-1.107	0.0125	204633_s_at	ribosomal protein S6 kinase, 90kDa, polypeptide 5
PCNXL2	-0.747	0.0125	39650_s_at	pecanex-like 2 (Drosophila)
POLR3A	0.431	0.0125	227872_at	polymerase (RNA) III (DNA directed) polypeptide A, 155kDa
HEATR2	0.944	0.0125	218460_at	HEAT repeat containing 2
METTL2B	1.135	0.0125	225253_s_at	methyltransferase like 2A /// methyltransferase like 2B
XRCC4	1.18	0.0125	210813_s_at	X-ray repair complementing defective repair in Chinese hamster cells 4
GTF2IRD1	1.409	0.0125	218412_s_at	GTF2I repeat domain containing 1
LRRC61	1.915	0.0125	218907_s_at	leucine rich repeat containing 61
KRT14	4.485	0.0126	209351_at	keratin 14
SLMO2	0.798	0.0126	217851_s_at	slowmo homolog 2 (Drosophila)
RAD18	1.065	0.0127	223417_at	RAD18 homolog (S. cerevisiae)
EFS	-1.757	0.0127	204400_at	embryonal Fyn-associated substrate
RYK	1.014	0.0128	216976_s_at	receptor-like tyrosine kinase
SEC11A	0.44	0.0129	201290_at	SEC11 homolog A (S. cerevisiae)
LARS	0.49	0.0129	222428_s_at	leucyl-tRNA synthetase
PSMC3	0.861	0.0129	201267_s_at	proteasome (prosome, macropain) 26S subunit, ATPase, 3
TRAFD1	1.184	0.0129	35254_at	TRAF-type zinc finger domain containing 1
ARHGEF6	-1.388	0.0129	209539_at	Rac/Cdc42 guanine nucleotide exchange factor (GEF) 6
YIPF6	0.578	0.0129	212341_at	Yip1 domain family, member 6
FAM192A	0.621	0.0129	222460_s_at	family with sequence similarity 192, member A
FADD	0.937	0.0129	202535_at	Fas (TNFRSF6)-associated via death domain
CCDC90B	1.156	0.0129	1568834_s_at	coiled-coil domain containing 90B
MIS18A	1.253	0.0129	219004_s_at	MIS18 kinetochore protein homolog A (S. pombe)
ETS1	-1.396	0.0129	224833_at	v-ets erythroblastosis virus E26 oncogene homolog 1 (avian)
CENPF	2.259	0.013	209172_s_at	centromere protein F, 350/400kDa (mitosin)
RPGR	-1.331	0.013	207624_s_at	retinitis pigmentosa GTPase regulator
STAMBP	0.703	0.013	202811_at	STAM binding protein
MRPS5	0.794	0.013	224333_s_at	mitochondrial ribosomal protein S5
PSMC2	0.719	0.0131	201068_s_at	proteasome (prosome, macropain) 26S subunit, ATPase, 2
PRPF4	0.674	0.0132	209161_at	PRP4 pre-mRNA processing factor 4 homolog (yeast)
FAM149A	-2.121	0.0132	222291_at	family with sequence similarity 149, member A
KIAA0232	-0.641	0.0132	212441_at	KIAA0232
CDK7	1.446	0.0133	211297_s_at	cyclin-dependent kinase 7
SND1	0.805	0.0134	201622_at	staphylococcal nuclease and tudor domain containing 1
CRYBG3	-0.798	0.0134	214030_at	beta-gamma crystallin domain containing 3
FAM98B	0.685	0.0134	1564637_a_at	family with sequence similarity 98, member B
SLC39A10	1.057	0.0134	225295_at	solute carrier family 39 (zinc transporter), member 10
EME1	1.574	0.0134	234464_s_at	essential meiotic endonuclease 1 homolog 1 (S. pombe)
LOC10050549	-2.469	0.0134	230132_at	uncharacterized LOC100505495
LURAP1L	-2.53	0.0135	227443_at	leucine rich adaptor protein 1-like
PLCXD3	-2.861	0.0135	230081_at	phosphatidylinositol-specific phospholipase C, X domain containing 3
PIGU	1.195	0.0135	225903_at	phosphatidylinositol glycan anchor biosynthesis, class U
LRRC20	0.661	0.0136	218550_s_at	leucine rich repeat containing 20
RELL1	-1.015	0.0136	226430_at	RELT-like 1
FLJ27352	0.945	0.0136	243309_at	uncharacterized LOC145788
NIPA2	0.969	0.0136	212133_at	non imprinted in Prader-Willi/Angelman syndrome 2
LSM3	0.973	0.0136	202209_at	LSM3 homolog, U6 small nuclear RNA associated (S. cerevisiae)
CEP89	1.144	0.0136	1553732_s_at	centrosomal protein 89kDa
SLC7A1	1.697	0.0136	212295_s_at	solute carrier family 7 (cationic amino acid transporter, y+ system), member 1
ITGB4	1.771	0.0136	204990_s_at	integrin, beta 4
DAAM2	-2.055	0.0136	212793_at	dishevelled associated activator of morphogenesis 2
BRD1	-0.771	0.0136	215460_x_at	bromodomain containing 1
CDC6	1.725	0.0136	203968_s_at	cell division cycle 6 homolog (S. cerevisiae)
ESAM	-2.718	0.0136	225369_at	endothelial cell adhesion molecule
IRAK3	-2.638	0.0136	213817_at	interleukin-1 receptor-associated kinase 3
GFPT1	1.062	0.0137	226886_at	glutamine--fructose-6-phosphate transaminase 1
NOSTRIN	-2.623	0.0138	226992_at	nitric oxide synthase trafficker
ST6GALNAC3	-1.806	0.0138	235334_at	ST6 (alpha-N-acetyl-neuraminyl-2,3-beta-galactosyl-1,3)-N-acetylgalactosaminide alpha-2,6-sialyltransferase 3
FXR1	0.712	0.0138	229519_at	fragile X mental retardation, autosomal homolog 1
RAD50	1.15	0.0138	208393_s_at	RAD50 homolog (S. cerevisiae)
RPL23AP7	0.683	0.0139	221634_at	60S ribosomal protein L23a-like /// ribosomal protein L23a pseudogene /// uncharacterized LOC100508047 /// 60S ribosomal protein L23a-like /// uncharacterized LOC100653267 /// CENPB DNA-binding domains containing 1 pseudogene /// ribosomal protein L23a ps
SLC25A25	-1.596	0.0139	225212_at	solute carrier family 25 (mitochondrial carrier; phosphate carrier), member 25
SRSF9	0.774	0.0139	200044_at	glutamyl-tRNA(Gln) amidotransferase, subunit C homolog (bacterial) /// serine/arginine-rich splicing factor 9

PCDHGA10	0.871	0.0139	211875_x_at	protocadherin gamma subfamily A, 10
BBS7	-0.957	0.0139	235007_at	Bardet-Biedl syndrome 7
MRPL28	0.686	0.0139	204599_s_at	mitochondrial ribosomal protein L28
TOR1A	0.653	0.014	202349_at	torsin family 1, member A (torsin A)
PTPLAD1	0.991	0.014	234000_s_at	protein tyrosine phosphatase-like A domain containing 1
EPHA3	-3.496	0.014	206070_s_at	EPH receptor A3
DHX29	0.517	0.014	212648_at	DEAH (Asp-Glu-Ala-His) box polypeptide 29
PCDHGA8	0.692	0.014	210368_at	protocadherin gamma subfamily A, 8
KDM4A	0.844	0.014	203204_s_at	lysine (K)-specific demethylase 4A
KIAA1524	1.731	0.014	231855_at	KIAA1524
GUCY1A3	-1.983	0.014	227235_at	guanylate cyclase 1, soluble, alpha 3
RRP7A	0.915	0.014	202937_x_at	ribosomal RNA processing 7 homolog A (S. cerevisiae)
SLC9A7	2.216	0.014	226550_at	solute carrier family 9, subfamily A (NHE7, cation proton antiporter 7), member 7
KLF11	-1.269	0.0141	218486_at	Kruppel-like factor 11
PPP1R16B	-1.736	0.0141	41577_at	protein phosphatase 1, regulatory subunit 16B
RBPMS	-1.07	0.0141	209487_at	RNA binding protein with multiple splicing
NOL7	0.53	0.0141	202882_x_at	nucleolar protein 7, 27kDa
SPCS2	0.584	0.0141	201240_s_at	signal peptidase complex subunit 2 homolog (S. cerevisiae) pseudogene /// signal peptidase complex subunit 2 homolog (S. cerevisiae)
UQCC	0.938	0.0141	222470_s_at	ubiquinol-cytochrome c reductase complex chaperone
MRVI1	-1.82	0.0142	226047_at	murine retrovirus integration site 1 homolog
ABCA11P	-0.976	0.0142	220159_at	ATP-binding cassette, sub-family A (ABC1), member 11, pseudogene
RSPRY1	1.619	0.0142	225774_at	ring finger and SPRY domain containing 1
ADAMTS9	-2.741	0.0142	220287_at	ADAM metallopeptidase with thrombospondin type 1 motif, 9
PTGER4	-1.656	0.0142	204897_at	prostaglandin E receptor 4 (subtype EP4)
PRPF19	0.858	0.0142	203103_s_at	PRP19/PSO4 pre-mRNA processing factor 19 homolog (S. cerevisiae)
FANCI	2.283	0.0142	213008_at	Fanconi anemia, complementation group I
CCDC167	0.725	0.0142	225723_at	coiled-coil domain containing 167
AVIL	-0.958	0.0143	233333_x_at	advillin
UBE2V2	0.542	0.0143	209096_at	ubiquitin-conjugating enzyme E2 variant 2
COPS8	0.608	0.0143	214260_at	COP9 constitutive photomorphogenic homolog subunit 8 (Arabidopsis)
MAML3	-1.252	0.0143	242794_at	mastermind-like 3 (Drosophila)
NIN	-0.701	0.0143	225921_at	ninein (GSK3B interacting protein)
PSMC5	0.563	0.0143	209503_s_at	proteasome (prosome, macropain) 26S subunit, ATPase, 5
SRM	1.46	0.0144	201516_at	spermidine synthase
GABPB1-AS1	1.157	0.0144	227406_at	GABPB1 antisense RNA 1 (non-protein coding)
RFC5	1.185	0.0144	203209_at	replication factor C (activator 1) 5, 36.5kDa
USP31	2.052	0.0144	1558117_s_at	ubiquitin specific peptidase 31
NR1D2	-2.232	0.0144	225768_at	nuclear receptor subfamily 1, group D, member 2
FUNDC1	0.474	0.0144	235346_at	FUN14 domain containing 1
FIGN	-1.873	0.0145	238964_at	fidgetin
NAALADL1	-1.609	0.0145	228424_at	N-acetylated alpha-linked acidic dipeptidase-like 1
EPB41L4A	0.686	0.0145	220119_at	erythrocyte membrane protein band 4.1 like 4A
PDF	0.705	0.0145	219575_s_at	component of oligomeric golgi complex 8 /// peptide deformylase (mitochondrial)
ISG20	1.985	0.0145	204698_at	interferon stimulated exonuclease gene 20kDa
SHE	-1.856	0.0146	229910_at	Src homology 2 domain containing E
GIMAP1	-2.399	0.0147	236583_at	GTPase, IMAP family member 1
GJA4	-1.872	0.0147	40687_at	gap junction protein, alpha 4, 37kDa
RBBP9	-1.067	0.0147	226696_at	retinoblastoma binding protein 9
ATG3	0.644	0.0147	221492_s_at	autophagy related 3
SCAMP1	0.811	0.0147	206668_s_at	secretory carrier membrane protein 1
HBEGF	-2.485	0.0148	203821_at	heparin-binding EGF-like growth factor
FAM198B	-1.593	0.0148	223204_at	family with sequence similarity 198, member B
UEVLD	0.749	0.0148	220775_s_at	UEV and lactate/malate dehyrogenase domains
WAPAL	0.375	0.0148	212267_at	wings apart-like homolog (Drosophila)
B3GALTL	-1.331	0.0148	227083_at	beta 1,3-galactosyltransferase-like
ADPRHL2	0.756	0.0148	223097_at	ADP-ribosylhydrolase like 2
ADRBK2	-2.168	0.0148	228771_at	adrenergic, beta, receptor kinase 2
LOC1002890	-1.043	0.0148	215160_x_at	protein FRG1-like
CBX6	-0.773	0.0148	202047_s_at	chromobox homolog 6
STK4	-0.636	0.0148	223746_at	serine/threonine kinase 4
BBS4	0.711	0.0148	212745_s_at	Bardet-Biedl syndrome 4
TTC9C	1.055	0.0148	226175_at	tetratricopeptide repeat domain 9C
DPP3	1.276	0.0148	218567_x_at	dipeptidyl-peptidase 3
SNORA11E	1.617	0.0148	223313_s_at	melanoma antigen family D, 4 /// melanoma antigen family D, 4B /// small nucleolar RNA, H/ACA box 11D /// small nucleolar RNA, H/ACA box 11E
ARHGEF4	2.091	0.0148	205109_s_at	Rho guanine nucleotide exchange factor (GEF) 4
FCGBP	3.461	0.0148	203240_at	Fc fragment of IgG binding protein
UNC5B	1.55	0.0149	213100_at	unc-5 homolog B (C. elegans)
TENC1	-1.8	0.0149	212494_at	tensin like C1 domain containing phosphatase (tensin 2)
LOC728377	0.943	0.0149	228596_at	Rho guanine nucleotide exchange factor (GEF) 5 pseudogene
BAI2	1.068	0.0149	204966_at	brain-specific angiogenesis inhibitor 2
LOC339803	1.457	0.0149	227941_at	uncharacterized LOC339803
ACTL6A	0.597	0.0149	202666_s_at	actin-like 6A
RPS10-NUDT	0.672	0.015	221579_s_at	nudix (nucleoside diphosphate linked moiety X)-type motif 3 /// RPS10-NUDT3 readthrough
CYP39A1	-2.392	0.015	1553977_a_at	cytochrome P450, family 39, subfamily A, polypeptide 1
C12orf65	0.959	0.0151	223476_s_at	chromosome 12 open reading frame 65

SRGAP1	1.271	0.0151	233888_s_at	SLIT-ROBO Rho GTPase activating protein 1
ANXA2	0.595	0.0151	201590_x_at	annexin A2
CNOT6L	-0.638	0.0151	227119_at	CCR4-NOT transcription complex, subunit 6-like
ALG8	0.92	0.0151	203545_at	asparagine-linked glycosylation 8, alpha-1,3-glucosyltransferase homolog (S. cerevisiae)
TP53INP2	-1.22	0.0151	224836_at	tumor protein p53 inducible nuclear protein 2
ZNF641	1.259	0.0151	226509_at	zinc finger protein 641
TYMS	2.384	0.0151	202589_at	thymidylate synthetase
MIR143HG	-1.677	0.0151	231987_at	MIR143 host gene (non-protein coding)
CLCN7	0.549	0.0151	38069_at	chloride channel, voltage-sensitive 7
NAA38	0.482	0.0151	219119_at	N(alpha)-acetyltransferase 38, NatC auxiliary subunit
GPM6A	-4.373	0.0151	209470_s_at	glycoprotein M6A
CELF1	0.812	0.0151	204113_at	CUGBP, Elav-like family member 1
BRI3BP	0.838	0.0152	225716_at	BRI3 binding protein
CD40	-0.987	0.0152	222292_at	CD40 molecule, TNF receptor superfamily member 5
KDM6B	-0.567	0.0152	41386_i_at	lysine (K)-specific demethylase 6B
POLR3B	0.923	0.0152	219459_at	polymerase (RNA) III (DNA directed) polypeptide B
AARS	0.654	0.0152	201000_at	alanyl-tRNA synthetase
PNO1	1.212	0.0152	203622_s_at	partner of NOB1 homolog (S. cerevisiae)
TMPRSS3	3.508	0.0152	223949_at	transmembrane protease, serine 3
DUSP1	-2.528	0.0153	201041_s_at	dual specificity phosphatase 1
EIF2S1	0.768	0.0153	201144_s_at	eukaryotic translation initiation factor 2, subunit 1 alpha, 35kDa
LOC653739	0.821	0.0153	220949_s_at	chromosome 7 open reading frame 49 /// uncharacterized LOC653739
TMEM39A	0.89	0.0154	218615_s_at	transmembrane protein 39A
TH1L	0.86	0.0154	220607_x_at	TH1-like (Drosophila)
PRKDC	0.897	0.0154	210543_s_at	protein kinase, DNA-activated, catalytic polypeptide
PRRC1	1.12	0.0154	221734_at	proline-rich coiled-coil 1
ASPM	4.173	0.0157	219918_s_at	asp (abnormal spindle) homolog, microcephaly associated (Drosophila)
SUCNR1	-2.639	0.0157	223939_at	succinate receptor 1
RAI2	-1.926	0.0157	219440_at	retinoic acid induced 2
LOC1001295C	-1.084	0.0157	228791_at	uncharacterized LOC100129502
ARHGEF1	-0.746	0.0157	203055_s_at	Rho guanine nucleotide exchange factor (GEF) 1
NUTF2	0.851	0.0157	202397_at	nuclear transport factor 2-like /// nuclear transport factor 2
C5orf42	2.206	0.0157	219381_at	chromosome 5 open reading frame 42
MOB3B	-1.812	0.0157	226844_at	MOB kinase activator 3B
FOXO3	-0.715	0.0157	224891_at	forkhead box O3
TMEM136	1.347	0.0157	238497_at	transmembrane protein 136
KLF10	-1.078	0.0158	202393_s_at	Kruppel-like factor 10
PUF60	0.819	0.0158	209899_s_at	poly-U binding splicing factor 60KDa
DARC	-3.14	0.0158	208335_s_at	Duffy blood group, chemokine receptor
DCTPP1	0.733	0.0158	218069_at	dCTP pyrophosphatase 1
MEF2C	-1.603	0.0159	209200_at	myocyte enhancer factor 2C
NQO1	2.48	0.0159	210519_s_at	NAD(P)H dehydrogenase, quinone 1
PAM	-0.919	0.0159	202336_s_at	peptidylglycine alpha-amidating monooxygenase
GINS2	2.164	0.0159	221521_s_at	GINS complex subunit 2 (Psf2 homolog)
AFAP1-AS1	3.089	0.016	223779_at	AFAP1 antisense RNA 1 (non-protein coding)
MRPL13	1.099	0.016	218049_s_at	uncharacterized LOC100506980 /// mitochondrial ribosomal protein L13
CPSF3	0.616	0.0161	225082_at	cleavage and polyadenylation specific factor 3, 73kDa
CEP112	-1.086	0.0161	213644_at	centrosomal protein 112kDa
BCL2L2	-0.765	0.0162	209311_at	BCL2-like 2
ATOH8	-1.876	0.0163	228890_at	atonal homolog 8 (Drosophila)
SMTN	-1.281	0.0163	207390_s_at	smoothelin
PSMA1	0.562	0.0163	201676_x_at	proteasome (prosome, macropain) subunit, alpha type, 1
VPS35	0.709	0.0163	222388_s_at	vacuolar protein sorting 35 homolog (S. cerevisiae)
DNAJC10	0.987	0.0163	221782_at	DnaJ (Hsp40) homolog, subfamily C, member 10
HPS5	1.256	0.0163	204544_at	Hermansky-Pudlak syndrome 5
LOC646903	2.029	0.0163	237116_at	uncharacterized LOC646903
ZNF664-FAM1	2.674	0.0164	227320_at	family with sequence similarity 101, member A /// protein FAM101A
GOLGB1	0.468	0.0165	201057_s_at	golgin B1
ZNF271	0.843	0.0165	211009_s_at	zinc finger protein 271
LMAN2	1.126	0.0165	200805_at	lectin, mannose-binding 2
FKBP2	0.874	0.0165	203391_at	FK506 binding protein 2, 13kDa
CCL3L3	-2.768	0.0166	205114_s_at	chemokine (C-C motif) ligand 3 /// chemokine (C-C motif) ligand 3-like 1 /// chemokine (C-C motif) ligand 3-like 3
PPTC7	-0.673	0.0166	225213_at	PTC7 protein phosphatase homolog (S. cerevisiae)
ARL2BP	0.812	0.0166	202092_s_at	ADP-ribosylation factor-like 2 binding protein
PEX13	0.831	0.0166	1556009_at	peroxisomal biogenesis factor 13
KIF23	3.359	0.0167	204709_s_at	kinesin family member 23
TMED9	1.087	0.0167	208757_at	transmembrane emp24 protein transport domain containing 9
NR4A1	-3.449	0.0167	202340_x_at	nuclear receptor subfamily 4, group A, member 1
NBAS	0.502	0.0167	202926_at	neuroblastoma amplified sequence
KDELR2	0.834	0.0167	200699_at	KDEL (Lys-Asp-Glu-Leu) endoplasmic reticulum protein retention receptor 2
THSD7A	-2.116	0.0167	214920_at	thrombospondin, type I, domain containing 7A
SPRY2	-1.473	0.0167	204011_at	sprouty homolog 2 (Drosophila)
EP300	-0.715	0.0167	213579_s_at	E1A binding protein p300
FOXP1	-0.624	0.0167	229844_at	forkhead box P1
EIF2AK1	0.655	0.0167	217736_s_at	eukaryotic translation initiation factor 2-alpha kinase 1

TMEM41A	0.72	0.0167	225991_at	transmembrane protein 41A
GLIS2	0.766	0.0167	223378_at	GLIS family zinc finger 2
NIF3L1	0.845	0.0167	218133_s_at	NIF3 NGG1 interacting factor 3-like 1 (S. cerevisiae)
KPNA1	1.401	0.0167	202058_s_at	karyopherin alpha 1 (importin alpha 5)
NPAS2	1.739	0.0167	39549_at	neuronal PAS domain protein 2
CCNB1	2.961	0.0167	228729_at	cyclin B1
SASH1	-1.114	0.0167	226022_at	SAM and SH3 domain containing 1
CD97	-1.549	0.0168	202910_s_at	CD97 molecule
MSH6	0.873	0.0168	202911_at	mutS homolog 6 (E. coli)
KLHL3	-1.911	0.0169	221221_s_at	kelch-like 3 (Drosophila)
C2orf29	0.57	0.0169	224695_at	chromosome 2 open reading frame 29
INPP4B	-2.113	0.0169	235046_at	inositol polyphosphate-4-phosphatase, type II, 105kDa
GAPDH	1.449	0.0169	MGAPDH/M331	glyceraldehyde-3-phosphate dehydrogenase /// glyceraldehyde-3-phosphate dehydrogenase
CENPBD1	0.886	0.0169	1552330_at	CENPB DNA-binding domains containing 1
CYR61	-2.336	0.0169	201289_at	cysteine-rich, angiogenic inducer, 61
CLEC14A	-2.125	0.0169	226244_at	C-type lectin domain family 14, member A
MRPL9	0.731	0.017	211594_s_at	mitochondrial ribosomal protein L9
PPIH	0.593	0.0171	204228_at	peptidylprolyl isomerase H (cyclophilin H)
LOC10065328	0.692	0.0171	218003_s_at	FK506 binding protein 3, 25kDa /// uncharacterized LOC100652866 /// uncharacterized LOC100653280
CBX3	0.486	0.0172	200037_s_at	chromobox homolog 3
XPOT	0.831	0.0172	212160_at	exportin, tRNA (nuclear export receptor for tRNAs)
TMED7-TICAM	0.555	0.0173	214658_at	transmembrane emp24 protein transport domain containing 7 /// TMED7-TICAM2 readthrough
FOS	-4.873	0.0173	209189_at	FBJ murine osteosarcoma viral oncogene homolog
ACE	-2.904	0.0173	227463_at	angiotensin I converting enzyme (peptidyl-dipeptidase A) 1
N6AMT1	0.439	0.0173	220311_at	N-6 adenine-specific DNA methyltransferase 1 (putative)
GTPBP5	0.653	0.0173	226854_at	GTP binding protein 5 (putative)
NHLRC3	0.793	0.0173	236953_s_at	NHL repeat containing 3
CD69	-3.09	0.0173	209795_at	CD69 molecule
SPATA7	-0.768	0.0173	219583_s_at	spermatogenesis associated 7
RNF111	0.524	0.0173	218761_at	ring finger protein 111
EMC8	0.725	0.0173	218057_x_at	ER membrane protein complex subunit 8
ABHD16A	0.341	0.0174	230221_at	abhydrolase domain containing 16A
TRMT1L	0.772	0.0174	223404_s_at	tRNA methyltransferase 1 homolog (S. cerevisiae)-like
RAB6A	0.628	0.0174	201047_x_at	RAB6A, member RAS oncogene family
SELRC1	1.316	0.0174	219420_s_at	Sel1 repeat containing 1
CARD6	-1.266	0.0175	224414_s_at	caspase recruitment domain family, member 6
FLJ42709	-1.849	0.0176	1556695_a_at	uncharacterized LOC441094
MBNL1	-0.549	0.0176	201153_s_at	muscleblind-like splicing regulator 1
LGALSL	-1.147	0.0176	226188_at	lectin, galactoside-binding-like
FUT4	-0.954	0.0176	209892_at	fucosyltransferase 4 (alpha (1,3) fucosyltransferase, myeloid-specific)
COPB1	0.331	0.0176	201358_s_at	coatomer protein complex, subunit beta 1
HEATR1	0.82	0.0176	218594_at	HEAT repeat containing 1
ENO1	1.481	0.0176	201231_s_at	enolase 1, (alpha)
NAV3	-2.154	0.0177	204823_at	neuron navigator 3
ZNF107	1.225	0.0178	205739_x_at	zinc finger protein 107
FLT1	-1.662	0.0178	222033_s_at	fms-related tyrosine kinase 1 (vascular endothelial growth factor/vascular permeability factor receptor)
ARHGAP25	-1.605	0.0178	204882_at	Rho GTPase activating protein 25
DIAPH2	-1.555	0.0178	205726_at	diaphanous homolog 2 (Drosophila)
FBXO21	1.279	0.0178	212229_s_at	F-box protein 21
LRCH2	-2.21	0.0178	227688_at	leucine-rich repeats and calponin homology (CH) domain containing 2
EIF4E3	-1.401	0.0178	225939_at	eukaryotic translation initiation factor 4E family member 3
EIF2S2	0.656	0.0178	208725_at	eukaryotic translation initiation factor 2, subunit 2 beta, 38kDa
TRIOBP	-1.082	0.0179	243690_at	TRIO and F-actin binding protein
KLF8	-2.124	0.018	230986_at	Kruppel-like factor 8
TCEAL7	-1.937	0.018	227705_at	transcription elongation factor A (SII)-like 7
SCN7A	-4.217	0.018	228504_at	sodium channel, voltage-gated, type VII, alpha subunit
GNAS-AS1	1.419	0.018	232881_at	GNAS antisense RNA 1 (non-protein coding)
NAA25	0.648	0.018	225888_at	N(alpha)-acetyltransferase 25, NatB auxiliary subunit
CGRRF1	-0.723	0.018	204605_at	cell growth regulator with ring finger domain 1
ARL1	0.887	0.0181	201657_at	ADP-ribosylation factor-like 1
NGRN	0.771	0.0182	217722_s_at	neugrin, neurite outgrowth associated
PDRG1	0.874	0.0182	225075_at	p53 and DNA-damage regulated 1
MALT1	-0.673	0.0182	210017_at	mucosa associated lymphoid tissue lymphoma translocation gene 1
TMEM248	0.643	0.0182	224688_at	transmembrane protein 248
ZMPSTE24	0.588	0.0183	202939_at	zinc metallopeptidase STE24 homolog (S. cerevisiae)
SH2D3C	-1.63	0.0184	226673_at	SH2 domain containing 3C
PCSK5	-1.479	0.0184	213652_at	proprotein convertase subtilisin/kexin type 5
ICMT	0.575	0.0184	201611_s_at	isoprenylcysteine carboxyl methyltransferase
DGUOK	0.791	0.0184	209549_s_at	deoxyguanosine kinase
GPC1	1.906	0.0184	202756_s_at	glypican 1
KIAA1109	-0.824	0.0184	212779_at	KIAA1109
EMC9	1.153	0.0184	219203_at	ER membrane protein complex subunit 9
SFXN1	1.432	0.0184	218392_x_at	sideroflexin 1
GPR183	-2.177	0.0185	205419_at	G protein-coupled receptor 183
CKAP4	1.61	0.0186	200998_s_at	cytoskeleton-associated protein 4

LOC338758	-1.301	0.0187	238893_at	uncharacterized LOC338758
DLEU2	1.047	0.0187	216870_x_at	deleted in lymphocytic leukemia 2 (non-protein coding)
CPEB4	-0.75	0.0187	224831_at	cytoplasmic polyadenylation element binding protein 4
POLR2D	0.514	0.0187	214144_at	polymerase (RNA) II (DNA directed) polypeptide D
ASUN	0.761	0.0188	221652_s_at	asunder, spermatogenesis regulator homolog (Drosphila)
JUNB	-2.48	0.0188	201473_at	jun B proto-oncogene
POU6F1	-1.091	0.0188	205878_at	POU class 6 homeobox 1
DIABLO	0.829	0.0188	219350_s_at	diablo, IAP-binding mitochondrial protein
HSPE1	1.075	0.0188	205133_s_at	heat shock 10kDa protein 1 (chaperonin 10)
CBFA2T3	-1.488	0.0189	208056_s_at	core-binding factor, runt domain, alpha subunit 2; translocated to, 3
ISY1	0.389	0.0189	223428_s_at	ISY1 splicing factor homolog (S. cerevisiae)
TBK1	0.436	0.0189	218520_at	TANK-binding kinase 1
ATXN7L1	-0.74	0.0189	227732_at	ataxin 7-like 1
MLEC	1.127	0.0189	200616_s_at	malectin
S1PR4	-0.422	0.019	206437_at	sphingosine-1-phosphate receptor 4
OSBPL1A	-1.467	0.0191	209485_s_at	oxysterol binding protein-like 1A
FAM105B	0.94	0.0191	229268_at	family with sequence similarity 105, member B
HECW2	-2.349	0.0191	232080_at	HECT, C2 and WW domain containing E3 ubiquitin protein ligase 2
PAK6	0.607	0.0191	219461_at	p21 protein (Cdc42/Rac)-activated kinase 6
MRPS16	0.777	0.0191	222499_at	uncharacterized LOC100652993 /// uncharacterized LOC100653136 /// mitochondrial ribosomal protein S16
DEPDC1	2.141	0.0191	235545_at	DEP domain containing 1
COPS7B	0.853	0.0191	225696_at	COP9 constitutive photomorphogenic homolog subunit 7B (Arabidopsis)
ALYREF	1.345	0.0193	226320_at	Aly/REF export factor
HSBP1	0.816	0.0193	200942_s_at	heat shock factor binding protein 1
SURF4	1.067	0.0195	222979_s_at	surfeit 4
CSRNP3	-1.219	0.0195	235017_s_at	cysteine-serine-rich nuclear protein 3
IL17RA	-1.02	0.0195	229101_at	interleukin 17 receptor A
PTGES3	0.535	0.0195	200627_at	uncharacterized LOC100506732 /// prostaglandin E synthase 3 (cytosolic)
MARS	0.625	0.0195	213671_s_at	methionyl-tRNA synthetase
TMEM141	1.185	0.0195	225568_at	transmembrane protein 141
CCNB2	3.159	0.0195	202705_at	cyclin B2
ADPRH	-1.554	0.0196	228042_at	ADP-ribosylarginine hydrolase
UBA5	0.702	0.0196	222579_at	ubiquitin-like modifier activating enzyme 5
HAX1	0.778	0.0196	201145_at	HCLS1 associated protein X-1
CUL5	0.529	0.0196	203531_at	cullin 5
DNAJB14	-0.641	0.0198	226399_at	DnaJ (Hsp40) homolog, subfamily B, member 14
OTUB1	0.703	0.0199	201245_s_at	OTU domain, ubiquitin aldehyde binding 1
RPRD1A	1.425	0.0199	218209_s_at	regulation of nuclear pre-mRNA domain containing 1A
SBNO1	0.656	0.0199	218737_at	strawberry notch homolog 1 (Drosophila)
MCF2L	-2.126	0.0199	212935_at	MCF.2 cell line derived transforming sequence-like
S1PR1	-1.757	0.02	204642_at	sphingosine-1-phosphate receptor 1
MAP9	-1.204	0.02	228423_at	microtubule-associated protein 9
KIAA1147	-0.623	0.02	223162_s_at	KIAA1147
ZFAND3	0.721	0.02	218020_s_at	zinc finger, AN1-type domain 3
DDX49	0.783	0.02	210811_s_at	DEAD (Asp-Glu-Ala-Asp) box polypeptide 49
ZNF92	1.371	0.02	235170_at	zinc finger protein 92
CCL15	-3.267	0.02	205392_s_at	chemokine (C-C motif) ligand 14 /// CCL14-CCL15 readthrough /// chemokine (C-C motif) ligand 15
ARHGEF15	-1.549	0.0201	205507_at	Rho guanine nucleotide exchange factor (GEF) 15
FAM98A	0.698	0.0202	212333_at	family with sequence similarity 98, member A
LHX6	-2.288	0.0203	219884_at	LIM homeobox 6
SOX11	1.971	0.0204	204914_s_at	SRY (sex determining region Y)-box 11
PSMC1	0.398	0.0204	204219_s_at	proteasome (prosome, macropain) 26S subunit, ATPase, 1
MCAM	1.709	0.0204	211042_x_at	melanoma cell adhesion molecule
RASGRP3	-1.725	0.0204	205801_s_at	RAS guanyl releasing protein 3 (calcium and DAG-regulated)
MPP7	-2.626	0.0204	238778_at	membrane protein, palmitoylated 7 (MAGUK p55 subfamily member 7)
RCC2	0.626	0.0204	224578_at	regulator of chromosome condensation 2
C4orf48	1.631	0.0204	229860_x_at	chromosome 4 open reading frame 48
MTERF	0.483	0.0205	204871_at	mitochondrial transcription termination factor
ABCB4	-1.628	0.0205	209994_s_at	ATP-binding cassette, sub-family B (MDR/TAP), member 1 /// ATP-binding cassette, sub-family B (MDR/TAP), member 4
EMG1	0.713	0.0205	209233_at	EMG1 nucleolar protein homolog (S. cerevisiae)
ARHGEF7	-0.756	0.0206	202548_s_at	Rho guanine nucleotide exchange factor (GEF) 7
POC5	0.655	0.0206	227267_at	POC5 centriolar protein homolog (Chlamydomonas)
SYTL3	-1.673	0.0207	1562255_at	synaptotagmin-like 3
TAF2	0.498	0.0207	209523_at	TAF2 RNA polymerase II, TATA box binding protein (TBP)-associated factor, 150kDa
FGR	-2.061	0.0207	208438_s_at	Gardner-Rasheed feline sarcoma viral (v-fgr) oncogene homolog
TRIM24	1.162	0.0207	204391_x_at	tripartite motif containing 24
GPX8	1.743	0.0208	227628_at	glutathione peroxidase 8 (putative)
SPOCK2	1.972	0.0208	202523_s_at	sparc/osteonectin, cwcv and kazal-like domains proteoglycan (testican) 2
KIF14	2.765	0.0208	236641_at	kinesin family member 14
TMEM47	-1.698	0.0208	209656_s_at	transmembrane protein 47
PSMD11	0.757	0.0209	208777_s_at	proteasome (prosome, macropain) 26S subunit, non-ATPase, 11
PTK2	1.658	0.0209	241453_at	uncharacterized LOC100653024 /// uncharacterized LOC100653146 /// PTK2 protein tyrosine kinase 2
BCL2	-1.144	0.0209	203685_at	B-cell CLL/lymphoma 2
EHHADH	-1.414	0.0209	205222_at	enoyl-CoA, hydratase/3-hydroxyacyl CoA dehydrogenase
SARNP	0.618	0.021	224914_s_at	SAP domain containing ribonucleoprotein

RBM34	0.71	0.021	212591_at	AT rich interactive domain 4B (RBP1-like) /// RNA binding motif protein 34
DNMT3A	0.771	0.021	222640_at	DNA (cytosine-5-)-methyltransferase 3 alpha
WDR33	0.767	0.021	222763_s_at	SFT2 domain containing 3 /// WD repeat domain 33
CSF2RB	-1.836	0.021	205159_at	colony stimulating factor 2 receptor, beta, low-affinity (granulocyte-macrophage)
TIPRL	1.058	0.021	1554351_a_at	TIP41, TOR signaling pathway regulator-like (S. cerevisiae)
ARL5A	0.657	0.0211	218150_at	ADP-ribosylation factor-like 5A
PLN	-3.377	0.0211	204940_at	phospholamban
SLC16A14	-2.441	0.0211	238029_s_at	solute carrier family 16, member 14 (monocarboxylic acid transporter 14)
LOC10050656	0.85	0.0211	224446_at	LLP homolog, long-term synaptic facilitation (Aplysia) /// protein LLP homolog
CYP46A1	-1.254	0.0211	220331_at	cytochrome P450, family 46, subfamily A, polypeptide 1
CEP85L	-0.909	0.0212	228007_at	centrosomal protein 85kDa-like
MCM6	0.721	0.0212	201930_at	minichromosome maintenance complex component 6
TMED4	0.847	0.0212	224676_at	transmembrane emp24 protein transport domain containing 4
TRIP13	4.125	0.0212	204033_at	thyroid hormone receptor interactor 13
IKBIP	1.203	0.0212	236249_at	IKBKB interacting protein
TRPV2	-2.036	0.0213	219282_s_at	transient receptor potential cation channel, subfamily V, member 2
SAMD4B	-0.981	0.0213	229871_at	sterile alpha motif domain containing 4B
CCDC149	-0.403	0.0213	226141_at	coiled-coil domain containing 149
DPH2	0.464	0.0213	204514_at	DPH2 homolog (S. cerevisiae)
PITHD1	0.495	0.0213	223124_s_at	PITH (C-terminal proteasome-interacting domain of thioredoxin-like) domain containing 1
PPP1R7	0.538	0.0213	201214_s_at	protein phosphatase 1, regulatory subunit 7
ATP5G1	0.76	0.0213	208972_s_at	ATP synthase, H+ transporting, mitochondrial Fo complex, subunit C1 (subunit 9)
YKT6	0.891	0.0213	217785_s_at	YKT6 v-SNARE homolog (S. cerevisiae)
DLG5	0.903	0.0213	201681_s_at	discs, large homolog 5 (Drosophila)
ESPL1	1.78	0.0213	38158_at	extra spindle pole bodies homolog 1 (S. cerevisiae)
SLC6A6	-1.284	0.0213	228754_at	solute carrier family 6 (neurotransmitter transporter, taurine), member 6
CCDC85B	-1.359	0.0213	204610_s_at	coiled-coil domain containing 85B
USP39	0.821	0.0213	217829_s_at	ubiquitin specific peptidase 39
MYADM	-1.375	0.0214	225673_at	myeloid-associated differentiation marker
MID1	-1.042	0.0215	203636_at	midline 1 (Opitz/BBB syndrome)
CAPN7	0.656	0.0215	203357_s_at	calpain 7
MALSU1	0.513	0.0215	226385_s_at	mitochondrial assembly of ribosomal large subunit 1
C12orf66	0.902	0.0215	235026_at	chromosome 12 open reading frame 66
TSN	1.184	0.0215	201504_s_at	translin
TFPI	-2.422	0.0217	213258_at	tissue factor pathway inhibitor (lipoprotein-associated coagulation inhibitor)
KRT6C	2.191	0.0217	214580_x_at	keratin 6A /// keratin 6B /// keratin 6C
MYH11	-3.293	0.0217	207961_x_at	myosin, heavy chain 11, smooth muscle
ZNF717	0.736	0.0217	229986_at	zinc finger protein 717-like /// zinc finger protein 717
FOSB	-5.531	0.0218	202768_at	FBJ murine osteosarcoma viral oncogene homolog B
TRNT1	0.528	0.0218	1552625_a_at	tRNA nucleotidyl transferase, CCA-adding, 1
LOC541471	1.174	0.0219	225799_at	long intergenic non-protein coding RNA 152 /// uncharacterized LOC541471
BAG6	0.595	0.0219	201255_x_at	BCL2-associated athanogene 6
ABTB2	1.048	0.0219	213497_at	ankyrin repeat and BTB (POZ) domain containing 2
SHFM1	0.831	0.022	202276_at	split hand/foot malformation (ectrodactyly) type 1
IQGAP1	0.994	0.022	200791_s_at	IQ motif containing GTPase activating protein 1
SMARCA4	1.05	0.022	213720_s_at	SWI/SNF related, matrix associated, actin dependent regulator of chromatin, subfamily a, member 4
HIST1H3E	2.211	0.022	236278_at	Histone cluster 1, H3e
PELI1	-1.13	0.022	218319_at	pellino E3 ubiquitin protein ligase 1
EDEM3	0.706	0.0221	223243_s_at	ER degradation enhancer, mannosidase alpha-like 3
AP2M1	1.03	0.0221	200613_at	adaptor-related protein complex 2, mu 1 subunit
EGFL7	-1.674	0.0221	218825_at	EGF-like-domain, multiple 7
CREB3L2	0.502	0.0221	212345_s_at	cAMP responsive element binding protein 3-like 2
SPATS2L	-1.534	0.0221	215617_at	spermatogenesis associated, serine-rich 2-like
STAT5B	-1.012	0.0221	212549_at	signal transducer and activator of transcription 5B
MINOS1	0.741	0.0221	224867_at	mitochondrial inner membrane organizing system 1
CCNO	0.635	0.0221	210021_s_at	cyclin O
SF3A1	-0.503	0.0222	201356_at	splicing factor 3a, subunit 1, 120kDa
BUB1	2.716	0.0222	209642_at	budding uninhibited by benzimidazoles 1 homolog (yeast)
DNAJC8	0.451	0.0222	205545_x_at	DnaJ (Hsp40) homolog, subfamily C, member 8
RNF187	0.725	0.0222	212155_at	ring finger protein 187
PMS2P1	0.822	0.0222	216843_x_at	postmeiotic segregation increased 2 pseudogene 1
MTFMT	1.213	0.0222	235689_at	mitochondrial methionyl-tRNA formyltransferase
NANOS1	1.752	0.0222	228523_at	nanos homolog 1 (Drosophila)
HIST1H2BI	1.781	0.0222	210387_at	histone cluster 1, H2bc /// histone cluster 1, H2be /// histone cluster 1, H2bf /// histone cluster 1, H2bg /// histone cluster 1, H2bi
SLC2A1	3.128	0.0222	201250_s_at	solute carrier family 2 (facilitated glucose transporter), member 1
IL13RA1	-0.899	0.0222	201887_at	interleukin 13 receptor, alpha 1
SLC2A12	-1.424	0.0222	244353_s_at	solute carrier family 2 (facilitated glucose transporter), member 12
KIF2C	2.431	0.0222	209408_at	kinesin family member 2C
PRDM5	-1.639	0.0223	235764_at	PR domain containing 5
CYTIP	-1.825	0.0223	209606_at	cytohesin 1 interacting protein
ARHGDIB	-1.301	0.0223	201288_at	Rho GDP dissociation inhibitor (GDI) beta
METTL21A	1.3	0.0223	235177_at	methyltransferase like 21A
EPT1	1.194	0.0223	224888_at	ethanolaminephosphotransferase 1 (CDP-ethanolamine-specific)
FLI1	-1.817	0.0224	204236_at	Friend leukemia virus integration 1
PGBD1	-1.107	0.0224	235411_at	piggyBac transposable element derived 1

METTL13	0.46	0.0224	212405_s_at	methyltransferase like 13
LRRFIP1	-1.161	0.0224	223492_s_at	leucine rich repeat (in FLII) interacting protein 1
TWF1	0.528	0.0225	201745_at	twinfilin, actin-binding protein, homolog 1 (Drosophila)
PRKCA	1.846	0.0225	215195_at	protein kinase C, alpha
FAIM3	-2.405	0.0225	221601_s_at	Fas apoptotic inhibitory molecule 3
HCFC2	-0.603	0.0226	219484_at	host cell factor C2
DAG1	0.668	0.0226	205417_s_at	dystroglycan 1 (dystrophin-associated glycoprotein 1)
IL3RA	-1.28	0.0227	206148_at	interleukin 3 receptor, alpha (low affinity)
SLC52A2	1.146	0.0227	222155_s_at	solute carrier family 52, riboflavin transporter, member 2
RAD21	1.612	0.0227	200607_s_at	RAD21 homolog (S. pombe)
RNGTT	0.63	0.0227	204208_at	RNA guanylyltransferase and 5'-phosphatase
NR2C2AP	1.128	0.0227	226839_at	nuclear receptor 2C2-associated protein
TMEM99	1.139	0.0227	226565_at	transmembrane protein 99
Sep-15	0.495	0.0227	200902_at	15 kDa selenoprotein
NUCB2	1.977	0.0228	229838_at	nucleobindin 2
UTP18	0.637	0.0229	203721_s_at	UTP18 small subunit (SSU) processome component homolog (yeast)
TIMMDC1	0.682	0.0229	223004_s_at	translocase of inner mitochondrial membrane domain containing 1
MAMDC2	-2.961	0.023	228885_at	MAM domain containing 2
RHBDD1	0.629	0.0231	226945_at	rhomboid domain containing 1
C16orf45	-1.478	0.0231	212736_at	chromosome 16 open reading frame 45
PET100	0.485	0.0231	226006_at	PET100 homolog (S. cerevisiae)
C14orf149	1.19	0.0231	227699_at	chromosome 14 open reading frame 149
YPEL2	-1.016	0.0231	227020_at	yippee-like 2 (Drosophila)
SHQ1	0.593	0.0231	63009_at	SHQ1 homolog (S. cerevisiae)
CHCHD3	1.089	0.0231	217972_at	coiled-coil-helix-coiled-coil-helix domain containing 3
TRIP10	-1.373	0.0232	202734_at	thyroid hormone receptor interactor 10
PIM3	-1.06	0.0232	224739_at	pim-3 oncogene
TRIM8	-0.869	0.0232	221012_s_at	tripartite motif containing 8
C17orf48	-0.67	0.0234	223401_at	chromosome 17 open reading frame 48
ELAVL1	0.708	0.0234	201726_at	ELAV (embryonic lethal, abnormal vision, Drosophila)-like 1 (Hu antigen R)
CUX1	1.439	0.0234	227069_at	cut-like homeobox 1
RDH16	2.827	0.0234	206753_at	retinol dehydrogenase 16 (all-trans)
EMP1	-0.796	0.0236	201324_at	epithelial membrane protein 1
NR3C2	-2.334	0.0237	205259_at	nuclear receptor subfamily 3, group C, member 2
MARK1	-2.135	0.0237	226653_at	MAP/microtubule affinity-regulating kinase 1
HSD11B1	-1.696	0.0237	205404_at	hydroxysteroid (11-beta) dehydrogenase 1
HSD17B11	-1.277	0.0237	217989_at	hydroxysteroid (17-beta) dehydrogenase 11
TRAPPC11	-0.507	0.0237	218179_s_at	trafficking protein particle complex 11
STK32C	0.422	0.0237	230934_at	uncharacterized LOC100506057 /// serine/threonine kinase 32C
ZNF775	0.425	0.0237	228226_s_at	zinc finger protein 775
DERL1	0.583	0.0237	222543_at	derlin 1
WBSCR16	0.788	0.0237	221247_s_at	RCC1-like G exchanging factor-like /// Williams-Beuren syndrome chromosome region 16
CBX1	0.813	0.0237	201518_at	chromobox homolog 1
TSSC1	1.077	0.0237	217968_at	tumor suppressing subtransferable candidate 1
UBXN4	1.448	0.0237	212008_at	UBX domain protein 4
MPZL1	2.026	0.0237	210087_s_at	myelin protein zero-like 1
C2orf40	-3.47	0.0237	223623_at	chromosome 2 open reading frame 40
BMPR1B	-2.218	0.0237	229975_at	bone morphogenetic protein receptor, type IB
GLRB	-2.035	0.0237	205280_at	glycine receptor, beta
ABCB1	-1.914	0.0237	209993_at	ATP-binding cassette, sub-family B (MDR/TAP), member 1
MLLT3	-0.875	0.0237	204917_s_at	myeloid/lymphoid or mixed-lineage leukemia (trithorax homolog, Drosophila); translocated to, 3
RINT1	0.575	0.0237	218598_at	RAD50 interactor 1
RASD1	-2.957	0.0237	223467_at	RAS, dexamethasone-induced 1
RBBP7	0.469	0.0237	201092_at	retinoblastoma binding protein 7
ZNF322P1	0.866	0.0237	219376_at	zinc finger protein 322 /// zinc finger protein 322 pseudogene 1
C14orf28	-0.86	0.0238	238647_at	chromosome 14 open reading frame 28
SP1	0.487	0.0238	224754_at	Sp1 transcription factor
ZNF675	0.585	0.0238	217547_x_at	zinc finger protein 675
MCOLN3	-3.082	0.0238	229797_at	mucolipin 3
GALNTL2	-3.142	0.0239	239461_at	UDP-N-acetyl-alpha-D-galactosamine:polypeptide N-acetylgalactosaminyltransferase-like 2
NAMPT	-2.774	0.0239	243296_at	Nicotinamide phosphoribosyltransferase
HSPG2	-1.497	0.0239	201655_s_at	heparan sulfate proteoglycan 2
TMEM101	0.553	0.0239	225004_at	transmembrane protein 101
RB1	0.644	0.0239	203132_at	retinoblastoma 1
HNRNPA3P1	0.662	0.0239	211931_s_at	heterogeneous nuclear ribonucleoprotein A3 /// heterogeneous nuclear ribonucleoprotein A3 pseudogene 1
ANKIB1	0.606	0.0239	224687_at	ankyrin repeat and IBR domain containing 1
AOC3	-2.933	0.0239	204894_s_at	amine oxidase, copper containing 3 (vascular adhesion protein 1)
TIE1	-2.226	0.0239	204468_s_at	tyrosine kinase with immunoglobulin-like and EGF-like domains 1
PSMA4	0.555	0.024	203396_at	proteasome (prosome, macropain) subunit, alpha type, 4
SMG5	0.614	0.024	34868_at	smg-5 homolog, nonsense mediated mRNA decay factor (C. elegans)
EZH2	1.869	0.024	203358_s_at	enhancer of zeste homolog 2 (Drosophila)
SPRY4	-1.51	0.024	221489_s_at	sprouty homolog 4 (Drosophila)
PSMD4	0.554	0.0241	200882_s_at	proteasome (prosome, macropain) 26S subunit, non-ATPase, 4
LSAMP	-2.827	0.0241	235494_at	limbic system-associated membrane protein
SLC35E1	0.557	0.0241	79005_at	solute carrier family 35, member E1

PRDX1	0.731	0.0242	208680_at	peroxiredoxin 1
SLC19A2	-1.845	0.0242	209681_at	solute carrier family 19 (thiamine transporter), member 2
ISG20L2	0.582	0.0243	212766_s_at	interferon stimulated exonuclease gene 20kDa-like 2
PON3	-2.842	0.0243	213695_at	paraoxonase 3
SDF2	0.815	0.0244	203090_at	stromal cell-derived factor 2
LYRM4	0.652	0.0245	218561_s_at	LYR motif containing 4
BUB3	0.866	0.0245	201457_x_at	budding uninhibited by benzimidazoles 3 homolog (yeast)
IKZF2	-1.122	0.0245	231929_at	IKAROS family zinc finger 2 (Helios)
CTDSP2	-0.357	0.0245	203445_s_at	CTD (carboxy-terminal domain, RNA polymerase II, polypeptide A) small phosphatase 2
RNASEH2A	2.027	0.0245	203022_at	ribonuclease H2, subunit A
RAPGEF3	-1.438	0.0246	210051_at	Rap guanine nucleotide exchange factor (GEF) 3
TSPAN12	-2.352	0.0247	219274_at	tetraspanin 12
PSMD14	1.019	0.0247	212296_at	proteasome (prosome, macropain) 26S subunit, non-ATPase, 14
SMC4	1.251	0.0247	201664_at	structural maintenance of chromosomes 4
ZC3H15	0.507	0.0249	201593_s_at	zinc finger CCCH-type containing 15
PDCD6	0.977	0.0249	222380_s_at	Programmed cell death 6
DNA2	1.391	0.0249	213647_at	DNA replication helicase 2 homolog (yeast)
UBE2T	2.016	0.025	223229_at	ubiquitin-conjugating enzyme E2T (putative)
NR4A2	-3.823	0.025	204621_s_at	nuclear receptor subfamily 4, group A, member 2
PELO	-2.257	0.025	1560359_at	Pelota homolog (Drosophila)
CAD	1.018	0.0251	202715_at	carbamoyl-phosphate synthetase 2, aspartate transcarbamylase, and dihydroorotase
DNM3	-1.617	0.0252	209839_at	dynamin 3
PRPS1	0.965	0.0252	208447_s_at	phosphoribosyl pyrophosphate synthetase 1
RHOBTB1	-1.169	0.0253	212651_at	Rho-related BTB domain containing 1
HVCN1	-1.071	0.0253	226879_at	hydrogen voltage-gated channel 1
MAPK3	-0.822	0.0253	212046_x_at	mitogen-activated protein kinase 3
ANXA2P2	0.633	0.0253	208816_x_at	annexin A2 pseudogene 2
SNX17	0.711	0.0253	200991_s_at	sorting nexin 17
TUBGCP4	0.95	0.0253	211337_s_at	tubulin, gamma complex associated protein 4
FSTL3	1.879	0.0253	203592_s_at	follistatin-like 3 (secreted glycoprotein)
LMCD1	-1.977	0.0253	218574_s_at	LIM and cysteine-rich domains 1
CDC20	2.985	0.0253	202870_s_at	cell division cycle 20 homolog (S. cerevisiae)
TTC21A	-0.509	0.0254	230997_at	tetratricopeptide repeat domain 21A
TGM2	-2.707	0.0254	201042_at	transglutaminase 2 (C polypeptide, protein-glutamine-gamma-glutamyltransferase)
FLJ11710	0.717	0.0254	220529_at	uncharacterized protein FLJ11710
ATXN1L	-0.564	0.0254	227373_at	ataxin 1-like
RBM12B	0.915	0.0255	51228_at	RNA binding motif protein 12B
MXD1	-1.047	0.0255	226275_at	MAX dimerization protein 1
CEP68	-1.321	0.0255	212675_s_at	centrosomal protein 68kDa
TCF3	0.645	0.0255	210776_x_at	transcription factor 3 (E2A immunoglobulin enhancer binding factors E12/E47)
USP32	0.87	0.0255	211702_s_at	ubiquitin specific peptidase 32
TTLL12	0.904	0.0255	216251_s_at	tubulin tyrosine ligase-like family, member 12
MRPS14	0.965	0.0255	203800_s_at	mitochondrial ribosomal protein S14
NAA15	1.034	0.0255	222837_s_at	N(alpha)-acetyltransferase 15, NatA auxiliary subunit
PSME3	1.042	0.0255	200987_x_at	proteasome (prosome, macropain) activator subunit 3 (PA28 gamma; Ki)
PPP2R2C	1.214	0.0255	223574_x_at	protein phosphatase 2, regulatory subunit B, gamma
ADCK2	1.471	0.0255	221893_s_at	aarF domain containing kinase 2
NINJ2	-1.923	0.0255	219594_at	ninjurin 2
GALT	-1.186	0.0255	203179_at	galactose-1-phosphate uridylyltransferase
LOC254057	-2.415	0.0255	232370_at	uncharacterized LOC254057
GSTM5	-2.09	0.0256	205752_s_at	glutathione S-transferase mu 5
LMO4	-1.696	0.0256	229537_at	LIM domain only 4
TET3	0.367	0.0256	214754_at	tet methylcytosine dioxygenase 3
RPL8	0.852	0.0257	200936_at	ribosomal protein L8
GPR37	3.061	0.0257	209631_s_at	G protein-coupled receptor 37 (endothelin receptor type B-like)
GPR56	-1.731	0.0257	212070_at	G protein-coupled receptor 56
HNRNPL	-0.63	0.0257	221860_at	heterogeneous nuclear ribonucleoprotein L
NMT2	-1.28	0.0258	205006_s_at	N-myristoyltransferase 2
ELL3	1.871	0.0258	219518_s_at	elongation factor RNA polymerase II-like 3
PAPSS1	0.763	0.0259	209043_at	3'-phosphoadenosine 5'-phosphosulfate synthase 1
BRCA2	1.303	0.0259	208368_s_at	breast cancer 2, early onset
CBX5	1.549	0.0259	226085_at	chromobox homolog 5
CDS2	-0.633	0.0259	228456_s_at	CDP-diacylglycerol synthase (phosphatidate cytidylyltransferase) 2
DDB1	0.418	0.0259	208619_at	damage-specific DNA binding protein 1, 127kDa
KDM1A	0.629	0.0259	212348_s_at	lysine (K)-specific demethylase 1A
SLC40A1	-1.231	0.026	223044_at	solute carrier family 40 (iron-regulated transporter), member 1
MRPS34	1.104	0.026	218112_at	mitochondrial ribosomal protein S34
VTN	3.557	0.026	204534_at	vitronectin
GOLT1B	0.681	0.0261	222552_at	golgi transport 1B
MBIP	-1.652	0.0261	218411_s_at	MAP3K12 binding inhibitory protein 1
HIF1AN	0.42	0.0262	59999_at	hypoxia inducible factor 1, alpha subunit inhibitor
NEGR1	-2.995	0.0262	229461_x_at	neuronal growth regulator 1
AP4E1	0.697	0.0262	228164_at	adaptor-related protein complex 4, epsilon 1 subunit
C1orf31	0.778	0.0262	225638_at	chromosome 1 open reading frame 31
TIMM17B	1.099	0.0262	203342_at	translocase of inner mitochondrial membrane 17 homolog B (yeast)

AJUBA	1.106	0.0262	1553764_a_at	ajuba LIM protein
ORAOV1	1.333	0.0262	243531_at	oral cancer overexpressed 1
ALS2	-0.876	0.0262	232184_at	amyotrophic lateral sclerosis 2 (juvenile)
PTPRM	-1.676	0.0263	1555579_s_at	protein tyrosine phosphatase, receptor type, M
LOC10050715	1.16	0.0263	240908_at	uncharacterized LOC100507153
TET2	-0.685	0.0263	227624_at	tet methylcytosine dioxygenase 2
FOXN3	-0.875	0.0264	205022_s_at	forkhead box N3
USP14	0.459	0.0264	226567_at	ubiquitin specific peptidase 14 (tRNA-guanine transglycosylase)
LRRN3	-2.529	0.0264	209841_s_at	leucine rich repeat neuronal 3
APCDD1	-1.845	0.0265	225016_at	adenomatosis polyposis coli down-regulated 1
PLEKHA5	-1.264	0.0265	220952_s_at	pleckstrin homology domain containing, family A member 5
VDAC2	1.022	0.0265	211662_s_at	voltage-dependent anion channel 2
LOC730102	-1.338	0.0265	241607_at	quinone oxidoreductase-like protein 2 pseudogene
NAB1	0.73	0.0266	209272_at	NGFI-A binding protein 1 (EGR1 binding protein 1)
C11orf84	0.202	0.0266	227292_at	chromosome 11 open reading frame 84
SYAP1	1.057	0.0266	225154_at	synapse associated protein 1
SETBP1	-1.439	0.0266	205933_at	SET binding protein 1
YWHAZ	0.534	0.0266	200639_s_at	tyrosine 3-monooxygenase/tryptophan 5-monooxygenase activation protein, zeta polypeptide
FTSJ2	0.703	0.0266	222130_s_at	FtsJ RNA methyltransferase homolog 2 (E. coli)
FGF18	1.75	0.0266	211485_s_at	fibroblast growth factor 18
VRK2	0.695	0.0267	205126_at	vaccinia related kinase 2
CENPO	1.389	0.0267	226118_at	centromere protein O
RPL13P5	-0.498	0.0268	210822_at	ribosomal protein L13 pseudogene 5
LOC10009363	0.435	0.0269	201065_s_at	general transcription factor IIi /// general transcription factor IIi, pseudogene 1 /// general transcription factor II, i, pseudogene
IRAK1	0.958	0.0269	201587_s_at	interleukin-1 receptor-associated kinase 1
TPSAB1	-2.247	0.0269	205683_x_at	tryptase alpha/beta 1
SKP2	1.729	0.0269	203625_x_at	S-phase kinase-associated protein 2, E3 ubiquitin protein ligase
LOC10013493	-0.926	0.0269	229685_at	uncharacterized LOC100134937
PRKX	1.426	0.0269	204061_at	protein kinase, X-linked
ACP1	1.208	0.027	215227_x_at	acid phosphatase 1, soluble
RDBP	0.585	0.0271	209219_at	microRNA 1236 /// RD RNA binding protein
CLDN5	-2.208	0.0274	204482_at	claudin 5
AGBL2	1.596	0.0275	220390_at	ATP/GTP binding protein-like 2
PTBP1	0.472	0.0275	212016_s_at	polypyrimidine tract binding protein 1
MS4A2	-1.845	0.0275	207496_at	membrane-spanning 4-domains, subfamily A, member 2
HYI	1.42	0.0275	223622_s_at	hydroxypyruvate isomerase (putative)
TMOD2	-1.236	0.0276	226186_at	tropomodulin 2 (neuronal)
ERGIC2	0.524	0.0276	218135_at	ERGIC and golgi 2
RABGAP1L	-0.937	0.0276	213982_s_at	RAB GTPase activating protein 1-like
MCM7	0.888	0.0277	208795_s_at	minichromosome maintenance complex component 7
SNRPD1	0.812	0.0277	202690_s_at	small nuclear ribonucleoprotein D1 polypeptide 16kDa
EMC7	0.682	0.0278	217898_at	ER membrane protein complex subunit 7
SETD7	-1.172	0.0278	224928_at	SET domain containing (lysine methyltransferase) 7
EARS2	0.612	0.0278	227374_at	glutamyl-tRNA synthetase 2, mitochondrial (putative)
PRMT2	0.675	0.0278	228725_x_at	protein arginine methyltransferase 2
SCARA3	1.837	0.0278	219416_at	scavenger receptor class A, member 3
GPR143	2.921	0.0278	206696_at	G protein-coupled receptor 143
CENPA	3.001	0.0278	204962_s_at	chromosome 2 open reading frame 18 /// centromere protein A
ID3	-1.785	0.0279	207826_s_at	inhibitor of DNA binding 3, dominant negative helix-loop-helix protein
KIF13B	-0.699	0.0279	202962_at	kinesin family member 13B
AKAP8L	0.143	0.0279	215741_x_at	A kinase (PRKA) anchor protein 8-like
SH2B3	-1.239	0.028	203320_at	SH2B adaptor protein 3
SLC8A1	-1.217	0.0281	235518_at	solute carrier family 8 (sodium/calcium exchanger), member 1
EIF1	-0.963	0.0281	228967_at	eukaryotic translation initiation factor 1
LOC10065333	0.517	0.0281	226976_at	karyopherin alpha 6 (importin alpha 7) /// uncharacterized LOC100652828 /// uncharacterized LOC100653335
ZMYND8	1.091	0.0281	230533_at	zinc finger, MYND-type containing 8
C1orf21	-0.877	0.0281	221272_s_at	chromosome 1 open reading frame 21
SESN3	-0.891	0.0281	225123_at	sestrin 3
FAM92A1	-0.512	0.0281	228011_at	family with sequence similarity 92, member A1
ZNF254	0.517	0.0283	242602_x_at	zinc finger protein 254
API5	0.903	0.0284	214959_s_at	apoptosis inhibitor 5
TMEM208	1.123	0.0284	221597_s_at	transmembrane protein 208
LSM10	0.441	0.0285	225593_at	LSM10, U7 small nuclear RNA associated
GPHN	-1.792	0.0285	223319_at	gephyrin
RAP1GAP2	1.553	0.0285	213280_at	RAP1 GTPase activating protein 2
DCUN1D1	0.43	0.0285	218583_s_at	DCN1, defective in cullin neddylation 1, domain containing 1 (S. cerevisiae)
POLDIP2	1.074	0.0285	222425_s_at	polymerase (DNA-directed), delta interacting protein 2
ELOF1	1.105	0.0288	225156_at	elongation factor 1 homolog (S. cerevisiae)
CSNK1A1	0.327	0.0288	208867_s_at	casein kinase 1, alpha 1
CARD10	-1.589	0.0288	210026_s_at	caspase recruitment domain family, member 10
BCL6B	-2.035	0.0289	228311_at	B-cell CLL/lymphoma 6, member B
RASSF9	-1.791	0.0289	210335_at	Ras association (RalGDS/AF-6) domain family (N-terminal) member 9
GNL2	0.678	0.0289	201948_at	guanine nucleotide binding protein-like 2 (nucleolar)
ZBTB41	0.813	0.029	226962_at	zinc finger and BTB domain containing 41
PPP2R5E	0.455	0.029	203338_at	protein phosphatase 2, regulatory subunit B', epsilon isoform

CDADC1	-0.725	0.0291	221015_s_at	cytidine and dCMP deaminase domain containing 1
NARG2	0.611	0.0291	235189_at	NMDA receptor regulated 2
ERP29	0.674	0.0291	201216_at	endoplasmic reticulum protein 29
LOC730268	0.722	0.0291	231973_s_at	anaphase promoting complex subunit 1 /// anaphase-promoting complex subunit 1-like /// anaphase promoting complex subunit 1 pseudogene /// anaphase-promoting complex subunit 1-like
C1orf226	0.986	0.0291	227019_at	chromosome 1 open reading frame 226
SLC18A2	-2.48	0.0291	205857_at	solute carrier family 18 (vesicular monoamine), member 2
PCDH18	-1.671	0.0291	225975_at	protocadherin 18
VAV2	0.613	0.0291	226063_at	vav 2 guanine nucleotide exchange factor
SLC35B2	0.91	0.0291	224716_at	microRNA 4647 /// solute carrier family 35, member B2
C7orf63	-0.728	0.0291	1554919_s_at	chromosome 7 open reading frame 63
LPPR4	-2.446	0.0292	213496_at	lipid phosphate phosphatase-related protein type 4
MTAP	-1.187	0.0292	204956_at	methylthioadenosine phosphorylase
MFF	0.491	0.0292	219137_s_at	mitochondrial fission factor
PTER	1.054	0.0292	218967_s_at	phosphotriesterase related
LOC339524	-2.778	0.0292	215039_at	heparan sulfate 2-O-sulfotransferase 1 /// uncharacterized LOC339524
CNKSR3	-1.579	0.0292	227481_at	CNKSR family member 3
UBAC2	0.956	0.0292	224298_s_at	UBA domain containing 2
GIMAP6	-2.016	0.0293	229367_s_at	GTPase, IMAP family member 6
C12orf23	0.602	0.0293	224759_s_at	chromosome 12 open reading frame 23
LRRC59	1.22	0.0293	222231_s_at	leucine rich repeat containing 59
RBPJ	-0.698	0.0293	207785_s_at	recombination signal binding protein for immunoglobulin kappa J region
ZFAND2B	-0.41	0.0293	226168_at	zinc finger, AN1-type domain 2B
MRRF	0.567	0.0293	225126_at	mitochondrial ribosome recycling factor
SNF8	0.908	0.0293	218391_at	SNF8, ESCRT-II complex subunit, homolog (S. cerevisiae)
DTX4	1.281	0.0293	212611_at	deltex homolog 4 (Drosophila)
HMMR	2.811	0.0293	209709_s_at	hyaluronan-mediated motility receptor (RHAMM)
KRT5	3.423	0.0293	201820_at	keratin 5
RAP1A	-0.588	0.0294	202362_at	RAP1A, member of RAS oncogene family
RPN2	0.807	0.0294	208689_s_at	ribophorin II
ATP6V0E1	0.781	0.0294	200096_s_at	ATPase, H+ transporting, lysosomal 9kDa, V0 subunit e1
SLC35E3	0.491	0.0294	218988_at	solute carrier family 35, member E3
PDZRN4	-2.775	0.0295	220595_at	PDZ domain containing ring finger 4
ITGA1	-1.883	0.0295	226731_at	integrin, alpha 1
NSUN3	0.678	0.0295	219458_s_at	NOP2/Sun domain family, member 3
TRIM26	0.965	0.0295	202702_at	tripartite motif containing 26
RNF5	1.031	0.0295	209111_at	ring finger protein 5, E3 ubiquitin protein ligase
TMEM55A	-1.156	0.0295	226338_at	transmembrane protein 55A
TIPARP	-1.378	0.0296	212665_at	TCDD-inducible poly(ADP-ribose) polymerase
ANKRD50	-0.98	0.0296	225731_at	ankyrin repeat domain 50
EPN2	-0.905	0.0296	203464_s_at	epsin 2
RNASEH1	1.003	0.0297	218497_s_at	ribonuclease H1
CAMKK1	-0.873	0.0297	223460_at	calcium/calmodulin-dependent protein kinase kinase 1, alpha
C17orf85	-0.756	0.0297	229342_at	chromosome 17 open reading frame 85
TSSK2	-0.581	0.0297	32032_at	DiGeorge syndrome critical region gene 14 /// testis-specific serine kinase 2
PTCD2	0.764	0.0298	219658_at	pentatricopeptide repeat domain 2
FAR2	-1.91	0.0298	220615_s_at	fatty acyl CoA reductase 2
ELK3	-1.286	0.0298	221773_at	ELK3, ETS-domain protein (SRF accessory protein 2)
ANXA2P3	0.584	0.0298	211241_at	annexin A2 pseudogene 3
BUD31	0.762	0.0298	205690_s_at	BUD31 homolog (S. cerevisiae)
PFDN2	0.772	0.0299	218336_at	prefoldin subunit 2
TEX261	0.649	0.0299	212084_at	testis expressed 261
MCTS1	0.824	0.0299	218163_at	malignant T cell amplified sequence 1
PIK3R2	0.692	0.03	1568629_s_at	phosphoinositide-3-kinase, regulatory subunit 2 (beta)
FBXO11	0.995	0.03	219208_at	F-box protein 11
NDC80	2.003	0.03	204162_at	NDC80 kinetochore complex component homolog (S. cerevisiae)
SLC3A2	0.734	0.0301	200924_s_at	solute carrier family 3 (activators of dibasic and neutral amino acid transport), member 2
MCFD2	0.704	0.0301	212245_at	multiple coagulation factor deficiency 2
LOC10050945	-3.249	0.0301	236203_at	major histocompatibility complex, class II, DQ alpha 1 /// HLA class II histocompatibility antigen, DQ alpha 1 chain-like /// HLA class II histocompatibility antigen, DQ alpha 1 chain-like
PPID	-1.609	0.0302	228469_at	Peptidylprolyl isomerase D
CRY2	-1.209	0.0302	212695_at	cryptochrome 2 (photolyase-like)
SSNA1	0.748	0.0302	210378_s_at	Sjogren syndrome nuclear autoantigen 1
CCNY	0.91	0.0302	224652_at	cyclin Y
C19orf42	0.948	0.0302	229650_s_at	chromosome 19 open reading frame 42
TMEM167A	1.196	0.0302	226276_at	transmembrane protein 167A
IGFBP7	-1.152	0.0302	201162_at	insulin-like growth factor binding protein 7
USP8	0.481	0.0303	202745_at	ubiquitin specific peptidase 8
MKI67	1.308	0.0303	212023_s_at	antigen identified by monoclonal antibody Ki-67
SHCBP1	2.131	0.0303	219493_at	SHC SH2-domain binding protein 1
EIF4ENIF1	-0.738	0.0304	218626_at	eukaryotic translation initiation factor 4E nuclear import factor 1
ADAMTS13	0.667	0.0304	223844_at	ADAM metallopeptidase with thrombospondin type 1 motif, 13
DTX2P1-UPK3	0.694	0.0304	210707_x_at	DTX2P1-UPK3BP1-PMS2P11 readthrough (non-protein coding)
ASCC1	0.7	0.0304	1554627_a_at	activating signal cointegrator 1 complex subunit 1
REXO2	1.147	0.0304	223989_s_at	REX2, RNA exonuclease 2 homolog (S. cerevisiae)
RPS6KA2	-1.786	0.0305	212912_at	ribosomal protein S6 kinase, 90kDa, polypeptide 2
FAM83H	1.976	0.0306	226129_at	family with sequence similarity 83, member H

ATXN7L3B	0.477	0.0306	212114_at	ataxin 7-like 3B
KIAA1609	0.697	0.0307	65438_at	KIAA1609
TYW3	0.721	0.0307	227141_at	tRNA-yW synthesizing protein 3 homolog (S. cerevisiae)
IDE	0.723	0.0308	203327_at	insulin-degrading enzyme
CREB3	0.988	0.0308	209432_s_at	cAMP responsive element binding protein 3
GSPT1	0.786	0.0308	201912_s_at	G1 to S phase transition 1
KPNB1	0.494	0.0308	208974_x_at	karyopherin (importin) beta 1
AKR7A2	0.95	0.0308	202139_at	aldo-keto reductase family 7, member A2 (aflatoxin aldehyde reductase)
DARS	0.415	0.0309	201623_s_at	aspartyl-tRNA synthetase
ZNF521	-1.764	0.0309	226677_at	zinc finger protein 521
B3GALNT1	0.786	0.0309	211812_s_at	beta-1,3-N-acetylgalactosaminyltransferase 1 (globoside blood group)
ANAPC11	0.614	0.031	226414_s_at	anaphase promoting complex subunit 11
RABIF	0.644	0.0311	204477_at	RAB interacting factor
ANGPTL2	-1.216	0.0311	219514_at	angiopoietin-like 2
TOP2A	3.133	0.0312	201291_s_at	topoisomerase (DNA) II alpha 170kDa
TRIM29	3.735	0.0312	202504_at	tripartite motif containing 29
GSN	-1.018	0.0312	200696_s_at	gelsolin
TM2D1	0.808	0.0312	213882_at	TM2 domain containing 1
SUV39H2	1.146	0.0312	1554572_a_at	suppressor of variegation 3-9 homolog 2 (Drosophila)
YEATS4	0.884	0.0313	218911_at	YEATS domain containing 4
SLC16A9	1.406	0.0314	227506_at	solute carrier family 16, member 9 (monocarboxylic acid transporter 9)
H2AFV	0.862	0.0314	202487_s_at	H2A histone family, member V
TSEN15	0.903	0.0315	230257_s_at	tRNA splicing endonuclease 15 homolog (S. cerevisiae)
PIK3R3	-2.03	0.0317	202743_at	phosphoinositide-3-kinase, regulatory subunit 3 (gamma)
LONRF1	-1.291	0.0317	215322_at	LON peptidase N-terminal domain and ring finger 1
MECP2	-0.648	0.0317	241924_at	methyl CpG binding protein 2 (Rett syndrome)
ERI2	0.655	0.0319	213365_at	ERI1 exoribonuclease family member 2
Mar-06	1.019	0.0319	201737_s_at	membrane-associated ring finger (C3HC4) 6, E3 ubiquitin protein ligase
MAN1A1	-1.271	0.0319	221760_at	mannosidase, alpha, class 1A, member 1
POP7	0.926	0.0319	209482_at	processing of precursor 7, ribonuclease P/MRP subunit (S. cerevisiae)
MDM2	1.025	0.0319	229711_s_at	Mdm2, p53 E3 ubiquitin protein ligase homolog (mouse)
MAPKAP1	1.855	0.0319	229845_at	mitogen-activated protein kinase associated protein 1
RMI2	1.902	0.0319	226456_at	RMI2, RecQ mediated genome instability 2, homolog (S. cerevisiae)
FAM83A	3.617	0.0319	238460_at	family with sequence similarity 83, member A
ME3	-1.576	0.0319	204663_at	malic enzyme 3, NADP(+)-dependent, mitochondrial
PARG	0.32	0.0319	205060_at	poly (ADP-ribose) glycohydrolase
PTTG1IP	0.524	0.0319	200677_at	pituitary tumor-transforming 1 interacting protein
SET	0.687	0.0319	213047_x_at	SET nuclear oncogene
MYZAP	-2.557	0.0321	228568_at	GRINL1A complex locus 1 /// myocardial zonula adherens protein
CLEC1A	-1.779	0.0322	219761_at	C-type lectin domain family 1, member A
TAL1	-2.422	0.0323	206283_s_at	T-cell acute lymphocytic leukemia 1
TFDP1	0.618	0.0323	212330_at	transcription factor Dp-1
BLZF1	0.657	0.0323	32088_at	basic leucine zipper nuclear factor 1
C8orf59	0.66	0.0323	1555243_x_at	chromosome 8 open reading frame 59
HSP90B1	0.727	0.0323	216449_x_at	heat shock protein 90kDa beta (Grp94), member 1
EXOSC4	0.919	0.0323	58696_at	exosome component 4
NUSAP1	2.662	0.0323	218039_at	nucleolar and spindle associated protein 1
PPIA	0.355	0.0325	211378_x_at	peptidyl-prolyl cis-trans isomerase A-like /// peptidylprolyl isomerase A (cyclophilin A)
SLC25A27	-2.115	0.0325	1554161_at	solute carrier family 25, member 27
ANK3	-2.358	0.0325	206385_s_at	ankyrin 3, node of Ranvier (ankyrin G)
LTA4H	-1.418	0.0325	208771_s_at	leukotriene A4 hydrolase
JMY	-0.793	0.0325	241985_at	junction mediating and regulatory protein, p53 cofactor
IKZF4	-0.734	0.0325	226759_at	IKAROS family zinc finger 4 (Eos)
TUBB2B	0.602	0.0325	209372_x_at	tubulin, beta 2A class IIa /// tubulin, beta 2B class IIb
ARMC6	0.754	0.0325	221758_at	armadillo repeat containing 6
MGLL	-1.378	0.0326	225102_at	monoglyceride lipase
SNRK	-1.405	0.0326	207474_at	SNF related kinase
PARVA	1.368	0.0326	222454_s_at	parvin, alpha
RPL37	0.323	0.0327	200092_s_at	ribosomal protein L37
TYW1B	0.762	0.0327	218347_at	tRNA-yW synthesizing protein 1 homolog (S. cerevisiae) /// tRNA-yW synthesizing protein 1 homolog B (S. cerevisiae)
EDN1	-1.969	0.0327	222802_at	endothelin 1
DNAJC19	1.003	0.0327	225358_at	DnaJ (Hsp40) homolog, subfamily C, member 19
TPI1	1.011	0.0327	200822_x_at	triosephosphate isomerase 1
CHEK1	3.046	0.0327	205394_at	checkpoint kinase 1
SLC25A45	-0.957	0.0328	1563498_s_at	solute carrier family 25, member 45
STARD8	-0.945	0.0328	206868_at	StAR-related lipid transfer (START) domain containing 8
GSR	1.106	0.0331	225609_at	glutathione reductase
FIBP	0.744	0.0332	202041_s_at	fibroblast growth factor (acidic) intracellular binding protein
ACSF2	-1.741	0.0332	218844_at	acyl-CoA synthetase family member 2
CD93	-1.688	0.0334	202878_s_at	CD93 molecule
COX16	0.771	0.0334	223191_at	COX16 cytochrome c oxidase assembly homolog (S. cerevisiae)
AGTR1	-2.904	0.0335	205357_s_at	angiotensin II receptor, type 1
PDK4	-2.322	0.0335	225207_at	pyruvate dehydrogenase kinase, isozyme 4
DLEU2L	0.89	0.0335	215629_s_at	deleted in lymphocytic leukemia 2 (non-protein coding) /// deleted in lymphocytic leukemia 2-like
TCEB2	1.178	0.0335	200085_s_at	transcription elongation factor B (SIII), polypeptide 2 (18kDa, elongin B)

PSMB4	0.729	0.0335	202243_s_at	proteasome (prosome, macropain) subunit, beta type, 4
NFYC	0.662	0.0335	238231_at	Nuclear transcription factor Y, gamma
ALG1	0.274	0.0335	223355_at	asparagine-linked glycosylation 1, beta-1,4-mannosyltransferase homolog (S. cerevisiae)
C12orf32	0.754	0.0336	225837_at	chromosome 12 open reading frame 32
PCNA	0.827	0.0336	201202_at	proliferating cell nuclear antigen
CHCHD4	1.107	0.0336	229595_at	coiled-coil-helix-coiled-coil-helix domain containing 4
IL27RA	-1.884	0.0336	222062_at	interleukin 27 receptor, alpha
GALNS	0.665	0.0336	206335_at	galactosamine (N-acetyl)-6-sulfate sulfatase
DDX43	-1.5	0.0336	220004_at	DEAD (Asp-Glu-Ala-Asp) box polypeptide 43
TEAD4	1.091	0.0337	41037_at	TEA domain family member 4
MRS2	0.929	0.0337	218538_s_at	MRS2 magnesium homeostasis factor homolog (S. cerevisiae)
MKRN1	0.693	0.0337	209845_at	makorin ring finger protein 1
SRPR	0.785	0.0337	200918_s_at	signal recognition particle receptor (docking protein)
TRIM37	0.857	0.0337	213009_s_at	tripartite motif containing 37
P4HB	1.412	0.0337	200656_s_at	prolyl 4-hydroxylase, beta polypeptide
NDUFA8	0.846	0.0338	218160_at	NADH dehydrogenase (ubiquinone) 1 alpha subcomplex, 8, 19kDa
CDC23	1.28	0.0339	223651_x_at	cell division cycle 23 homolog (S. cerevisiae)
RALBP1	0.348	0.034	202845_s_at	ralA binding protein 1
NDUFA13	1.198	0.034	220864_s_at	NADH dehydrogenase (ubiquinone) 1 alpha subcomplex, 13
TMEM30B	-2.794	0.034	213285_at	transmembrane protein 30B
ANTXR2	-1.512	0.0342	228573_at	anthrax toxin receptor 2
C12orf52	0.915	0.0343	221777_at	chromosome 12 open reading frame 52
ENY2	0.798	0.0344	218482_at	enhancer of yellow 2 homolog (Drosophila)
SAR1A	1.367	0.0344	201543_s_at	SAR1 homolog A (S. cerevisiae)
DYNC1H1	0.596	0.0344	229115_at	dynein, cytoplasmic 1, heavy chain 1
UBE2C	1.738	0.0344	202954_at	ubiquitin-conjugating enzyme E2C
SURF6	0.621	0.0345	225038_s_at	surfeit 6
LOC84856	3.056	0.0345	233562_at	uncharacterized LOC84856
WISP2	-1.715	0.0345	205792_at	WNT1 inducible signaling pathway protein 2
RAPGEF2	-0.659	0.0345	203097_s_at	Rap guanine nucleotide exchange factor (GEF) 2
ADAM17	0.62	0.0346	213532_at	ADAM metallopeptidase domain 17
SIDT2	-0.725	0.0346	56256_at	SID1 transmembrane family, member 2
TMEM204	-1.416	0.0346	219315_s_at	transmembrane protein 204
POP5	0.9	0.0347	204839_at	processing of precursor 5, ribonuclease P/MRP subunit (S. cerevisiae)
NPRL3	0.598	0.0347	210672_s_at	nitrogen permease regulator-like 3 (S. cerevisiae)
PGM3	1.029	0.0347	210041_s_at	phosphoglucomutase 3
LRP8	2.188	0.0348	228955_at	low density lipoprotein receptor-related protein 8, apolipoprotein e receptor
LRRC8C	-1.823	0.0348	228314_at	leucine rich repeat containing 8 family, member C
ECT2	1.569	0.0349	219787_s_at	epithelial cell transforming sequence 2 oncogene
EFHA2	-1.589	0.035	238458_at	EF-hand domain family, member A2
NOL10	0.614	0.035	222684_s_at	nucleolar protein 10
SELE	-4.281	0.035	206211_at	selectin E
SURF2	0.217	0.035	205224_at	surfeit 2
HLF	-2.931	0.035	204755_x_at	hepatic leukemia factor
PUS7L	0.358	0.035	221025_x_at	pseudouridylate synthase 7 homolog (S. cerevisiae)-like
LRPPRC	0.792	0.035	1557360_at	leucine-rich pentatricopeptide repeat containing
SNRPE	0.821	0.035	203316_s_at	small nuclear ribonucleoprotein polypeptide E
HECTD2	-1.208	0.035	227568_at	HECT domain containing E3 ubiquitin protein ligase 2
C21orf59	0.742	0.035	218123_at	chromosome 21 open reading frame 59
GIMAP8	-1.605	0.0351	235306_at	GTPase, IMAP family member 8
GGCT	0.698	0.0351	215380_s_at	gamma-glutamylcyclotransferase
STX6	1.006	0.0351	212800_at	syntaxin 6
RGNEF	-1.142	0.0351	219610_at	190 kDa guanine nucleotide exchange factor
PZP	-1.137	0.0352	207330_at	pregnancy-zone protein
WHSC2	-0.212	0.0352	203112_s_at	microRNA 943 /// Wolf-Hirschhorn syndrome candidate 2
PPIC	0.548	0.0352	204517_at	peptidylprolyl isomerase C (cyclophilin C)
MED10	1.034	0.0352	223247_at	mediator complex subunit 10
DPT	-2.771	0.0352	213071_at	dermatopontin
POLR1C	0.783	0.0353	209317_at	polymerase (RNA) I polypeptide C, 30kDa
PDE4B	-0.858	0.0353	203708_at	phosphodiesterase 4B, cAMP-specific
AHSA1	0.532	0.0353	201491_at	AHA1, activator of heat shock 90kDa protein ATPase homolog 1 (yeast)
YARS2	0.614	0.0353	218470_at	tyrosyl-tRNA synthetase 2, mitochondrial
CTSL2	1.657	0.0353	210074_at	cathepsin L2
WDR44	0.847	0.0354	219297_at	WD repeat domain 44
CERS6	0.974	0.0354	212442_s_at	ceramide synthase 6
MBD5	0.918	0.0354	220195_at	methyl-CpG binding domain protein 5
CKS1B	1.385	0.0354	201897_s_at	CDC28 protein kinase regulatory subunit 1B
LOC10050673	0.857	0.0354	1562012_at	uncharacterized LOC100506730
TIMM44	1.082	0.0355	203093_s_at	translocase of inner mitochondrial membrane 44 homolog (yeast)
VPS25	0.659	0.0355	224608_s_at	vacuolar protein sorting 25 homolog (S. cerevisiae)
PDIA2	3.448	0.0356	206691_s_at	protein disulfide isomerase family A, member 2
SHMT2	0.817	0.0356	214096_s_at	serine hydroxymethyltransferase 2 (mitochondrial)
POLD3	0.578	0.0357	212836_at	polymerase (DNA-directed), delta 3, accessory subunit
WWC1	2.696	0.0357	216074_x_at	WW and C2 domain containing 1
BZRAP1-AS1	-1.015	0.0358	228826_at	BZRAP1 antisense RNA 1 (non-protein coding)

PNPLA4	1.384	0.0358	209739_s_at	patatin-like phospholipase domain containing 4
TSTA3	1.421	0.0359	201644_at	tissue specific transplantation antigen P35B
KIT	-2.278	0.036	205051_s_at	v-kit Hardy-Zuckerman 4 feline sarcoma viral oncogene homolog
ING3	-0.626	0.036	205070_at	inhibitor of growth family, member 3
POLR2K	0.776	0.036	202635_s_at	polymerase (RNA) II (DNA directed) polypeptide K, 7.0kDa
UROS	1.1	0.036	203031_s_at	uroporphyrinogen III synthase
PRR24	-1.037	0.0361	227325_at	proline rich 24
POLA1	0.511	0.0362	204835_at	polymerase (DNA directed), alpha 1, catalytic subunit
BAD	0.394	0.0362	232660_at	BCL2-associated agonist of cell death
NDUFB2	0.782	0.0362	218201_at	NADH dehydrogenase (ubiquinone) 1 beta subcomplex, 2, 8kDa
SNRPF	0.866	0.0362	203832_at	small nuclear ribonucleoprotein polypeptide F
TPX2	2.346	0.0362	210052_s_at	TPX2, microtubule-associated, homolog (Xenopus laevis)
ATAD3A	0.434	0.0362	219068_x_at	ATPase family, AAA domain containing 3A
TEAD2	-1.579	0.0363	226408_at	TEA domain family member 2
TMEM178A	-1.522	0.0363	229302_at	transmembrane protein 178A
PRKD1	-1.409	0.0363	205880_at	protein kinase D1
PHC1	-0.87	0.0363	225958_at	polyhomeotic homolog 1 (Drosophila)
KCNQ1OT1	0.963	0.0363	243428_at	KCNQ1 opposite strand/antisense transcript 1 (non-protein coding)
AHSA2	1.784	0.0363	230148_at	AHA1, activator of heat shock 90kDa protein ATPase homolog 2 (yeast)
SPATS2	1.016	0.0364	222593_s_at	spermatogenesis associated, serine-rich 2
STARD13	-1.167	0.0365	213103_at	StAR-related lipid transfer (START) domain containing 13
C12orf73	1.113	0.0365	226943_at	chromosome 12 open reading frame 73
PRR13	0.487	0.0367	217794_at	proline rich 13
UBE2F	0.489	0.0367	225791_at	ubiquitin-conjugating enzyme E2F (putative)
PHF20L1	0.645	0.0367	1554472_a_at	PHD finger protein 20-like 1
TOM1L2	-0.759	0.0368	226198_at	target of myb1-like 2 (chicken)
HIST1H2BD	2.866	0.0368	209911_x_at	histone cluster 1, H2bd
DUT	0.857	0.0368	208955_at	deoxyuridine triphosphatase
PLXDC1	-1.904	0.037	214081_at	plexin domain containing 1
PAIP1	1.159	0.037	208051_s_at	poly(A) binding protein interacting protein 1
PDZRN3	-1.464	0.0371	212915_at	PDZ domain containing ring finger 3
KARS	0.474	0.0371	200079_s_at	lysyl-tRNA synthetase
TMEM179B	0.587	0.0371	228089_x_at	transmembrane protein 179B
GAK	-0.593	0.0371	40225_at	cyclin G associated kinase
ENG	-1.56	0.0372	201809_s_at	endoglin
DOCK8	-0.962	0.0372	232843_s_at	dedicator of cytokinesis 8
NUP107	0.631	0.0372	218768_at	nucleoporin 107kDa
FER	0.73	0.0372	206412_at	fer (fps/fes related) tyrosine kinase
MIR3620	0.754	0.0372	208750_s_at	ADP-ribosylation factor 1 /// microRNA 3620
TGIF2-C20OR	1.244	0.0372	224376_s_at	chromosome 20 open reading frame 24 /// TGIF2-C20orf24 readthrough
SETD3	-0.399	0.0372	212465_at	SET domain containing 3
ABI2	0.908	0.0372	211793_s_at	abl-interactor 2
XPO1	0.367	0.0372	208775_at	exportin 1 (CRM1 homolog, yeast)
TBCA	0.643	0.0373	203667_at	tubulin folding cofactor A
XPO5	0.815	0.0373	223056_s_at	exportin 5
FXYD5	-1.515	0.0374	224252_s_at	FXYD domain containing ion transport regulator 5
CARD11	1.072	0.0374	223514_at	caspase recruitment domain family, member 11
C6orf62	1.364	0.0374	213875_x_at	chromosome 6 open reading frame 62
RNF34	0.497	0.0375	219035_s_at	ring finger protein 34, E3 ubiquitin protein ligase
BOP1	1.594	0.0375	212563_at	block of proliferation 1
ETS2	-1.25	0.0376	201328_at	v-ets erythroblastosis virus E26 oncogene homolog 2 (avian)
AKR7A3	0.787	0.0376	216381_x_at	aldo-keto reductase family 7, member A3 (aflatoxin aldehyde reductase)
LAMTOR2	0.623	0.0376	218291_at	late endosomal/lysosomal adaptor, MAPK and MTOR activator 2
SSX2IP	0.502	0.0376	203015_s_at	synovial sarcoma, X breakpoint 2 interacting protein
LOC10013482	-0.941	0.0377	231886_at	uncharacterized LOC100134822
FLJ43489	0.849	0.0377	244551_at	uncharacterized LOC644283
PRDX5	1.16	0.0377	1560587_s_at	peroxiredoxin 5
MYCL1	1.334	0.0377	214058_at	v-myc myelocytomatosis viral oncogene homolog 1, lung carcinoma derived (avian)
DHX9	0.564	0.0377	202420_s_at	DEAH (Asp-Glu-Ala-His) box polypeptide 9
FMO2	-3.309	0.0377	228268_at	flavin containing monooxygenase 2 (non-functional)
CISD2	0.931	0.0377	226686_at	CDGSH iron sulfur domain 2
SMAD9	-1.668	0.0377	227719_at	SMAD family member 9
CXorf69	-1.159	0.0377	227976_at	chromosome X open reading frame 69
MTM1	-0.933	0.0377	36920_at	myotubularin 1
ZC3HC1	0.52	0.0377	223163_s_at	zinc finger, C3HC-type containing 1
DCAF6	0.554	0.0377	232349_x_at	DDB1 and CUL4 associated factor 6
IRS1	-1.444	0.0377	204686_at	insulin receptor substrate 1
KIF16B	-0.695	0.0377	219570_at	kinesin family member 16B
MRPS21	0.414	0.038	222997_s_at	mitochondrial ribosomal protein S21
ICAM2	-1.52	0.038	213620_s_at	intercellular adhesion molecule 2
NFATC4	-1.353	0.0381	236270_at	nuclear factor of activated T-cells, cytoplasmic, calcineurin-dependent 4
NMNAT3	-0.884	0.0381	228090_at	nicotinamide nucleotide adenylyltransferase 3
GABRB2	-3.417	0.0382	242344_at	gamma-aminobutyric acid (GABA) A receptor, beta 2
ALG3	0.922	0.0382	207396_s_at	asparagine-linked glycosylation 3, alpha-1,3- mannosyltransferase homolog (S. cerevisiae)
SMEK2	1.002	0.0382	222270_at	SMEK homolog 2, suppressor of mek1 (Dictyostelium)

HELLS	1.603	0.0382	223556_at	helicase, lymphoid-specific
OGFOD2	0.604	0.0382	219245_s_at	2-oxoglutarate and iron-dependent oxygenase domain containing 2
PSMC3IP	0.337	0.0383	205956_x_at	PSMC3 interacting protein
DDX1	0.488	0.0383	201241_at	DEAD (Asp-Glu-Ala-Asp) box helicase 1
SELP	-2.264	0.0383	206049_at	selectin P (granule membrane protein 140kDa, antigen CD62)
MELK	2.816	0.0383	204825_at	maternal embryonic leucine zipper kinase
CLIC2	-1.701	0.0383	213415_at	chloride intracellular channel 2
NRD1	0.593	0.0383	208709_s_at	nardilysin (N-arginine dibasic convertase)
MOCOS	1.984	0.0384	219959_at	molybdenum cofactor sulfurase
C5orf34	1.448	0.0384	229886_at	chromosome 5 open reading frame 34
HSPA4	0.855	0.0384	211015_s_at	heat shock 70kDa protein 4
PODN	-1.794	0.0386	226522_at	podocan
RHOJ	-1.714	0.0386	238905_at	ras homolog family member J
PLK1	0.207	0.0386	202240_at	polo-like kinase 1
EPRS	0.495	0.0386	200842_s_at	glutamyl-prolyl-tRNA synthetase
LUC7L2	0.573	0.0386	217842_at	LUC7-like 2 (S. cerevisiae)
ZFYVE16	2.051	0.0387	1555982_at	zinc finger, FYVE domain containing 16
MED20	0.592	0.0387	212872_s_at	mediator complex subunit 20
FLJ39739	1.038	0.0387	1556033_at	uncharacterized FLJ39739
DDOST	1.132	0.0387	208675_s_at	dolichyl-diphosphooligosaccharide--protein glycosyltransferase
CUTA	0.831	0.0387	221488_s_at	cutA divalent cation tolerance homolog (E. coli)
C10orf35	1.404	0.0388	226313_at	chromosome 10 open reading frame 35
HPGDS	-2.231	0.0388	206726_at	hematopoietic prostaglandin D synthase
MPG	0.671	0.0388	203686_at	N-methylpurine-DNA glycosylase
GGH	1.398	0.0389	203560_at	gamma-glutamyl hydrolase (conjugase, folylpolygammaglutamyl hydrolase)
C1orf144	0.628	0.0389	212004_at	chromosome 1 open reading frame 144
SPC25	1.971	0.0391	209891_at	SPC25, NDC80 kinetochore complex component, homolog (S. cerevisiae)
C8orf4	-2.954	0.0391	218541_s_at	chromosome 8 open reading frame 4
CFL2	-0.998	0.0392	224352_s_at	cofilin 2 (muscle)
GCFC2	1.021	0.0392	210175_at	GC-rich sequence DNA-binding factor 2
PARK7	0.521	0.0393	200006_at	parkinson protein 7
SPIRE2	0.359	0.0394	227706_at	spire homolog 2 (Drosophila)
TMEM106C	1.195	0.0395	201764_at	transmembrane protein 106C
FOXM1	1.618	0.0395	202580_x_at	forkhead box M1
APTX	0.638	0.0397	218527_at	aprataxin
ROBO4	-2.127	0.0397	226028_at	roundabout, axon guidance receptor, homolog 4 (Drosophila)
FAT4	-1.535	0.0398	219427_at	FAT tumor suppressor homolog 4 (Drosophila)
CCBE1	-2.441	0.0399	229641_at	collagen and calcium binding EGF domains 1
SPSB1	-0.989	0.04	226075_at	splA/ryanodine receptor domain and SOCS box containing 1
OSBPL8	-0.582	0.04	212585_at	oxysterol binding protein-like 8
BEND7	-1.538	0.0401	227341_at	BEN domain containing 7
NUCKS1	0.992	0.0401	222424_s_at	nuclear casein kinase and cyclin-dependent kinase substrate 1
SEC24A	0.49	0.0401	212900_at	SEC24 family, member A (S. cerevisiae)
MMRN2	-1.751	0.0401	236262_at	multimerin 2
ARL8B	0.321	0.0401	217852_s_at	ADP-ribosylation factor-like 8B
SETDB1	0.454	0.0402	214197_s_at	SET domain, bifurcated 1
SEMA6A	-1.712	0.0403	223449_at	sema domain, transmembrane domain (TM), and cytoplasmic domain, (semaphorin) 6A
QKI	-1.121	0.0403	236154_at	QKI, KH domain containing, RNA binding
IMPAD1	0.722	0.0403	222654_at	inositol monophosphatase domain containing 1
CTSF	-1.179	0.0403	203657_s_at	cathepsin F
FBXO45	0.724	0.0403	225100_at	F-box protein 45
TSPAN7	-2.751	0.0404	202242_at	tetraspanin 7
LARP4	0.454	0.0404	212714_at	La ribonucleoprotein domain family, member 4
MED8	0.754	0.0404	213126_at	mediator complex subunit 8
FAM63B	1.26	0.0404	222111_at	family with sequence similarity 63, member B
SEMA3G	-2.478	0.0404	219689_at	sema domain, immunoglobulin domain (Ig), short basic domain, secreted, (semaphorin) 3G
PLCG2	-0.921	0.0405	204613_at	phospholipase C, gamma 2 (phosphatidylinositol-specific)
GBP2	-1.539	0.0406	242907_at	guanylate binding protein 2, interferon-inducible
NCOA1	-0.612	0.0406	209105_at	nuclear receptor coactivator 1
GATAD1	0.713	0.0406	214718_at	GATA zinc finger domain containing 1
SGCD	-1.556	0.0406	213543_at	sarcoglycan, delta (35kDa dystrophin-associated glycoprotein)
EIF3B	0.575	0.0407	211501_s_at	eukaryotic translation initiation factor 3, subunit B
CHCHD2	0.955	0.0407	217720_at	coiled-coil-helix-coiled-coil-helix domain containing 2
FAM108B1	-0.36	0.0408	227551_at	family with sequence similarity 108, member B1
C11orf80	1.321	0.0408	238593_at	chromosome 11 open reading frame 80
CH25H	-2.304	0.0408	206932_at	cholesterol 25-hydroxylase
ATP13A1	0.864	0.0408	218052_s_at	ATPase type 13A1
TXNDC15	0.271	0.041	220495_s_at	thioredoxin domain containing 15
GLRX2	0.9	0.041	219933_at	glutaredoxin 2
ZNF826P	1.13	0.041	1569191_at	zinc finger protein 826, pseudogene
MIEN1	0.784	0.0411	224447_s_at	migration and invasion enhancer 1
LOC10065297	-1.39	0.0412	225602_at	GLI pathogenesis-related 2 /// uncharacterized LOC100652973
BRD8	0.679	0.0412	242265_at	Bromodomain containing 8
CDH19	-2.057	0.0412	206898_at	cadherin 19, type 2
VPS37B	-0.943	0.0412	221704_s_at	vacuolar protein sorting 37 homolog B (S. cerevisiae)

OMA1	0.716	0.0412	226020_s_at	disabled homolog 1 (Drosophila) /// OMA1 zinc metallopeptidase homolog (S. cerevisiae)
GSK3B	0.904	0.0412	209945_s_at	glycogen synthase kinase 3 beta
MAD2L1	1.916	0.0412	203362_s_at	MAD2 mitotic arrest deficient-like 1 (yeast)
PPAP2B	-1.144	0.0412	212230_at	phosphatidic acid phosphatase type 2B
C19orf52	0.687	0.0412	231901_at	chromosome 19 open reading frame 52
TIGD6	0.601	0.0413	220986_s_at	tigger transposable element derived 6
HMBS	1.117	0.0414	203040_s_at	hydroxymethylbilane synthase
FAM13A	-1.615	0.0414	202973_x_at	family with sequence similarity 13, member A
MAP7D3	-1.263	0.0414	219626_at	MAP7 domain containing 3
PLCB4	-2.492	0.0415	203895_at	phospholipase C, beta 4
HLA-DOB	-1.034	0.0415	205671_s_at	major histocompatibility complex, class II, DO beta
PARN	0.61	0.0416	203905_at	poly(A)-specific ribonuclease
MZT2A	1.251	0.0416	213166_x_at	microRNA 4784 /// mitotic spindle organizing protein 2A
ODC1	1.179	0.0417	200790_at	ornithine decarboxylase 1
NFKBIA	-1.021	0.0417	201502_s_at	nuclear factor of kappa light polypeptide gene enhancer in B-cells inhibitor, alpha
GMCL1	0.8	0.0417	239355_at	germ cell-less homolog 1 (Drosophila)
RACGAP1	1.533	0.0417	222077_s_at	Rac GTPase activating protein 1
FNDC3B	2.29	0.0417	229865_at	fibronectin type III domain containing 3B
MRPL27	0.774	0.0417	224330_s_at	mitochondrial ribosomal protein L27
C5orf15	0.447	0.0418	203024_s_at	chromosome 5 open reading frame 15
TARDBP	0.366	0.0419	200020_at	TAR DNA binding protein
SEPP1	-0.829	0.0419	201427_s_at	selenoprotein P, plasma, 1
NFATC2	-1.637	0.042	228442_at	nuclear factor of activated T-cells, cytoplasmic, calcineurin-dependent 2
MAD2L2	0.772	0.042	223234_at	MAD2 mitotic arrest deficient-like 2 (yeast)
AHCY	1.208	0.042	200903_s_at	adenosylhomocysteinase
GADD45GIP1	0.714	0.042	212891_s_at	growth arrest and DNA-damage-inducible, gamma interacting protein 1
Mar-05	0.769	0.042	218582_at	membrane-associated ring finger (C3HC4) 5
TCEB1	0.586	0.0421	202824_s_at	transcription elongation factor B (SIII), polypeptide 1 (15kDa, elongin C)
KIF15	1.699	0.0421	219306_at	kinesin family member 15
LRFN4	0.715	0.0421	219491_at	leucine rich repeat and fibronectin type III domain containing 4
EMC1	0.594	0.0421	212395_s_at	ER membrane protein complex subunit 1
MRPL50	0.304	0.0422	225581_s_at	mitochondrial ribosomal protein L50
SGCE	-1.162	0.0423	204688_at	sarcoglycan, epsilon
NCAPD3	1.775	0.0423	212789_at	non-SMC condensin II complex, subunit D3
GTF3C1	0.654	0.0423	202320_at	general transcription factor IIIC, polypeptide 1, alpha 220kDa
GTF2I	0.969	0.0423	210892_s_at	general transcription factor IIi
ADAM10	1.348	0.0423	202604_x_at	ADAM metallopeptidase domain 10
TARS	0.949	0.0424	201263_at	threonyl-tRNA synthetase
CETN2	0.489	0.0426	209194_at	centrin, EF-hand protein, 2
PGAP3	-0.735	0.0426	221811_at	post-GPI attachment to proteins 3
LYZ	-2.321	0.0426	213975_s_at	lysozyme
EGFL6	-3.048	0.0427	219454_at	EGF-like-domain, multiple 6
CD320	0.811	0.0427	218529_at	CD320 molecule
NMRAL1	0.872	0.0427	223206_s_at	NmrA-like family domain containing 1
AASDHPPT	0.797	0.0427	202170_s_at	aminoadipate-semialdehyde dehydrogenase-phosphopantetheinyl transferase
ORC6	1.316	0.0427	219105_x_at	origin recognition complex, subunit 6
TM2D2	0.64	0.0427	224413_s_at	TM2 domain containing 2
NUMB	-0.677	0.0428	207545_s_at	numb homolog (Drosophila)
CCL18	-3.378	0.0429	32128_at	chemokine (C-C motif) ligand 18 (pulmonary and activation-regulated)
ITGA9	-1.795	0.0429	227297_at	integrin, alpha 9
C8orf33	0.976	0.0429	222551_s_at	chromosome 8 open reading frame 33
DENND1A	2.248	0.043	219763_at	DENN/MADD domain containing 1A
ZDHHC2	-1.64	0.0431	244779_at	zinc finger, DHHC-type containing 2
UNC50	0.279	0.0431	203583_at	unc-50 homolog (C. elegans)
ZMAT3	1.363	0.0432	228315_at	zinc finger, matrin-type 3
VTA1	0.729	0.0432	224437_s_at	Vps20-associated 1 homolog (S. cerevisiae)
CLUAP1	0.79	0.0432	204576_s_at	clusterin associated protein 1
TTC3P1	0.84	0.0432	1569472_s_at	tetratricopeptide repeat domain 3 /// tetratricopeptide repeat domain 3 pseudogene 1
SHB	1.058	0.0432	204656_at	Src homology 2 domain containing adaptor protein B
HJURP	1.753	0.0432	218726_at	Holliday junction recognition protein
ZNF284	-0.733	0.0433	239462_at	Zinc finger protein 284
CXCL3	-2.458	0.0433	207850_at	chemokine (C-X-C motif) ligand 3
SNRPA1	0.716	0.0433	215722_s_at	small nuclear ribonucleoprotein polypeptide A'
MTMR2	0.854	0.0433	203212_s_at	myotubularin related protein 2
LRRC8D	0.705	0.0433	218684_at	leucine rich repeat containing 8 family, member D
CENPH	0.797	0.0433	231772_x_at	centromere protein H
ZWINT	2.465	0.0436	204026_s_at	ZW10 interactor
FRMD3	-1.805	0.0437	229893_at	FERM domain containing 3
ZBED3	-0.98	0.0437	235109_at	zinc finger, BED-type containing 3
HAT1	0.622	0.0437	203138_at	histone acetyltransferase 1
ZZEF1	-0.497	0.0437	212601_at	zinc finger, ZZ-type with EF-hand domain 1
SPAG5	1.887	0.0437	203145_at	sperm associated antigen 5
RRAS2	1.391	0.0441	208456_s_at	related RAS viral (r-ras) oncogene homolog 2
CYP26A1	3.502	0.0441	206424_at	cytochrome P450, family 26, subfamily A, polypeptide 1
TCF19	1.35	0.0441	223274_at	transcription factor 19

TTK	2.506	0.0442	204822_at	TTK protein kinase
C15orf38-AP3	1.104	0.0443	202399_s_at	adaptor-related protein complex 3, sigma 2 subunit /// C15orf38-AP3S2 readthrough
LRP11	1.604	0.0443	225060_at	low density lipoprotein receptor-related protein 11
TNFRSF14	-0.794	0.0445	209354_at	tumor necrosis factor receptor superfamily, member 14
TSFM	0.714	0.0445	212656_at	Ts translation elongation factor, mitochondrial
CSTF3	0.991	0.0446	229665_at	cleavage stimulation factor, 3' pre-RNA, subunit 3, 77kDa
SGPP1	-0.525	0.0446	223391_at	sphingosine-1-phosphate phosphatase 1
OTUD6B	0.562	0.0447	222825_at	OTU domain containing 6B
UNG	0.947	0.0447	202330_s_at	uracil-DNA glycosylase
C3orf14	1.113	0.0447	219288_at	chromosome 3 open reading frame 14
C1orf54	-1.133	0.0447	219506_at	chromosome 1 open reading frame 54
CNPY3	0.773	0.0447	1552977_a_at	canopy 3 homolog (zebrafish)
SLC44A1	-0.865	0.0449	228486_at	solute carrier family 44, member 1
HNRNPAB	0.657	0.0449	201277_s_at	heterogeneous nuclear ribonucleoprotein A/B
C15orf23	0.996	0.045	225300_at	chromosome 15 open reading frame 23
SLAIN2	-0.668	0.045	224844_at	SLAIN motif family, member 2
EPB41L2	-1.436	0.045	201719_s_at	erythrocyte membrane protein band 4.1-like 2
TNPO2	0.402	0.045	226428_at	transportin 2
CTPS1	1.097	0.045	202613_at	CTP synthase 1
HSPB11	1.207	0.0451	203960_s_at	heat shock protein family B (small), member 11
MAP2	-1.676	0.0451	225540_at	microtubule-associated protein 2
MAPK8	-0.569	0.0452	229664_at	mitogen-activated protein kinase 8
IER2	-1.976	0.0452	202081_at	immediate early response 2
MIR4448	-1.148	0.0452	220692_at	microRNA 4448
PPP2R3C	-0.849	0.0452	218852_at	protein phosphatase 2, regulatory subunit B'', gamma
PDK3	-0.819	0.0452	228959_at	pyruvate dehydrogenase kinase, isozyme 3
NOS1AP	0.45	0.0452	1563512_at	Nitric oxide synthase 1 (neuronal) adaptor protein
DNAJC13	0.572	0.0452	212467_at	DnaJ (Hsp40) homolog, subfamily C, member 13
TMEM60	0.66	0.0452	223396_at	transmembrane protein 60
ZNF519	1.594	0.0454	1568873_at	zinc finger protein 519
SARS2	-1.699	0.0454	220233_at	F-box protein 17 /// seryl-tRNA synthetase 2, mitochondrial
NR2F1	-1.39	0.0454	209506_s_at	nuclear receptor subfamily 2, group F, member 1
LARP1	0.354	0.0454	212137_at	La ribonucleoprotein domain family, member 1
RNF146	-0.586	0.0456	223886_s_at	ring finger protein 146
C11orf63	-0.55	0.0456	220141_at	chromosome 11 open reading frame 63
DCAF17	0.548	0.0456	220172_at	DDB1 and CUL4 associated factor 17
LOC10050723	1.123	0.0456	228799_at	uncharacterized LOC100507239
GDPD5	1.468	0.0456	32502_at	glycerophosphodiester phosphodiesterase domain containing 5
PLEKHG5	-0.296	0.0458	227142_at	pleckstrin homology domain containing, family G (with RhoGef domain) member 5
DEPDC1B	1.944	0.0458	226980_at	DEP domain containing 1B
ARMC10	1.129	0.0458	223328_at	armadillo repeat containing 10
ERCC1	-0.929	0.0459	228131_at	excision repair cross-complementing rodent repair deficiency, complementation group 1 (includes overlapping antisense sequence)
PSMB7	0.573	0.0459	200786_at	proteasome (prosome, macropain) subunit, beta type, 7
YEATS2	0.539	0.046	221203_s_at	YEATS domain containing 2
CAMKK2	-0.734	0.046	213812_s_at	calcium/calmodulin-dependent protein kinase kinase 2, beta
HNRNPR	0.322	0.046	208766_s_at	heterogeneous nuclear ribonucleoprotein R
UBE3A	0.266	0.0461	211285_s_at	ubiquitin protein ligase E3A
STX16	0.736	0.0461	1558249_s_at	syntaxin 16
EMC4	0.684	0.0461	223043_at	ER membrane protein complex subunit 4
ND4	-0.334	0.0463	224373_s_at	NADH dehydrogenase, subunit 4 (complex I)
CCDC138	0.914	0.0463	235644_at	coiled-coil domain containing 138
PRMT5	0.774	0.0463	1564520_s_at	protein arginine methyltransferase 5
SSFA2	-1.036	0.0463	202506_at	sperm specific antigen 2
LCORL	-0.497	0.0464	232293_at	ligand dependent nuclear receptor corepressor-like
LRR1	1.083	0.0464	235113_at	leucine rich repeat protein 1
C2orf47	0.608	0.0465	219176_at	chromosome 2 open reading frame 47
Sep-08	-1.248	0.0466	226627_at	septin 8
PPFIA2	-1.323	0.0466	232073_at	protein tyrosine phosphatase, receptor type, f polypeptide (PTPRF), interacting protein (liprin), alpha 2
NPEPL1	0.389	0.0467	89476_r_at	aminopeptidase-like 1
PSMG4	0.582	0.0467	228217_s_at	proteasome (prosome, macropain) assembly chaperone 4
KREMEN1	-1.648	0.0467	227250_at	kringle containing transmembrane protein 1
SORD	0.637	0.0467	201562_s_at	sorbitol dehydrogenase
ALKBH7	1.096	0.0467	227878_s_at	alkB, alkylation repair homolog 7 (E. coli)
PHF2	-0.527	0.0467	212726_at	PHD finger protein 2
PARS2	0.444	0.0467	227348_at	prolyl-tRNA synthetase 2, mitochondrial (putative)
ARPC5	0.941	0.0467	1555797_a_at	actin related protein 2/3 complex, subunit 5, 16kDa
PMS2P3	0.872	0.0467	214473_x_at	postmeiotic segregation increased 2 pseudogene 3
NSD1	1	0.0467	235760_at	nuclear receptor binding SET domain protein 1
N4BP1	-0.837	0.0468	221867_at	NEDD4 binding protein 1
WFS1	-1.532	0.0468	202908_at	Wolfram syndrome 1 (wolframin)
SWI5	1.087	0.0469	226027_at	SWI5 recombination repair homolog (yeast)
DUSP2	-2.607	0.047	204794_at	dual specificity phosphatase 2
STMN1	0.272	0.047	217714_x_at	microRNA 3917 /// stathmin 1
GLG1	0.571	0.0471	214730_s_at	golgi glycoprotein 1
LGALS3	-1.6	0.0471	1557197_a_at	lectin, galactoside-binding, soluble, 3

MYO15B	-1.477	0.0471	219173_at	myosin XVB pseudogene
LOC642236	-0.858	0.0471	242770_at	FSHD region gene 1 pseudogene
PPP1R21	0.395	0.0471	1554145_a_at	protein phosphatase 1, regulatory subunit 21
TAX1BP3	0.484	0.0471	209154_at	P2RX5-TAX1BP3 readthrough /// Tax1 (human T-cell leukemia virus type I) binding protein 3
CLTB	1.028	0.0471	205172_x_at	clathrin, light chain B
TXNDC17	0.679	0.0473	224511_s_at	thioredoxin domain containing 17
ZNF202	0.999	0.0473	204329_s_at	zinc finger protein 202
WTH3DI	0.548	0.0474	210406_s_at	RAB6A, member RAS oncogene family /// RAB6C, member RAS oncogene family /// RAB6C-like
TRAPPC4	0.854	0.0474	217959_s_at	microRNA 3656 /// trafficking protein particle complex 4
CAV1	-1.437	0.0474	212097_at	caveolin 1, caveolae protein, 22kDa
POC1A	0.481	0.0474	226355_at	POC1 centriolar protein homolog A (Chlamydomonas)
LRRC42	1.196	0.0474	215084_s_at	leucine rich repeat containing 42
ABCB6	1.328	0.0474	203192_at	ATP-binding cassette, sub-family B (MDR/TAP), member 6
FBLN5	-1.727	0.0474	203088_at	fibulin 5
WDR61	0.255	0.0475	232075_at	WD repeat domain 61
ATAD2	0.807	0.0475	228401_at	ATPase family, AAA domain containing 2
TRIM2	-0.8	0.0475	202342_s_at	tripartite motif containing 2
ZBTB8OS	0.611	0.0475	228970_at	zinc finger and BTB domain containing 8 opposite strand
MYL12B	0.37	0.0476	221474_at	myosin, light chain 12B, regulatory
DPP8	0.423	0.0476	220939_s_at	dipeptidyl-peptidase 8
HEATR6	0.52	0.0476	218991_at	HEAT repeat containing 6
DPY30	0.697	0.0476	224129_s_at	dpy-30 homolog (C. elegans)
WDR74	0.918	0.0476	221712_s_at	WD repeat domain 74
CDK1	2.372	0.0476	210559_s_at	cyclin-dependent kinase 1
CDC42EP5	-1.27	0.0476	227850_x_at	CDC42 effector protein (Rho GTPase binding) 5
NUDT6	-1.191	0.0476	220183_s_at	nudix (nucleoside diphosphate linked moiety X)-type motif 6
CSRNP1	-2.202	0.0477	225557_at	cysteine-serine-rich nuclear protein 1
WDR46	0.738	0.0478	209196_at	WD repeat domain 46
GUCY1B3	-1.642	0.0478	203817_at	guanylate cyclase 1, soluble, beta 3
MSL3	1.036	0.0479	236165_at	male-specific lethal 3 homolog (Drosophila)
TFDP2	0.795	0.048	203589_s_at	transcription factor Dp-2 (E2F dimerization partner 2)
TROAP	0.89	0.048	204649_at	trophinin associated protein (tastin)
PRTG	-0.99	0.048	229073_at	protogenin
ELF2	-0.393	0.048	242735_x_at	E74-like factor 2 (ets domain transcription factor)
SOX17	-2.195	0.048	230943_at	SRY (sex determining region Y)-box 17
RRN3P1	-1.848	0.048	215211_at	RNA polymerase I transcription factor homolog (S. cerevisiae) pseudogene 1
TSHZ2	-2.24	0.0481	238577_s_at	teashirt zinc finger homeobox 2
LOC10050659	0.759	0.0481	1559026_at	uncharacterized LOC100506599
TUFM	0.826	0.0481	201113_at	Tu translation elongation factor, mitochondrial
ACTR2	0.425	0.0482	200728_at	ARP2 actin-related protein 2 homolog (yeast)
SIRT2	-1.42	0.0483	1558331_at	sirtuin 2
SP140L	0.732	0.0483	223934_at	SP140 nuclear body protein-like
C19orf10	0.946	0.0483	216483_s_at	chromosome 19 open reading frame 10
THAP5	0.694	0.0484	227636_at	THAP domain containing 5
ARAP3	-1.652	0.0484	218950_at	ArfGAP with RhoGAP domain, ankyrin repeat and PH domain 3
SSR2	0.708	0.0484	200652_at	signal sequence receptor, beta (translocon-associated protein beta)
NMU	3.451	0.0484	206023_at	neuromedin U
UNC45A	0.596	0.0484	221708_s_at	unc-45 homolog A (C. elegans)
WWTR1	1.055	0.0484	202134_s_at	WW domain containing transcription regulator 1
CD52	-2.217	0.0485	34210_at	CD52 molecule
VSIG2	-2.145	0.0485	229369_at	V-set and immunoglobulin domain containing 2
S1PR3	-1.491	0.0485	228176_at	sphingosine-1-phosphate receptor 3
ANAPC7	0.451	0.0485	225554_s_at	anaphase promoting complex subunit 7
CLNS1A	0.941	0.0485	209143_s_at	chloride channel, nucleotide-sensitive, 1A
KRT6A	2.533	0.0485	209125_at	keratin 6A
RBKS	1.441	0.0486	219222_at	ribokinase
TNFSF10	-1.685	0.0486	202687_s_at	tumor necrosis factor (ligand) superfamily, member 10
MYRIP	-2.827	0.0487	214156_at	myosin VIIA and Rab interacting protein
RFWD2	0.294	0.0487	234950_s_at	ring finger and WD repeat domain 2, E3 ubiquitin protein ligase
DPM2	0.625	0.0487	209391_at	dolichyl-phosphate mannosyltransferase polypeptide 2, regulatory subunit
SPEF2	-0.545	0.0487	1552716_at	sperm flagellar 2
KIF18B	2.321	0.0487	222039_at	kinesin family member 18B
BMS1	0.439	0.0487	203082_at	BMS1 homolog, ribosome assembly protein (yeast)
PLAGL1	-1.256	0.0488	209318_x_at	pleiomorphic adenoma gene-like 1
EIF4E	-0.264	0.0489	242674_at	eukaryotic translation initiation factor 4E
OSMR	1.546	0.0489	205729_at	oncostatin M receptor
SERPINH1	1.177	0.0489	207714_s_at	serpin peptidase inhibitor, clade H (heat shock protein 47), member 1, (collagen binding protein 1)
SLC25A12	-0.675	0.049	203339_at	solute carrier family 25 (aspartate/glutamate carrier), member 12
LEO1	0.634	0.049	235096_at	Leo1, Paf1/RNA polymerase II complex component, homolog (S. cerevisiae)
HARBI1	0.62	0.049	241498_at	harbinger transposase derived 1
STC2	1.314	0.049	203439_s_at	stanniocalcin 2
NDRG2	-1.277	0.0491	206453_s_at	NDRG family member 2
HOXA3	-1.255	0.0491	235521_at	homeobox A3
DUSP7	-1.019	0.0492	213848_at	dual specificity phosphatase 7
DCTN4	0.712	0.0492	218013_x_at	dynactin 4 (p62)

PMS2P6	0.77	0.0492	215667_x_at	PMS2 postmeiotic segregation increased 2 (S. cerevisiae) pseudogene /// PMS2 postmeiotic segregation increased 2 (S. cerevisiae)-like /// postmeiotic segregation increased 2-like 2 pseudogene /// postmeiotic segregation increased 2 pseudogene 1 /// postme
GTF3A	0.534	0.0493	201338_x_at	general transcription factor IIIA
UBE2E1	-0.527	0.0496	212519_at	ubiquitin-conjugating enzyme E2E 1
CCL13	-1.539	0.0497	206407_s_at	chemokine (C-C motif) ligand 13
VEZF1	-0.414	0.0497	202173_s_at	vascular endothelial zinc finger 1
ATIC	1.1	0.0497	208758_at	5-aminoimidazole-4-carboxamide ribonucleotide formyltransferase/IMP cyclohydrolase
TRAK1	-0.415	0.0497	202079_s_at	trafficking protein, kinesin binding 1
HSPA9	0.68	0.0497	200692_s_at	heat shock 70kDa protein 9 (mortalin)
TTC7B	-0.961	0.0498	226152_at	tetratricopeptide repeat domain 7B
GSTM2	-1.602	0.0499	204418_x_at	glutathione S-transferase mu 2 (muscle)

s
