# Supplementary material for: Consistent gene expression profiles in MexTAg transgenic mouse and wild type mouse asbestos-induced mesothelioma
Source: BMC Cancer. 2015 Dec 18;15:983. doi: 10.1186/s12885-015-1953-y (PMC4683914; doi:10.1186/s12885-015-1953-y)
Supplement: Additional file 5: Table S5. — List of 211 differentially expressed genes from human array data that were common to the three mouse data sets. (PDF 70 kb) [file 12885_2015_1953_MOESM5_ESM.pdf]

Gene_A	logFC_WTtu_WTn	pval_WTtu_WTn	logFC_hiTAgtu_WTn	pval_hiTAgtu_WTn	logFC_loTAgtu_WTn	pval_loTAgtu_WTn	Desc
Abcb1a	-2.803	.000988	-2.772	.000839	-2.957	.000482	"ATP-binding
Actl6a	.940	.005930	1.408	.000089	1.503	.000050	actin-like
Agtr1a	-8.946	.000006	-8.226	.000010	-8.619	.000008	"angiotensin
Angptl1	-3.120	.017000	-2.548	.038200	-3.177	.011400	angiopoietin-like
Arhgef4	-1.178	.002990	-1.108	.003780	-1.317	.000852	Rho
Arid5b	-2.632	.005950	-1.730	.029900	-2.413	.008920	AT
Aspm	4.154	.000000	4.895	.000000	4.556	.000000	"asp
4933424B01Rik	.921	.022000	1.124	.003970	.940	.014800	"asunder
Atad2	2.922	.000047	3.751	.000002	3.487	.000005	"ATPase
Atxn7l1	-.177	.000130	-.177	.000096	-.175	.000127	ataxin
Bcl2l2	-.791	.031200	-.366	.021100	-.366	.020700	BCL2-like
Bms1	.992	.010600	1.038	.005750	.828	.023400	"BMS1
Brca2	2.680	.000824	3.563	.000029	2.673	.000697	breast
Bub1	3.686	.000002	4.481	.000000	4.267	.000000	budding
2010107G23Rik	-2.961	.002350	-2.451	.008330	-3.116	.001180	RIKEN
1500015O10Rik	-2.924	.004310	-2.993	.002700	-2.831	.004400	RIKEN
Cbx1	.885	.030200	.945	.015400	1.140	.003970	chromobox
Cbx5	1.512	.006900	3.034	.000518	1.155	.030300	chromobox
Ccbe1	-.979	.000425	-.978	.000329	-.906	.000746	collagen
Ccnb2	3.141	.000030	3.504	.000006	3.395	.000011	cyclin
Cd93	-.104	.038700	-.104	.029900	-.104	.030200	CD93
Cdc20	2.804	.000076	3.176	.000013	3.036	.000026	cell
Cdc6	4.181	.000003	5.031	.000000	4.368	.000001	cell
Cdk1	2.438	.000045	2.954	.000003	2.500	.000029	cyclin-dependent
Cdk2ap1	.860	.032000	.890	.020300	.853	.020600	CDK2
Cds2	-.896	.005410	-.875	.004940	-1.128	.000537	CDP-diacylglycerol
Cenpa	3.084	.000168	3.686	.000016	3.179	.000104	centromere
Cenpc1	.890	.048500	1.106	.011800	1.370	.002300	centromere
Cenpf	4.341	.000005	5.158	.000000	4.750	.000001	centromere
Cenph	3.156	.000006	3.798	.000000	3.608	.000001	centromere
Cenpn	3.866	.000005	4.655	.000000	3.641	.000010	centromere
Cgrrf1	-.697	.013800	-.794	.003910	-.754	.005960	cell
Chek1	3.508	.000195	4.475	.000009	4.319	.000017	checkpoint
Ckap2	1.821	.000927	2.344	.000047	1.582	.002680	cytoskeleton
Ckap5	1.225	.003170	1.697	.000091	1.051	.008300	cytoskeleton
Creb3	-.271	.000106	-.255	.000155	-.286	.000051	cAMP
Cse1l	1.380	.004460	1.539	.001310	1.692	.000560	chromosome
Cyb5r3	-1.959	.005730	-1.624	.003480	-2.428	.000075	cytochrome
Dctpp1	1.938	.038600	2.280	.010700	1.901	.031100	dCTP
Depdc1a	2.951	.000608	4.319	.000006	4.470	.000005	DEP
Depdc1b	2.503	.001270	3.716	.000013	2.624	.000661	DEP
Dna2	2.827	.000047	4.288	.000000	3.510	.000003	DNA
Dnajc9	1.410	.004040	2.176	.000037	1.629	.000881	"DnaJ
Dock8	-.103	.038700	-.103	.029900	-.103	.030200	dedicator
Dpy30	1.342	.007550	1.379	.004620	1.138	.018100	dpy-30
Dtymk	1.510	.007720	1.198	.027600	2.058	.000385	deoxythymidylate
E2f7	4.257	.000010	5.298	.000000	4.121	.000013	E2F
Ect2	2.990	.000064	4.172	.000001	3.812	.000003	ect2
Egfr	-.107	.038700	-.107	.029900	-.107	.030200	epidermal

2900064A13Rik	-2.430	.004130	-1.988	.014300	-1.791	.026800	ER
Emcn	-.889	.000000	-.889	.000000	-.889	.000000	endomucin
Eme1	3.056	.008150	3.472	.002180	2.658	.016600	essential
Epha3	-2.340	.022000	-2.458	.012200	-2.045	.034100	Eph
Eri2	1.722	.022100	2.041	.005070	2.220	.002620	exoribonuclease
Espl1	3.012	.000939	4.024	.000032	2.619	.002700	extra
Ezh2	2.322	.000021	3.192	.000000	3.005	.000001	enhancer
Fam13c	-2.650	.016700	-2.695	.011200	-2.830	.008140	"family
Fanca	3.037	.000063	4.002	.000002	2.486	.000429	"Fanconi
Fancd2	2.923	.000448	3.477	.000051	2.923	.000366	"Fanconi
Fancf	1.478	.024700	1.477	.019500	1.984	.002170	"Fanconi
Fanci	2.161	.004070	2.776	.000295	2.481	.000940	"Fanconi
Fen1	2.687	.000002	2.965	.000000	2.961	.000000	flap
Fgr	-.032	.042800	-.032	.034000	-.032	.034300	Gardner-Rasheed
Fmo2	-9.102	.000009	-9.211	.000006	-8.368	.000022	flavin
Fmo3	-5.031	.000111	-5.241	.000051	-4.021	.000908	flavin
Foxm1	4.260	.000015	5.767	.000000	3.485	.000126	forkhead
Foxp1	2.059	.003530	1.676	.013000	1.874	.010100	forkhead
Ggct	-.049	.039600	-.049	.031000	-.049	.031200	gamma-glutamyl
Gimap6	-.128	.044700	-.129	.033900	-.120	.047800	"GTPase
Gins1	3.550	.000120	4.179	.000013	3.993	.000028	GINS
Gins2	2.691	.000080	3.753	.000001	2.485	.000158	GINS
Gmcl1	1.869	.005310	.631	.029500	.687	.017900	germ
Gmnn	2.198	.000900	3.044	.000020	2.906	.000040	geminin
Gtf2h2	1.151	.035300	1.118	.030300	1.477	.005120	"general
Hat1	1.659	.004310	2.723	.000020	1.917	.000942	histone
Hells	4.127	.000000	5.097	.000000	4.577	.000000	"helicase
Hjurp	1.519	.000593	2.378	.000003	1.175	.004770	Holliday
Hmmr	3.135	.000029	3.620	.000004	3.664	.000004	hyaluronan
Hn1l	1.433	.005150	1.221	.012800	1.367	.005800	hematological
Hnmt	-.481	.003870	-.480	.002940	-.478	.003110	histamine
Hnrnpab	.934	.001430	.965	.000804	.946	.002010	heterogeneous
Hpgd	-6.768	.000000	-6.751	.000000	-6.020	.000001	hydroxyprostaglandin
Il1rap	-.035	.041700	-.035	.033000	-.035	.033200	interleukin
Ilf3	1.336	.038700	1.391	.024100	1.505	.014700	interleukin
Jam2	-2.808	.000001	-2.837	.000001	-2.821	.000001	junction
Etl4	-.723	.000000	-.740	.000000	-.755	.000000	enhancer
C330027C09Rik	3.267	.000035	4.359	.000001	3.664	.000008	RIKEN
Kif13b	-.197	.040400	-.196	.032700	-2.318	.013400	kinesin
Kif23	5.403	.000000	6.012	.000000	5.615	.000000	kinesin
Kif2c	3.918	.000006	4.319	.000001	3.996	.000004	kinesin
Lmbr1	-.684	.000163	-.704	.000087	-.642	.000264	limb
Lmcd1	-7.872	.001200	-7.345	.001760	-5.918	.010000	LIM
Lrpprc	1.324	.033200	1.525	.010300	1.323	.025700	leucine-rich
Lrr1	4.090	.000786	5.333	.000034	5.148	.000060	leucine
Lsm3	1.669	.005400	2.128	.000449	1.443	.012000	"LSM3
Lsm5	1.588	.001360	1.739	.000439	1.555	.001350	"LSM5
Mad2l1	2.374	.000002	2.869	.000000	2.690	.000000	MAD2
Mtap9	-.066	.040000	-.066	.031400	-.064	.037400	microtubule-associated
Mark1	-1.240	.022200	-3.416	.001180	-3.407	.001260	MAP/microtubule

Mcfd2	-1.333	.011800	-1.487	.003890	-1.448	.004930	multiple
Mcm2	2.627	.000008	4.035	.000000	2.674	.000011	minichromosome
Mcm4	2.270	.000036	3.213	.000000	3.497	.000000	minichromosome
Mcm6	2.685	.000005	3.762	.000000	3.315	.000001	"minichromosome
Mcm7	2.168	.000805	3.104	.000008	2.650	.000085	minichromosome
Melk	3.496	.000051	4.321	.000003	3.714	.000022	maternal
2610039C10Rik	1.765	.000301	3.065	.000000	2.663	.000002	MIS18
Mki67	3.274	.000015	3.662	.000003	3.389	.000009	antigen
Mphosph9	1.476	.023900	2.647	.000109	2.206	.000789	M-phase
Mrpl13	1.263	.001600	1.165	.002550	1.801	.000034	mitochondrial
Mrpl28	.841	.020200	1.575	.004020	1.023	.003730	mitochondrial
Naa38	1.317	.010700	2.094	.000107	1.943	.000266	"N(alpha)-acetyltransferase
Nampt	1.440	.045800	2.335	.001260	1.917	.006780	nicotinamide
Ncapd3	1.857	.005680	2.994	.000020	2.775	.000100	"non-SMC
Ndc80	3.345	.000005	4.100	.000000	4.060	.000000	"NDC80
Nmral1	2.354	.030900	3.061	.003740	2.677	.010400	NmrA-like
Nol7	.966	.022400	1.242	.002660	1.443	.000711	nucleolar
Nr3c2	-.908	.000000	-.897	.000000	-.903	.000000	"nuclear
Nsun3	-.520	.000026	-.544	.000011	-.491	.000042	NOL1/NOP2/Sun
Nucks1	.844	.000187	.894	.000074	.772	.000392	nuclear
Nup107	2.079	.000019	2.270	.000004	1.952	.000033	nucleoporin
Nup155	1.974	.000238	2.172	.000062	1.909	.000278	nucleoporin
Nup37	1.528	.013000	1.873	.001940	1.714	.004230	nucleoporin
Nup54	1.151	.004970	1.572	.000195	1.733	.000078	nucleoporin
Nusap1	3.128	.000009	4.247	.000000	4.112	.000000	nucleolar
Oip5	2.197	.000259	3.022	.000005	2.322	.000122	Opa
Pam	-.841	.017200	-1.365	.000184	-1.098	.001670	peptidylglycine
4930547N16Rik	2.915	.000047	3.088	.000017	3.077	.000022	PARP1
Pcna	1.708	.000018	2.519	.000000	2.189	.000001	proliferating
Pcnxl2	-4.383	.000000	-4.383	.000000	-4.355	.000000	pecanex-like
Pcsk5	-2.732	.000428	-2.575	.000593	-2.386	.001280	proprotein
Pdia3	-1.980	.001070	-2.043	.000615	-1.327	.019700	protein
Pdia6	-.193	.000481	-.193	.000370	-.192	.000410	protein
Pdpn	-1.905	.016400	-1.513	.042400	-1.759	.020600	podoplanin
Pdrg1	-1.459	.040400	-1.709	.013300	-2.094	.002920	p53
H2-Ke2	.931	.030900	1.133	.006310	1.031	.012400	H2-K
Plk1	3.362	.000117	3.649	.000034	2.604	.001260	polo-like
Pnpt1	1.239	.001200	.942	.009230	.906	.012000	polyribonucleotide
Podn	-.390	.017100	-.434	.006050	-.432	.006370	podocan
Pola1	3.538	.000259	5.035	.000003	4.389	.000021	"polymerase
Pold3	1.541	.038700	1.838	.001340	2.312	.001680	"polymerase
Ppih	2.315	.030800	2.438	.010000	2.528	.007930	peptidyl
Prkci	.855	.015200	1.797	.000031	.667	.044400	"protein
Prpf19	.786	.003230	.540	.031400	.658	.010000	PRP19/PSO4
Psmc3ip	2.241	.000084	2.694	.000007	2.641	.000034	"proteasome
Ptcd2	1.207	.009320	.966	.029900	1.321	.003570	pentatricopeptide
Ptdss2	-.107	.000232	-.124	.000033	-.124	.000039	phosphatidylserine
Pter	-1.698	.002720	-1.802	.001200	-1.583	.003850	phosphotriesterase
Ptplad1	-1.266	.018600	-1.036	.032600	-1.968	.000350	protein
Racgap1	3.637	.000010	4.484	.000001	3.633	.000010	Rac

Rad18	3.027	.000030	2.347	.000025	2.013	.000161	RAD18
Rad21	1.217	.000927	1.674	.000159	1.863	.000008	RAD21
Rad50	1.214	.003360	.926	.019300	1.178	.003400	RAD50
Ran	.858	.003900	.942	.000697	.983	.000908	"RAN
Rapgef3	-1.161	.001600	-1.324	.000349	-1.359	.000281	Rap
Rbm14	1.348	.027300	1.982	.001050	1.516	.009750	RNA
Rell1	.847	.033600	2.043	.000007	1.743	.000051	RELT-like
Rfc4	2.334	.000106	3.648	.000000	3.172	.000003	replication
Rfc5	3.052	.000006	3.795	.000000	2.689	.000022	replication
Rhobtb1	-2.343	.007820	-2.460	.004040	-1.638	.049000	Rho-related
Samd4b	-1.879	.005940	-1.511	.021300	-2.313	.000761	sterile
Scamp1	-1.338	.025600	-1.463	.010900	-1.387	.015800	secretory
Selp	-4.438	.000000	-4.441	.000000	-4.441	.000000	"selectin
Set	1.172	.007450	1.210	.004390	1.362	.001670	SET
Setdb1	1.027	.029000	1.762	.000252	1.481	.001450	"SET
Setdb2	1.309	.012100	2.695	.000007	2.011	.000210	"SET
Shcbp1	3.400	.000031	4.015	.000003	3.915	.000005	Shc
Shfm1	.719	.011800	.635	.020900	.865	.002140	split
Sirt2	-1.113	.003010	-.931	.009360	-1.559	.000087	"sirtuin
Skp2	2.856	.000254	3.473	.000021	3.946	.000108	S-phase
Slc16a9	-4.760	.000000	-5.092	.000000	-5.092	.000000	"solute
Slc44a1	-1.779	.007040	-1.867	.003630	-1.350	.031000	"solute
Slk	1.319	.030900	1.407	.010600	1.344	.014600	STE20-like
Smc2	2.427	.000100	3.089	.000004	2.815	.000017	structural
Smc4	1.533	.020300	2.750	.000080	2.299	.001120	structural
Snrpd1	1.013	.004280	1.635	.000024	1.151	.001090	small
Snrpe	1.079	.002510	1.146	.001100	1.373	.000196	small
Sox17	-.088	.038700	-.088	.030000	-.088	.030300	SRY-box
Spag5	4.387	.000000	4.797	.000000	4.127	.000000	sperm
Spc25	2.351	.000028	2.650	.000005	2.824	.000003	"SPC25
Spock2	-5.212	.000000	-5.128	.000000	-5.243	.000000	"sparc/osteonectin
Spry4	-3.103	.000162	-2.949	.000209	-2.841	.000334	sprouty
Spsb1	-3.225	.000000	-3.112	.000000	-3.226	.000000	splA/ryanodine
St6galnac3	-.124	.038700	-.124	.030100	-.124	.030300	"ST6
Stil	2.358	.005680	3.282	.000190	2.259	.006120	Scl/Tal1
Stmn1	1.710	.001500	2.427	.000028	1.670	.001520	stathmin
Stom	-2.958	.018900	-2.117	.019600	-3.157	.009290	stomatin
Suv39h2	3.237	.010600	4.483	.000472	3.211	.008680	suppressor
Tardbp	.932	.025100	1.112	.005670	1.130	.005120	TAR
Tcf19	2.202	.010000	3.608	.000070	2.317	.005250	transcription
Tcf3	1.653	.000045	1.548	.000067	.720	.036600	transcription
Tfdp1	1.203	.004450	1.336	.001350	.828	.037300	transcription
Timeless	3.784	.000018	5.041	.000000	3.939	.000010	timeless
Tmpo	2.403	.000024	3.064	.000001	2.865	.000003	thymopoietin
Top2a	5.786	.000000	5.150	.000000	5.211	.000000	topoisomerase
Trim27	.943	.011400	1.012	.005080	.700	.046800	tripartite
Trim37	1.192	.017000	1.606	.001170	1.398	.004030	tripartite
Trip13	2.539	.000135	3.225	.000006	2.880	.000029	thyroid
1190005F20Rik	-.161	.043400	-.167	.029900	-.168	.030200	tRNA
Troap	3.490	.007120	4.489	.000577	3.032	.014800	trophinin

Ttk	4.461	.000001	5.071	.000000	5.282	.000000	Ttk
Tyms	2.620	.001240	3.099	.000174	2.681	.000813	thymidylate
Ube2c	2.633	.000786	3.708	.000014	2.317	.002080	ubiquitin-conjugating
Ube2t	3.088	.000006	4.031	.000000	3.296	.000002	ubiquitin-conjugating
Ubxn4	-.631	.036500	-.586	.040500	-1.031	.000648	UBX
Ung	3.439	.039500	4.929	.002700	3.572	.026800	uracil
Usp14	.921	.010100	1.060	.002480	.959	.005780	ubiquitin
Wdr74	1.563	.001240	1.258	.006210	1.092	.016800	WD
Xpo1	1.233	.000598	1.560	.000034	1.604	.000030	"exportin
Ypel2	-1.758	.000005	-1.758	.000004	-1.756	.000005	yippee-like
Zfp36	-3.604	.003880	-2.762	.020600	-2.863	.016300	zinc
Zwilch	3.161	.000011	3.267	.000005	3.408	.000004	zwilch
